# Supplementary material for: β‐Catenin activity induces an RNA biosynthesis program promoting therapy resistance in T‐cell acute lymphoblastic leukemia
Source: EMBO Mol Med. 2023 Jan 4;15(2):e16554. doi: 10.15252/emmm.202216554 (PMC9906382; doi:10.15252/emmm.202216554)
Supplement: Supplementary file 1 — Appendix [file EMMM-15-e16554-s004.pdf]

**APPENDIX**

**Table of content:**

Appendix Table S1 – sg RNA
Appendix Table S2 – primers
Appendix Table S3 – antibodies
Appendix Table S4 – antibodies for flow cytometry analysis
Appendix Table S5- p values for Fig 5 and Fig EV5

Appendix Table S1 - sgRNA

| Target | Guide | sgRNA |
|---|---|---|
| TCF7 (TCF1) | 1 | TCGCTCGTGAACGAGTCCGA |
| | 2 | ATGCCGCAGCTGGACTCCGG |
| LEF1 | 1 | CGAGATCAGTCATCCCGAAG |
| | 2 | ATGATCCCCTTCAAGGACGA |
| ZBTB33 | 1 | GCTACAGACATTCAGTACTC |
| | 2 | AAGCATCTCAGGTACAGCGC |

Appendix Table S2 - primers

| Application | Target | Primer Forward | Primer Reverse |
|---|---|---|---|
| ChIP-qPCR | CDK12 | TGATAAGCAGGGGAATGAGG | CCTCCACACCACACACACTC |
| | DDX20 | ACCAGTTGCCTCATCTTTCC | TGGGAGCGAGAAGAAGAAAA |
| | RBM39 | AAAACTGATCCCATGCCAAG | TCTTTAGGGGTAGGGGGAGA |
| | RPL10A | TCAGGACTGCGACAACTCTG | AGGACCAACTCACCTCATGG |
| | POLR1C | GAGACGGAGCAGGGCTACTA | CTCAAGGCCACAAGGCTTAC |
| | POLR2E | CCTGAATGCAGCAACGTCT | GTGGGAGTTGAACGGAAACT |
| Expression | CTNNB1/ β-Catenin | CGGAGACGGAGGAAGGTCTG | TCCATCAAATCAGCTTGAGTAGC |
| | ZBTB33/Kaiso | GTGCGGCTTCTTCTTGTTGG | TCCATGCCCTTTCCTCTTTCT |
| | RPS19 | AGACGTGAACCAGCAGGAGT | TTCTCTGACGTCCCCCATAG |
| | RPL12 | CCTGGGTCTGTCTCCAAAAA | GTTCCTTGAGGGCTTTGATG |
| | RPL10A | TGGAGTTGCAGATCAGCTTG | GGCCAAAAACGCATCATACT |
| | POLR1C | CCAGGCTGATCTCTTTCCAG | TCTGGCAGGAGCCTGTAACT |
| | MATR3 | CGAAGCAGCGTTGGAAAATG | CATCACTTTGACCATCTGCG |
| | TBP | TGCCCGAAACGCCGAATATAATC | GTCTGGACTGTTCTTCACTCTTGG |
| | HPRT1 | ATAAGCCAGACTTTGTTGG | ATAGGACTCCAGATGTTTCC |

Appendix Table S3 - antibodies

| Application | Target | Source | Reference | Host specie | µg or dilution |
|---|---|---|---|---|---|
| ChIP | β-Catenin | R&D Systems | AF1329 | Goat | 2.5 µg |
| | β-Catenin | Santa Cruz | sc-7199 | Rabbit | 10 µg |
| | TCF1/TCF7 | Cell Signaling | 2206S | Rabbit | 1/50 |
| | LEF1 | Abcam | ab137872 | Rabbit | 2.5 µg |
| | Kaiso | Santa Cruz | sc-23871 X | Mouse | 10 µg |
| | acetyl-Histone H3 | Upstate | 06-599 | Rabbit | 5 µg |
| WB | β-Catenin | BD Biosciences | 610154 | Mouse | 1/1000 |
| | Active-β-Catenin | Millipore | 05-665 | Mouse | 1/1000 |
| | Kaiso | Bethyl Laboratories | A303-558A | Rabbit | 1/1000 |
| | TCF1/TCF7 | Cell Signaling | 2206S | Rabbit | 1/1000 |
| | LEF1 | Abcam | ab137872 | Rabbit | 1/1000 |
| | Tubulin | Sigma | T6074 | Mouse | 1/10000 |
| | Histone H3 | Abcam | ab1791 | Rabbit | 1/10000 |

Appendix Table S4 – antibodies for flow cytometry analysis

| Target | Fluorochrome | Source | Reference | Clone | Target specie | Dilution |
|---|---|---|---|---|---|---|
| CD45 | PerCP Cy5.5 | Invitrogen | 45-0451-82 | 30-F11 | Mouse | 1/200 |
| CD45 | APC H7 | BD Biosciences | 560178 | 2D1 | Human | 1/200 |
| HLA-ABC | FITC | BD Biosciences | 555552 | G46-2.6 | Human | 1/200 |
| CD5 | PE | Biolegend | 300622 | UCHT2 | Human | 1/200 |
| Ki67 | AF647 | BD Biosciences | 558615 | B56 | Human | 1/100 |

# Appendix Table S5

| Figure (panel) | Comparison | p-value | p-value summary | Statistical test | Previous test | p-value (previous test) |
|---|---|---|---|---|---|---|
| Fig 5a | sh β-cat + VCR vs sh control + VCR | 0.0004 | *** | Two-sided Student's t test | | |
| Fig 5b | VCR + DMSO vs VCR + ICG-001 0.5 µM | 0.0025 | ** | Two-sided Student's t test | | |
| Fig 5b | VCR + DMSO vs VCR + ICG-001 1 µM | 0.0002 | *** | Two-sided Student's t test | | |
| Fig 5b | VCR + DMSO vs VCR + ICG-001 2.5 µM | <0.0001 | **** | Two-sided Student's t test | | |
| Fig 5b | VCR + DMSO vs VCR + ICG-001 10 µM | <0.0001 | **** | Two-sided Student's t test | | |
| Fig 5c (left) | MTX + DMSO vs MTX + ICG-001 0.5 µM | 0.6313 | n.s. | Two-sided Student's t test | | |
| Fig 5c (left) | MTX + DMSO vs MTX + ICG-001 1 µM | 0.0310 | * | Two-sided Student's t test | | |
| Fig 5c (left) | MTX + DMSO vs MTX + ICG-001 2.5 µM | 0.0006 | *** | Two-sided Student's t test | | |
| Fig 5c (left) | MTX + DMSO vs MTX + ICG-001 10 µM | 0.0208 | * | Two-sided Student's t test | | |
| Fig 5c (central) | L-ASP + DMSO vs L-ASP + ICG-001 0.5 µM | 0.0002 | *** | Two-sided Student's t test | | |
| Fig 5c (central) | L-ASP + DMSO vs L-ASP + ICG-001 1 µM | <0.0001 | **** | Two-sided Student's t test | | |
| Fig 5c (central) | L-ASP + DMSO vs L-ASP + ICG-001 2.5 µM | <0.0001 | **** | Two-sided Student's t test | | |
| Fig 5c (central) | L-ASP + DMSO vs L-ASP + ICG-001 10 µM | 0.0002 | *** | Two-sided Student's t test | | |
| Fig 5c (right) | Ara-C + DMSO vs Ara-C + ICG-001 0.5 µM | 0.0063 | ** | Two-sided Student's t test | | |
| Fig 5c (right) | Ara-C + DMSO vs Ara-C + ICG-001 1 µM | 0.0034 | ** | Two-sided Student's t test | | |
| Fig 5c (right) | Ara-C + DMSO vs Ara-C + ICG-001 2.5 µM | 0.0022 | ** | Two-sided Student's t test | | |
| Fig 5c (right) | Ara-C + DMSO vs Ara-C + ICG-001 10 µM | 0.0014 | ** | Two-sided Student's t test | | |
| Fig 5e (2 days after treatment initiation) | VCR vs untreated | <0.0001 | **** | Tukey's multiple comparisons test | One-way ANOVA | <0.0001 |
| Fig 5e (2 days after treatment initiation) | VCR + ICG-001 10 µM vs untreated | <0.0001 | **** | Tukey's multiple comparisons test | One-way ANOVA | <0.0001 |
| Fig 5e (2 days after treatment initiation) | VCR vs VCR + ICG-001 10 µM | 0.0007 | ### | Tukey's multiple comparisons test | One-way ANOVA | <0.0001 |
| Fig 5f (3 days from drug wash-out) | VCR 15 nM, recovery in ICG-001 vs recovery in complete medium | <0.0001 | **** | Two-sided Student's t test | | |
| Fig 5f (8 days from drug wash-out) | VCR 15 nM, recovery in ICG-001 vs recovery in complete medium | 0.0006 | *** | Two-sided Student's t test | | |
| Fig 5f (3 days from drug wash-out) | VCR 15 nM + ICG-001 10 µM, recovery in ICG-001 vs recovery in complete medium | 0.0011 | ** | Two-sided Student's t test | | |
| Fig 5f (8 days from drug wash-out) | VCR 15 nM + ICG-001 10 µM, recovery in ICG-001 vs recovery in complete medium | 0.0217 | * | Two-sided Student's t test | | |
| Fig 5h (0 days after treatment initiation) | sh β-cat untreated vs sh control untreated | 0.3554 | n.s. | Two-sided Student's t test | | |
| Fig 5h (2 days after treatment initiation) | sh β-cat untreated vs sh control untreated | <0.0001 | **** | Two-sided Student's t test | | |
| Fig 5h (2 days after treatment initiation) | sh β-cat VCR 1 nM vs sh control 1 nM | <0.0001 | #### | Two-sided Student's t test | | |
| Fig 5h (2 days after treatment initiation) | sh β-cat VCR 15 nM vs sh control 15 nM | <0.0001 | ++++ | Two-sided Student's t test | | |
| Fig 5i (3 days from drug wash-out) | VCR 1 nM, sh β-cat vs sh control | <0.0001 | **** | Two-sided Student's t test | | |
| Fig 5i (8 days from drug wash-out) | VCR 1 nM, sh β-cat vs sh control | 0.0058 | ** | Two-sided Student's t test | | |
| Fig 5i (3 days from drug wash-out) | VCR 15 nM, sh β-cat vs sh control | 0.0009 | *** | Two-sided Student's t test | | |
| Fig 5i (8 days from drug wash-out) | VCR 15 nM, sh β-cat vs sh control | <0.0001 | **** | Two-sided Student's t test | | |
