# Supplementary material for: β‐Catenin activity induces an RNA biosynthesis program promoting therapy resistance in T‐cell acute lymphoblastic leukemia
Source: EMBO Mol Med. 2023 Jan 4;15(2):e16554. doi: 10.15252/emmm.202216554 (PMC9906382; doi:10.15252/emmm.202216554)
Supplement: Supplementary file 2 — Expanded View Figures PDF [file EMMM-15-e16554-s013.pdf]

Expanded View Figures

Figure EV1. β-catenin-binding genes in T-ALL cell lines.

A, B WB analysis of the indicated proteins from cytoplasmic and nuclear fractions of four different T-ALL cell lines (A) and at different times of 25 mM LiCl treatment in RPMI8402 cells (B). Tubulin and H3 detection were used as loading and fractionation controls.

C WB analysis of precipitated β-catenin in RPMI8402 cells crosslinked with 0.2 mM DSG at different time points, as done in the ChIP protocol.

D Venn diagrams showing the overlap between β-catenin-enriched ChIP-targets in basal vs LiCl treated conditions obtained in at least two replicates.

E IGV representation of β-catenin-enriched peaks in five basal and two LiCl treated replicates.

F Functional enrichment analysis of β-catenin ChIP targets. Statistically enriched categories (adjusted *P*-value < 0.05) among GO Biological Process terms.

G qPCR detection of β-catenin-enriched genes by ChIP in basal and LiCl treated conditions in the RPMI8402 (left panel) and in the Jurkat (right panel) cell lines. qPCR results normalized to negative IgG control indicated by a red-dashed line. Graph represents the mean and SD of three technical replicates.

H Venn diagrams showing the overlap between the β-catenin, TCF1 and LEF1 ChIPenriched genes (left panel) or the overlap between the β-catenin and the Kaiso genes obtained in at least two replicates.

I WB analysis of Kaiso, TCF1 and LEF1 in control (Empty Vector, EV) and Kaiso KO clones (left panel) or KO clones for the long isoforms of TCF1 and LEF1 (DLKO1 and DLKO2) (right panel) in RPMI8402 cells. Cytoplasmic and nuclear fractions are analyzed and tubulin and H3 used as loading controls.

Source data are available online for this figure.

 © 2023 The Authors

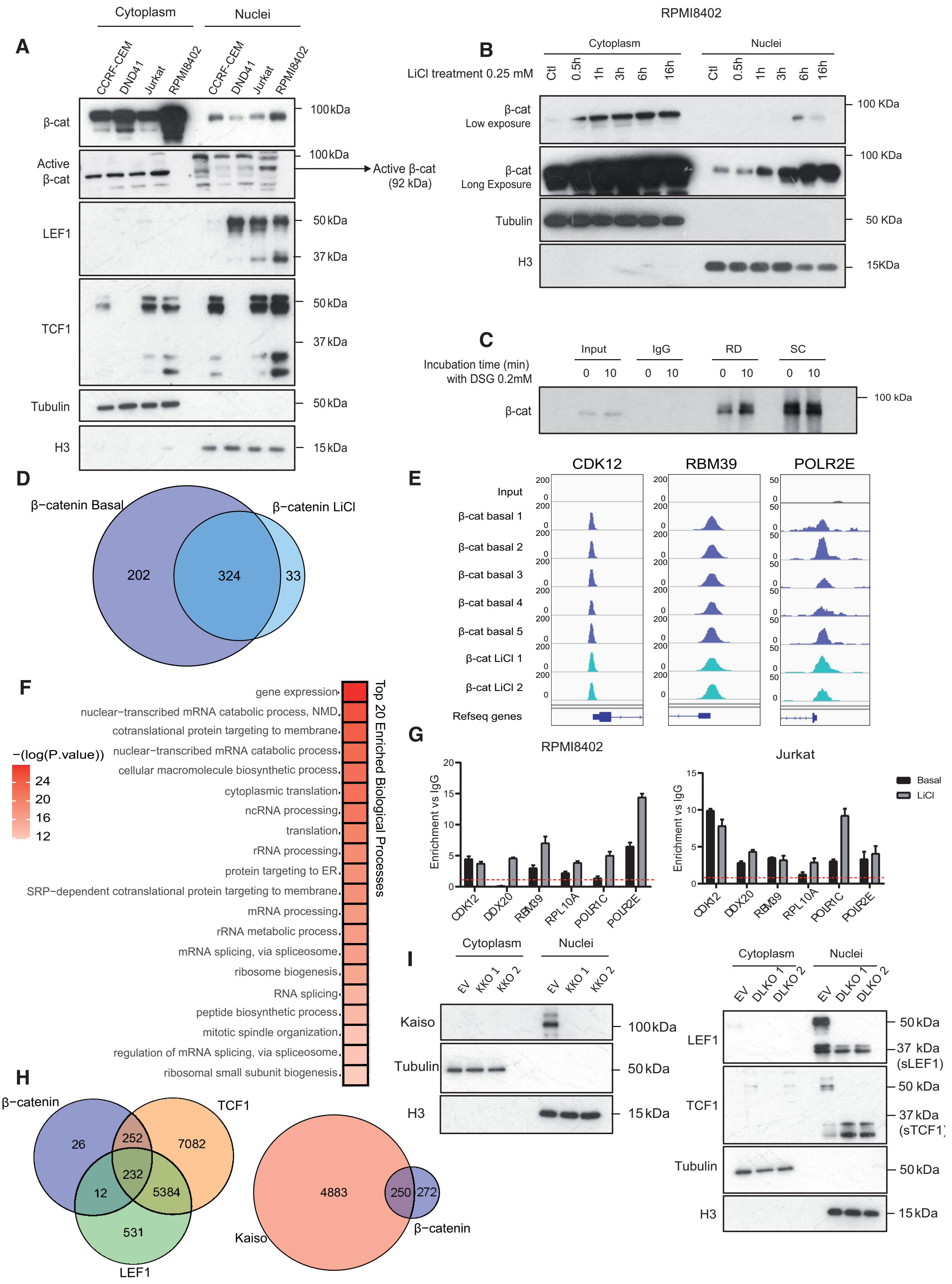
A
Cytoplasm
Nuclei
CCRF-CEM
DND41
Jurkat
RPMI8402
β-cat
Active β-cat
LEF1
TCF1
Tubulin
H3
100 kDa
50 kDa
37 kDa
15 kDa
Active β-cat (92 kDa)
B
RPMI8402
LiCl treatment 0.25 mM
Ctl
0.5h
1h
3h
6h
16h
β-cat Low exposure
β-cat Long Exposure
100 KDa
50 KDa
15KDa
C
Incubation time (min) with DSG 0.2mM
Input
IgG
RD
SC
0
10
100 kDa
D
β-catenin Basal
β-catenin LiCl
202
324
33
E
CDK12
RBM39
POLR2E
Input
β-cat basal 1
β-cat basal 2
β-cat basal 3
β-cat basal 4
β-cat basal 5
β-cat LiCl 1
β-cat LiCl 2
Refseq genes
F
−(log(P.value))
Top 20 Enriched Biological Processes
gene expression
nuclear-transcribed mRNA catabolic process, NMD
cotranslational protein targeting to membrane
nuclear-transcribed mRNA catabolic process
cellular macromolecule biosynthetic process
cytoplasmic translation
ncRNA processing
translation
rRNA processing
protein targeting to ER
SRP-dependent cotranslational protein targeting to membrane
mRNA processing
rRNA metabolic process
mRNA splicing, via spliceosome
ribosome biogenesis
RNA splicing
peptide biosynthetic process
mitotic spindle organization
regulation of mRNA splicing, via spliceosome
ribosomal small subunit biogenesis
G
RPMI8402
Jurkat
Enrichment vs IgG
Basal
LiCl
CDK12
DDX20
RBM39
RPL10A
POLR1C
POLR2E
H
β-catenin
TCF1
LEF1
26
252
7082
232
12
5384
531
4883
250
272
Kaiso
I
EV
KKO 1
KKO 2
DLKO 1
DLKO 2
Kaiso
37 kDa (sLEF1)
37 kDa (sTCF1)

Figure EV1.

Figure EV2. β-catenin-target gene expression in T-ALL cell lines.

A Representative heatmap of H3Ac enriched TSS-centered peaks in RPMI8402 cells in basal conditions and treated with LiCl 25 mM for 16 h (left panels) or H3K27Ac, H3K4me3 and H3K27me3 in RPMI8402, Jurkat or DND41 cell lines in basal conditions (right panels). Upper panels show β-catenin-binding targets and bottom panels show random genomic regions. Upper linear graphs show enrichment of the H3Ac mark in the TSS in the β-catenin-binding genes (blue) and in the random genomic regions (green).

B WB analysis of β-catenin levels in the cytoplasm, nucleoplasm and chromatin after treatment of RPMI8402 (left) or Jurkat cells (right) with β-catenin inhibitors ICG-001 or FH535 for 16 h at the indicated concentrations. Tubulin and H3 were used as loading controls.

C qPCR analysis of mRNA expression of β-catenin target genes in the RPMI8402 cell line after treatment with β-catenin inhibitor FH535 (30 μM, overnight). Graph represents the mean and SD of three independent experiments. Statistical significance was determined by two-sided Student's *t*-test.

D, E Flow cytometry cell cycle analysis of RPMI8402 cells after β-catenin inhibition (D) or knockdown (E). Top panels show representative flow cytometry density plots of cell cycle status. Bottom panels show the quantification of cell cycle phases distribution determined by DAPI and Ki67 incorporation in two independent experiments. Data represent the individual values per experiment and condition.

Source data are available online for this figure.

 © 2023 The Authors

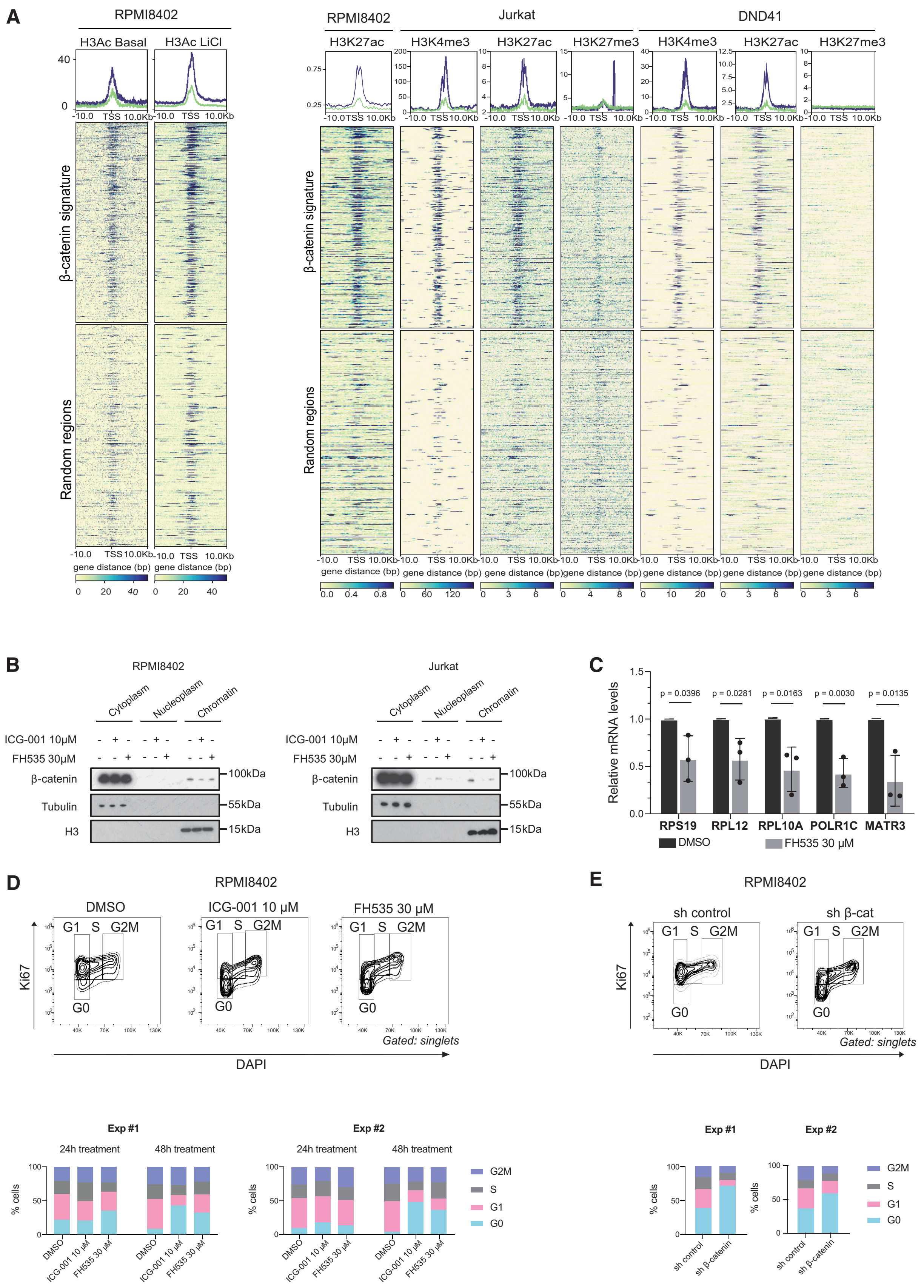
A
RPMI8402
H3Ac Basal
H3Ac LiCl
Jurkat
DND41
H3K27ac
H3K4me3
H3K27me3
β-catenin signature
Random regions
gene distance (bp)
B
RPMI8402
Jurkat
Cytoplasm
Nucleoplasm
Chromatin
ICG-001 10μM
FH535 30μM
β-catenin
Tubulin
H3
100kDa
55kDa
15kDa
C
Relative mRNA levels
p = 0.0396
p = 0.0281
p = 0.0163
p = 0.0030
p = 0.0135
RPS19
RPL12
RPL10A
POLR1C
MATR3
DMSO
FH535 30 μM
D
RPMI8402
DMSO
ICG-001 10 μM
FH535 30 μM
G1 S G2M
G0
Ki67
DAPI
Gated: singlets
E
sh control
sh β-cat
Exp #1
Exp #2
24h treatment
48h treatment
% cells
G2M
S
G1
G0
sh β-catenin

Figure EV2.

Figure EV3. β-catenin-signature in T-ALL.

A Non-supervised hierarchical classification of T-ALL patients (GSE14618, samples with available survival data $N = 40$) according to the expression of the 156 β-catenin targets found differentially expressed (DEG) in the RNAseq from sh-β-catenin. T-ALL phenotype, patient outcome, vital status and age are depicted on the top of the heatmap. Genes are represented on the left side and patients are shown in the upper part. A summary of the most representative biological functions significantly enriched for each group of genes is shown on the left (Gene Ontology Biological functions significantly enriched (adjusted P-value < 0.05) in groups of genes GA ($N = 92$), GB ($N = 18$) and GC ($N = 38$)). ETP, Early T-cell precursor; ABD, absence of biallelic TCRgamma locus deletion.

B Kaplan–Meier curves representing disease free (DSF, left) and overall survival (OS, right) probability for groups of patients PA ($N = 8$), PB ($N = 8$) and PC ($N = 24$) obtained in (A). Time is represented in years. Statistical significance among groups was determined by log-rank test.

C Scaled β-catenin, ZBTB33/Kaiso, TCF1 and LEF1 expression in each patient cluster PA ($N = 8$), PB ($N = 8$) and PC ($N = 24$). Box and whiskers plot represents the median (central bar), Q1 and Q4 quartiles (low and high hinge, respectively) and minimum and maximum values (lower and higher whisker, respectively) of the indicated scaled expressions. Statistical significance among groups was determined by Kruskal–Wallis test. When significant, Kruskal–Wallis is followed by Wilcoxon test for pairwise comparisons.

D Scaled β-catenin, ZBTB33/Kaiso, TCF1 and LEF1 expression in refractory ($N = 6$), relapse ($N = 13$) and remission cases ($N = 21$). Box and whiskers plot represents the median (central bar), Q1 and Q4 quartiles (low and high hinge, respectively) and minimum and maximum values (lower and higher whisker, respectively) of the indicated scaled expressions. Statistical significance among groups was determined by Kruskal–Wallis test. When significant, Kruskal–Wallis is followed by Wilcoxon test for pairwise comparisons.

E DFS probability for patients based solely on β-catenin expression. In the left panel, the high β-catenin group comprises patients with a β-catenin expression higher than the median ($N = 20$ per group). In the right panel, groups are established based on scaled expression quartiles (high β-catenin, Q4 ($N = 10$); medium β-catenin, Q3 ($N = 10$); and low β-catenin, Q1 + Q2 ($N = 20$). Statistical significance among groups was determined by log-rank test.

F–H DFS probability for patients based solely on Kaiso (F), TCF1 (G), LEF1 (H) expression. High factor groups comprise patients with a factor expression higher than the median ($N = 20$ per group). Statistical significance among groups was determined by log-rank test.

© 2023 The Authors

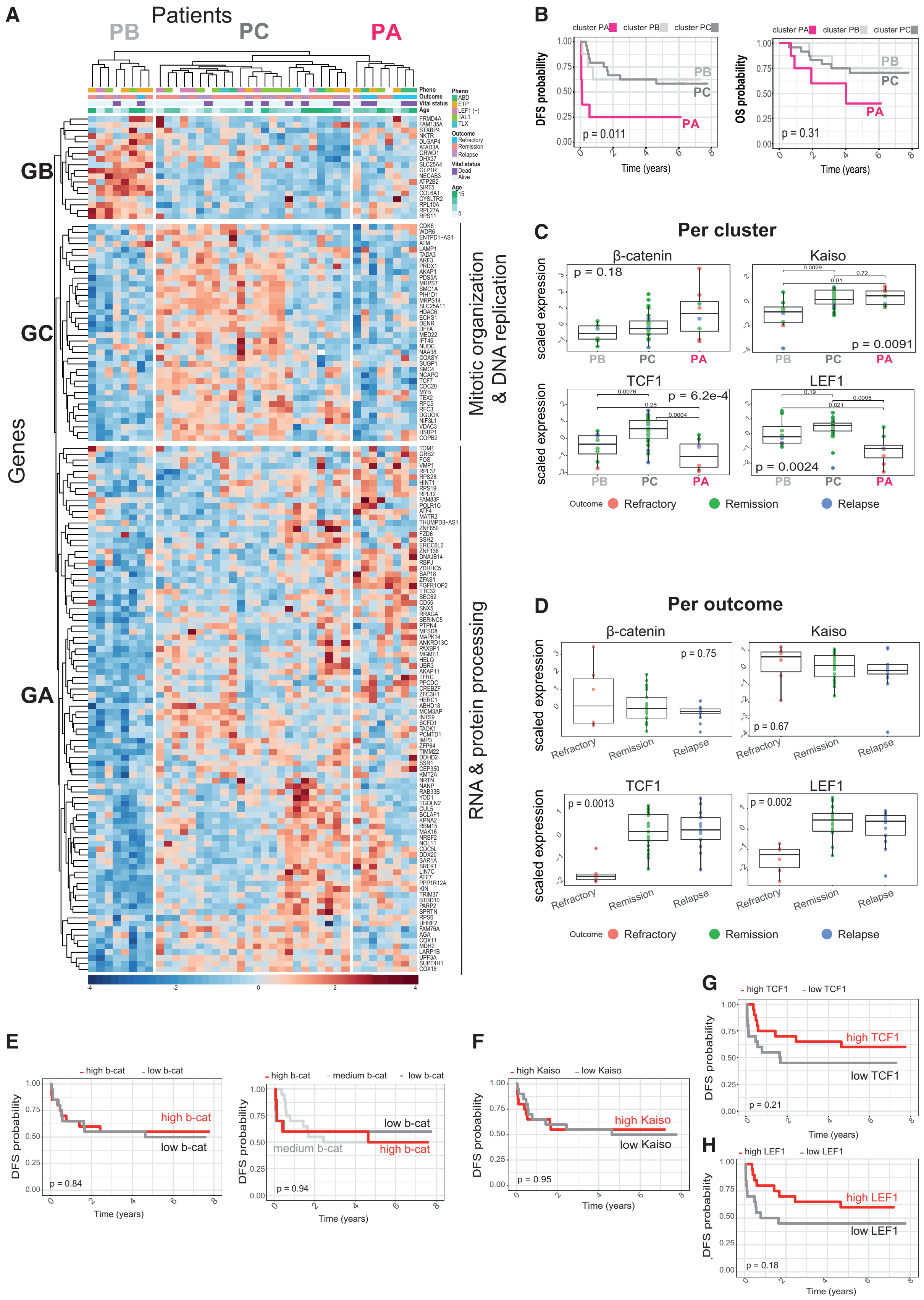
A
Patients
PB
PC
PA
GB
GC
GA
Genes
Mitotic organization & DNA replication
RNA & protein processing
B
cluster PA
cluster PB
cluster PC
DFS probability
OS probability
Time (years)
p = 0.011
p = 0.31
C
Per cluster
β-catenin
Kaiso
TCF1
LEF1
scaled expression
p = 0.18
p = 0.0091
p = 6.2e-4
p = 0.0024
Outcome
Refractory
Remission
Relapse
D
Per outcome
p = 0.75
p = 0.67
p = 0.0013
p = 0.002
E
high b-cat
low b-cat
medium b-cat
p = 0.84
p = 0.94
F
high Kaiso
low Kaiso
p = 0.95
G
high TCF1
low TCF1
p = 0.21
H
high LEF1
low LEF1
p = 0.18

Figure EV3.

Figure EV4. Minimal β-catenin-signature in T-ALL.

A Kaplan–Meier curves representing DSF (left) and OS (right) probability for groups of patients from the discovery cohort clustered according to the expression of the 77 β-catenin targets differentially upregulated in the RNAseq from sh-β-catenin. Time is represented in years. Statistical significance among groups was determined by log-rank test.

B Summary of β-catenin targets differentially expressed between refractory (*N* = 6) and remission (*N* = 21) cases from GSE14618 (samples with available survival data) and summary of the top Gene Ontology Biological functions enriched in groups of β catenin targets upregulated (*N* = 25) or downregulated (*N* = 17; *P*-value < 0.05, Dataset EV3).

C Classification of T-ALL patients (GSE14618, samples with available survival data *N* = 40) according to the expression of the 42 β-catenin targets differentially expressed (*P*-value < 0.05) between refractory and remission patients. T-ALL phenotype, patient outcome, vital status and age are depicted on the top of the heatmap. Genes are represented on the left side and patients are shown in the upper part.

D β-catenin expression levels in patients divided per outcome (left panels) or cluster (right panels) from the subset of GSE14618 cohort with no survival data available (left), EGA cohort (middle) and TARGET cohort (right). Q1, Q2–Q3, and Q4 clusters established according to G1 ssGSEA quartiles from Fig 4B–D. Box and whiskers plot represents the median (central bar), Q1 and Q4 quartiles (low and high hinge, respectively) and minimum and maximum values (lower and higher whisker, respectively) of the indicated expression. Statistical significance among groups was determined by Kruskal–Wallis test. For GSE14614 cohort with no survival data (left panel), Q1 *N* = 11, Q2–Q3 *N* = 20, Q4 *N* = 11. For EGA cohort (middle panel), Q1 *N* = 4, Q2–Q3 *N* = 14, Q4 *N* = 9. For TARGET cohort (right panel), Q1 = 63, Q2–Q3 = 129, Q4 = 61 (D).

E, F Kaplan–Meier curves representing DFS (upper panel) and OS (lower panel) probability for group of patients included in Q4 from Fig 4, compared with the other patients (not Q4) from the discovery cohort (E) and TARGET cohort (F). Q4 *N* = 11 (in D) or *N* = 61 (in E), not Q4 *N* = 31 (in D) or *N* = 192 (in E). Statistical significance among groups was determined by log-rank test.

 © 2023 The Authors

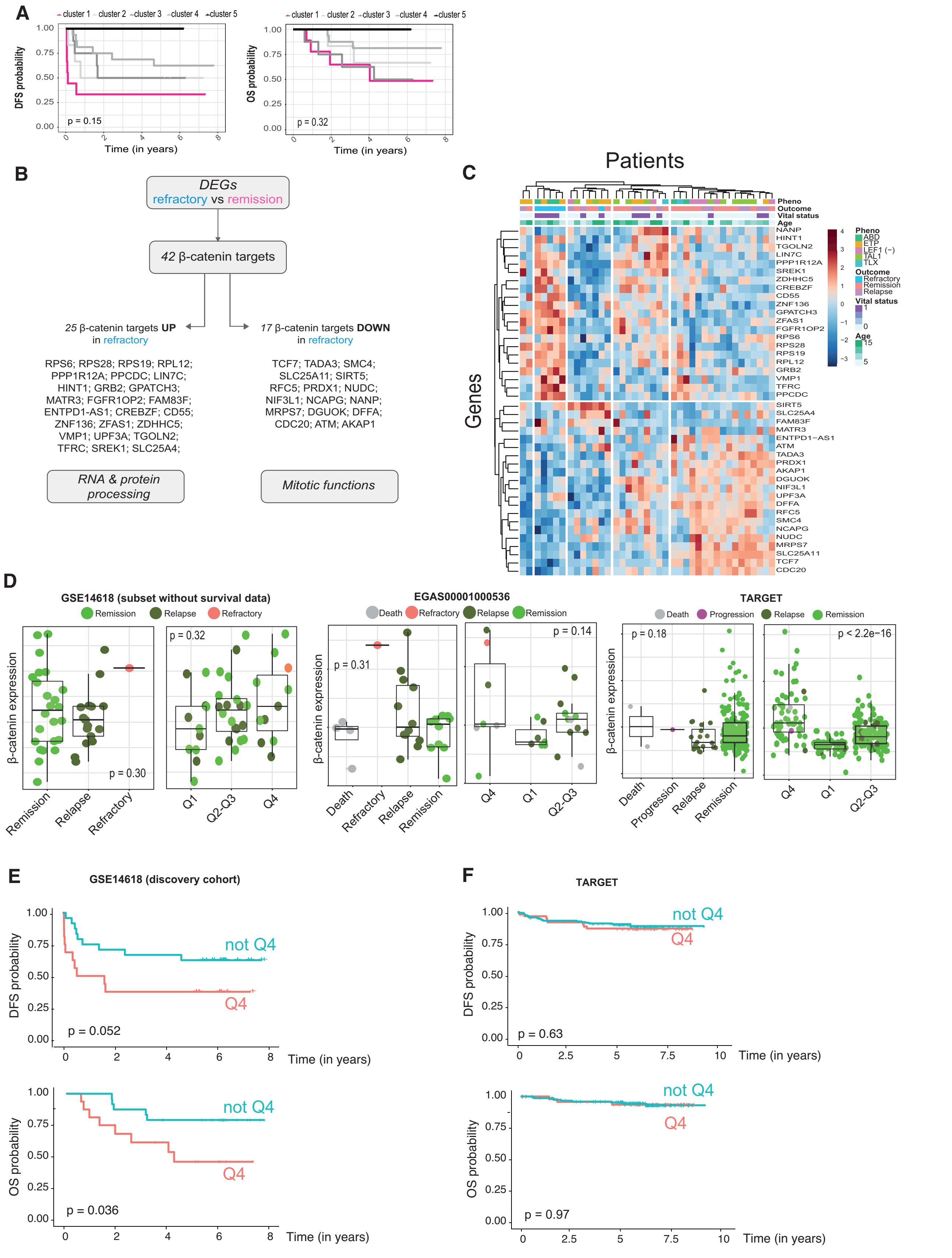
A
DFS probability
OS probability
Time (in years)
p = 0.15
p = 0.32
B
DEGs
refractory vs remission
42 β-catenin targets
25 β-catenin targets UP in refractory
17 β-catenin targets DOWN in refractory
RPS6; RPS28; RPS19; RPL12; PPP1R12A; PPCDC; LIN7C; HINT1; GRB2; GPATCH3; MATR3; FGFR1OP2; FAM83F; ENTPD1-AS1; CREBZF; CD55; ZNF136; ZFAS1; ZDHHC5; VMP1; UPF3A; TGOLN2; TFRC; SREK1; SLC25A4;
TCF7; TADA3; SMC4; SLC25A11; SIRT5; RFC5; PRDX1; NUDC; NIF3L1; NCAPG; NANP; MRPS7; DGUOK; DFFA; CDC20; ATM; AKAP1
RNA & protein processing
Mitotic functions
C
Patients
Genes
D
GSE14618 (subset without survival data)
EGAS00001000536
TARGET
β-catenin expression
E
GSE14618 (discovery cohort)
p = 0.052
p = 0.036
F
TARGET
p = 0.63
p = 0.97
not Q4
Q4

Figure EV4.

Figure EV5. β-catenin effect in chemotherapy cell recovery.

A Effect of 48 h treatment with VCR on RPMI8402 cells upon β-catenin knockdown when compared with control cells. Significant differences: $****P \leq 0.0001$, $***P \leq 0.001$, $**P \leq 0.01$.

B Effect of 48 h single and combined treatments with VCR and ICG-001 on RPMI8402 cells viability. Significant differences: $****P \leq 0.0001$, $***P \leq 0.001$, $**P \leq 0.01$, $*P \leq 0.05$.

C Effect of VCR 48 h-treatment on cell viability of Jurkat (left) CCRF-CEM (central) or DND41 (right) cells in the presence or absence of increasing doses of the β-catenin inhibitor ICG-001. Dose–response curves represent the logistic fitting and individual points correspond to mean ± SD. $****P \leq 0.0001$, $***P \leq 0.001$, $**P \leq 0.01$, $*P \leq 0.05$ of IC50 comparison with respect to the chemotherapy single treatment.

D (left) Cell viability of RPMI8402 cells treated with MTX and during the recovery time in the presence or absence of ICG-001 after drug wash-out as fold change relative to day 0 ($n = 2$). (right) Cell viability of RPMI-8402 cells transduced with sh β-catenin or sh control treated with MTX and during the recovery time as fold change relative to day 0 ($n = 1$). $**P \leq 0.01$ with respect to control; $\#P \leq 0.05$ with respect to VCR-treated cells. †† $P \leq 0.01$, † $P \leq 0.05$ with respect to recovery in complete medium.

E Percentage of viability of Jurkat (left), CCRF-CEM (central) and DND41 (right) cells after drug wash-out during recovery in the presence or absence of ICG-001 10 μM at the indicated time points. Significant differences: $****P \leq 0.0001$, $***P \leq 0.001$, $**P \leq 0.01$, $*P \leq 0.05$ with respect to recovery in complete medium.

Data information: For all applicable figure panels, data represent mean ± SD of three independent experiments, except in (D) (D left $n = 2$ independent experiments, and D right $n = 1$). Statistical significance was determined by one-way ANOVA with Tukey's correction for multiple comparison testing for (A and B) or by two-sided Student's *t*-test for (C–E).
Source data are available online for this figure.

 © 2023 The Authors

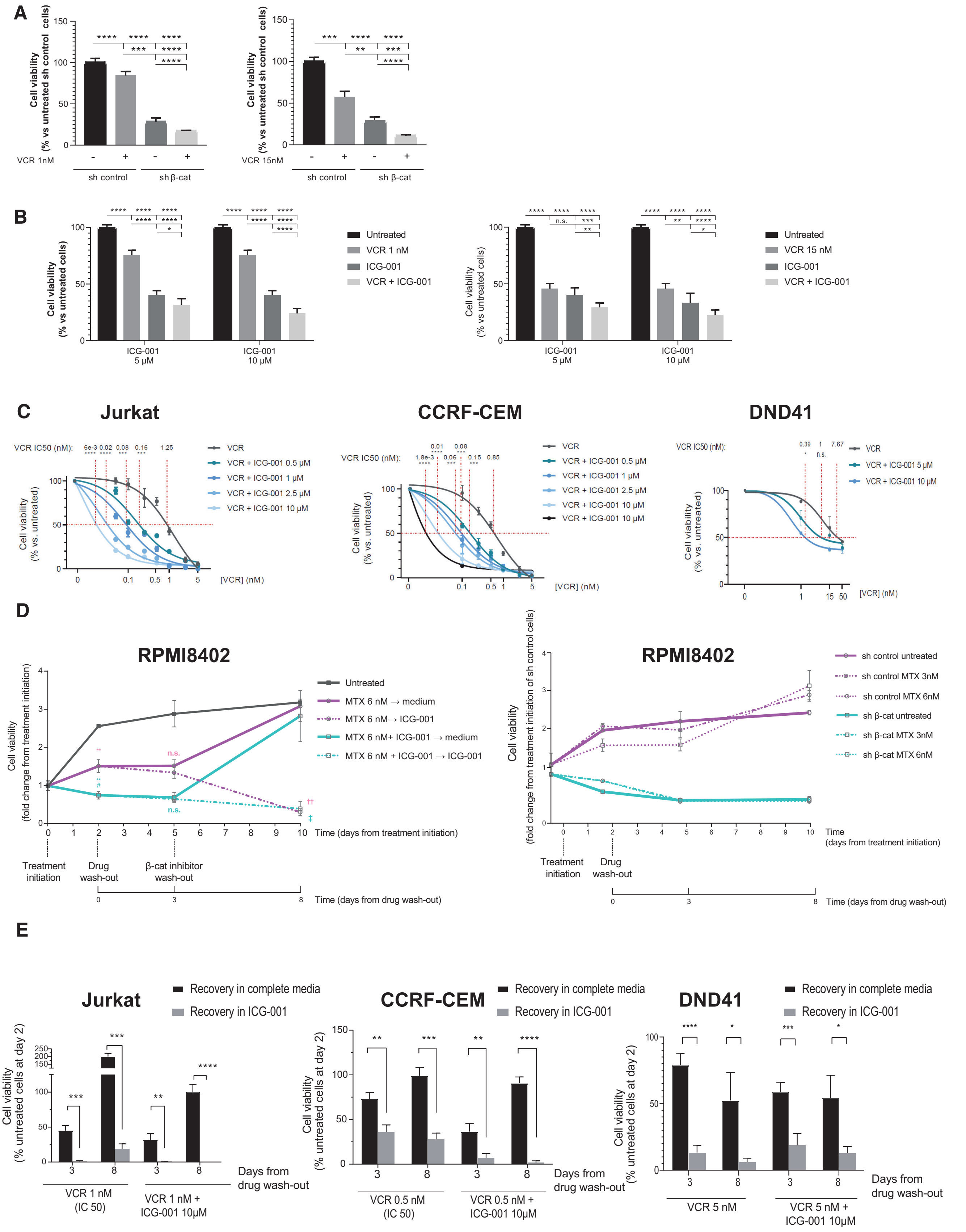

A
Cell viability (% vs untreated sh control cells)
VCR 1nM
sh control
sh β-cat
VCR 15nM
B
Cell viability (% vs untreated cells)
ICG-001 5 μM
ICG-001 10 μM
Untreated
VCR 1 nM
ICG-001
VCR + ICG-001
VCR 15 nM
C
Jurkat
CCRF-CEM
DND41
VCR IC50 (nM):
Cell viability (% vs. untreated)
[VCR] (nM)
VCR
VCR + ICG-001 0.5 μM
VCR + ICG-001 1 μM
VCR + ICG-001 2.5 μM
VCR + ICG-001 10 μM
VCR + ICG-001 5 μM
D
RPMI8402
Cell viability (fold change from treatment initiation)
Untreated
MTX 6 nM → medium
MTX 6 nM→ ICG-001
MTX 6 nM+ ICG-001 → medium
MTX 6 nM + ICG-001 → ICG-001
Time (days from treatment initiation)
Treatment initiation
Drug wash-out
β-cat inhibitor wash-out
Time (days from drug wash-out)
Cell viability (fold change from treatment initiation of sh control cells)
sh control untreated
sh control MTX 3nM
sh control MTX 6nM
sh β-cat untreated
sh β-cat MTX 3nM
sh β-cat MTX 6nM
E
Recovery in complete media
Recovery in ICG-001
Cell viability (% untreated cells at day 2)
Days from drug wash-out
VCR 1 nM (IC 50)
VCR 1 nM + ICG-001 10μM
VCR 0.5 nM (IC 50)
VCR 0.5 nM + ICG-001 10μM
VCR 5 nM
VCR 5 nM + ICG-001 10μM

Figure EV5.
