# Supplementary material for: β‐Catenin activity induces an RNA biosynthesis program promoting therapy resistance in T‐cell acute lymphoblastic leukemia
Source: EMBO Mol Med. 2023 Jan 4;15(2):e16554. doi: 10.15252/emmm.202216554 (PMC9906382; doi:10.15252/emmm.202216554)
Supplement: Supplementary file 8 — PDF+ [file EMMM-15-e16554-s009.pdf]

Article

SOURCE DATA | TRANSPARENT PROCESS | OPEN ACCESS

EMBO Molecular Medicine

β-Catenin activity induces an RNA biosynthesis program promoting therapy resistance in T-cell acute lymphoblastic leukemia

Violeta García-Hernández[1,†], David Arambilet[1,†], Yolanda Guillén[1,†], Teresa Lobo-Jarne[1], María Maqueda[1], Christos Gekas[1], Jessica González[1], Arnau Iglesias[1], Nerea Vega-García[2,3], Inés Sentís[4], Juan L Trincado[4], Ian Márquez-López[1], Holger Heyn[4,5], Mireia Camós[2,3], Lluis Espinosa[1,‡] & Anna Bigas[1,6,*,‡]

Abstract

Understanding the molecular mechanisms that contribute to the appearance of chemotherapy resistant cell populations is necessary to improve cancer treatment. We have now investigated the role of β-catenin/CTNNB1 in the evolution of T-cell Acute Lymphoblastic Leukemia (T-ALL) patients and its involvement in therapy resistance. We have identified a specific gene signature that is directly regulated by β-catenin, TCF/LEF factors and ZBTB33/Kaiso in T-ALL cell lines, which is highly and significantly represented in five out of six refractory patients from a cohort of 40 children with T-ALL. By subsequent refinement of this gene signature, we found that a subset of β-catenin target genes involved with RNA-processing function are sufficient to segregate T-ALL refractory patients in three independent cohorts. We demonstrate the implication of β-catenin in RNA and protein synthesis in T-ALL and provide *in vitro* and *in vivo* experimental evidence that β-catenin is crucial for the cellular response to chemotherapy, mainly in the cellular recovery phase after treatment. We propose that combination treatments involving chemotherapy plus β-catenin inhibitors will enhance chemotherapy response and prevent disease relapse in T-ALL patients.

Keywords chemotherapy resistance; Kaiso; RNA processing; T-ALL; β-catenin
Subject Categories Cancer; Haematology
DOI 10.15252/emmm.202216554 | Received 7 July 2022 | Revised 9 December 2022 | Accepted 13 December 2022 | Published online 4 January 2023
EMBO Mol Med (2023) 15: e16554

Introduction

T-cell Acute Lymphoblastic Leukemia (T-ALL) is an aggressive hematological disease characterized by the outgrowth of cells of the T-lymphoid lineage. T-ALL is a rare disease with an average incidence of 0.6 in 100,000 per year. Children are mostly affected but combinations of chemotherapeutic (CT) drugs at high doses have considerably improved the outcome of these patients from around 50% relapse in 1996 to the current less than 20%. Adult patients have nowadays the worst prognosis (close to 50% survival) and relapse patients from both groups have very dismal prognosis (Bigas *et al*, 2022).

Multiple studies from classical cytogenetics to recent deep genome sequencing of T-ALL cells reveal genetic alterations in crucial transcription factors and cell cycle regulators. Recurrent deletions in CDKN2A/B or PTEN, chromosomal translocations in TAL1/2 or TLX/HOX genes and genetic mutations in RAS, TP53 or PI3K among others have been identified. Activating NOTCH1 mutations are the most recurrent in T-ALL, with about 90% of patients carrying alterations in the pathway. Although NOTCH1 is a clear oncogenic driver of this disease, its role in the initiation and progression of T-ALL is not completely understood and the contribution of additional pathways is under extensive investigation (Tzoneva & Ferrando, 2012).

We previously found that NOTCH1-dependent leukemia requires β-catenin activity for the initial stage of leukemic transformation, and demonstrated a direct correlation between β-catenin deficiency and reduced number of leukemia initiating cells (LIC; Gekas *et al*, 2016), which is in agreement with other reports (Kaveri *et al*, 2013; Dose *et al*, 2014; Giambra *et al*, 2015). However,

1 Program in Cancer Research, Institut Hospital del Mar d'Investigacions Mèdiques (IMIM), CIBERONC, Barcelona, Spain
2 Hematology Laboratory, Hospital Sant Joan de Déu Barcelona, Barcelona, Spain
3 Developmental Tumor Biology Group, Leukemia and Other Pediatric Hemopathies, Institut de Recerca Sant Joan de Déu, CIBERER, Barcelona, Spain
4 CNAG-CRG, Centre for Genomic Regulation (CRG), Barcelona Institute of Science and Technology (BIST), Barcelona, Spain
5 Universitat Pompeu Fabra (UPF), Barcelona, Spain
6 Josep Carreras Leukemia Research Institute (IJC), Barcelona, Spain
*Corresponding author. Tel: +34 933160440; E-mail: abigas@imim.es
†These authors contributed equally to this work as first authors
‡These authors contributed equally to this work as senior authors

© 2023 The Authors. Published under the terms of the CC BY 4.0 license.

β-catenin contribution in T-ALL patients and its functional relevance in the disease is still unclear. Deregulation of Wnt signals have previously been detected in primary T-ALL patients (Ng *et al*, 2014) and analysis of public datasets identified several Wnt/β-catenin pathway elements that are differentially expressed among different T-ALL subtypes at diagnosis (Bigas *et al*, 2020). In addition, alterations in β-catenin cofactors TCF1 and LEF1 have been linked to different T cell malignancies (Yu *et al*, 2012). The mechanistic insights on how TCF/LEF impacts on T-ALL and their dependence on β-catenin are still under debate.

Besides its involvement in supporting LIC activity, β-catenin can play a role in chemotherapy and radiotherapy resistance by regulating expression of DNA damage repair genes (Roy *et al*, 2018). Thus, cells displaying high β-catenin activity may have higher capacity for DNA repair to respond to stress situations, which would result in a survival advantage. In addition, β-catenin through transcriptional activation of MYC (van de Wetering *et al*, 2002; Gekas *et al*, 2016) promotes RNA biogenesis programs and regulates RNA translation.

To further understand the molecular function and clinical impact of β-catenin in primary and relapse T-ALL, we investigated the transcriptional programs that depend on β-catenin in human T-ALL cell lines. We identified a β-catenin-dependent signature that is specifically associated with initial induction failure in T-ALL patients. Importantly, the minimal informative β-catenin dependent signature is enriched in genes related with RNA processing functions. Our work identifies β-catenin as a direct regulator of RNA processing in T-ALL and demonstrates its implication in the eradication of leukemic cells in the recovery phase after chemotherapy.

Results

β-Catenin is recruited to the promoter regions of RNA processing-related factors in T-ALL cells

β-Catenin (CTNNB1) is needed for NOTCH1-induced transformation of T-ALL cells, but the mechanisms behind this requirement are not yet characterized. We here investigated the DNA-binding activity of β-catenin in two T-ALL cell lines (RPMI8402 (pre-T-ALL) and Jurkat (cortical T-ALL relapse)) with high basal levels of nuclear active β-catenin protein that is increased in response to GSK3β inhibition by 25 mM LiCl (Fig EV1A and B). Chromatin immunoprecipitation (ChIP) conditions were established using two different antibodies (R&D and sc-7199) in the presence of the protein–protein crosslink DSG (Fig EV1C). By genome-wide ChIP with β-catenin antibodies, followed by deep sequencing (ChIPseq) in basal and LiCl-treated RPMI8402 cells, we identified 522 genes distributed in 433 peaks (statistically significant and supported by at least two ChIPseq replicates) that were further validated in Jurkat cells (Fig 1A and Dataset EV1). Most peaks overlapped in basal and LiCl conditions (Fig EV1D) and were distributed in promoter regions at < 1 kb distance of the TSS suggesting a role for β-catenin in their transcriptional regulation (Figs 1B and EV1E). Gene enrichment functional analysis of β-catenin targets revealed RNA processing, splicing and ribosomal biogenesis as the most significantly overrepresented functions (FDR adjusted *P*-value < 0.05; Fig EV1F and Dataset EV4). We confirmed β-catenin binding to randomly-selected genes associated with these functions by ChIP-PCR (Fig EV1G).

Motif enrichment analysis of β-catenin DNA-binding regions uncovered ZBTB33/Kaiso as the most represented motif (Fig 1C and Dataset EV1). We also detected enrichment of the Irf6 motif, as well as the previously described SPI1 motif (Zhu *et al*, 2018). However, the canonical TCF/LEF motif was not enriched in any β-catenin ChIP despite detecting high TCF1 and LEF1 protein isoform levels in T-ALL cell lines (Fig EV1A). Since binding of TCF1 to the TCTCGCGAGA (associated with ZBTB33/Kaiso) has been previously reported in ES cells (De Jaime-Soguero *et al*, 2017), we tested whether TCF1, LEF1 or Kaiso were recruited to β-catenin target genes in T-ALL cells. By ChIPseq analysis, we found that the majority of β-catenin targets were also bound by TCF1 and/or LEF1 (95.8%) and about 50% were bound by ZBTB33/Kaiso (Figs 1D–F and EV1H, and Dataset EV1). We next engineered RPMI8402 cells to test the requirement of TCF1, LEF1 and ZBTB33/Kaiso for β-catenin binding to the promoters. Deletion of TCF1 and LEF1 with guides targeting the β-catenin-binding domain of both factors did not affect the short isoforms of TCF1 and LEF1(Xu *et al*, 2017; sTCF1 and sLEF1; Fig EV1I, right) but abrogated or strongly reduced the binding of β-catenin to randomly selected targets by ChIP-PCR analysis

Figure 1. β-catenin chromatin binding in T-ALL cell lines.

A Representative density plot (upper panels) and heatmaps (bottom panels) showing β-catenin chromatin binding centered at TSS ± 10 Kb in RPMI8402 or Jurkat cells after precipitation in the indicated conditions with two different antibodies (R&D or SC). The order in the *y*-axis represents the closest gene annotated to the β-catenin peak obtained in the basal condition with R&D antibody and confirmed in at least another condition (for the RPMI8402 cell line: $n = 5$ for R&D antibody in basal conditions, $n = 2$ for R&D antibody upon LiCl treatment, $n = 2$ for SC antibody in basal conditions, $n = 2$ for SC antibody upon LiCl treatment. For Jurkat cell line: $n = 3$ for R&D antibody in basal conditions in the Jurkat cell line).

B Genomic distribution of β-catenin ChIPseq peaks shown in (A).

C DNA motifs enriched in the β-catenin-binding regions using as an input the peaks obtained in the basal condition with R&D antibody and confirmed in at least another condition.

D Representative heatmap showing the enrichment of 10 Kb upstream and downstream of the identified β-catenin peaks obtained by precipitating TCF1 ($n = 3$), LEF1 ($n = 3$) and Kaiso ($n = 3$; from left to right).

E Venn diagram (left panel) representing the number of β-catenin, TCF1/LEF1 and Kaiso targets obtained from ChIPSeq. Each gene dataset is formed by the genes obtained in at least two different replicates for TCF1, LEF1 and Kaiso. Summary of number of peaks and their corresponding annotated genes (right panel) obtained from ChIPSeq of β-catenin, TCF1, LEF1 and Kaiso.

F Integrative genomic viewer snapshot showing the position of β-catenin, TCF1, LEF1 and Kaiso peaks near the TSS of the indicated genes.

G, H Representative β-catenin ChIP-qPCR of indicated target genes in wt and TCF1/LEF1 long isoform double KO (DLKO) clones (G) and Kaiso KO clones (H) in RPMI8402 cells. Enrichment of each ChIP is normalized to its respective negative IgG control, indicated by a red-dashed line. DLKO1, DLKO2, KKO1 and KKO2 designate independent single-cell derived clones. Graph represents the mean and SD of three technical replicates.

Source data are available online for this figure.

 © 2023 The Authors

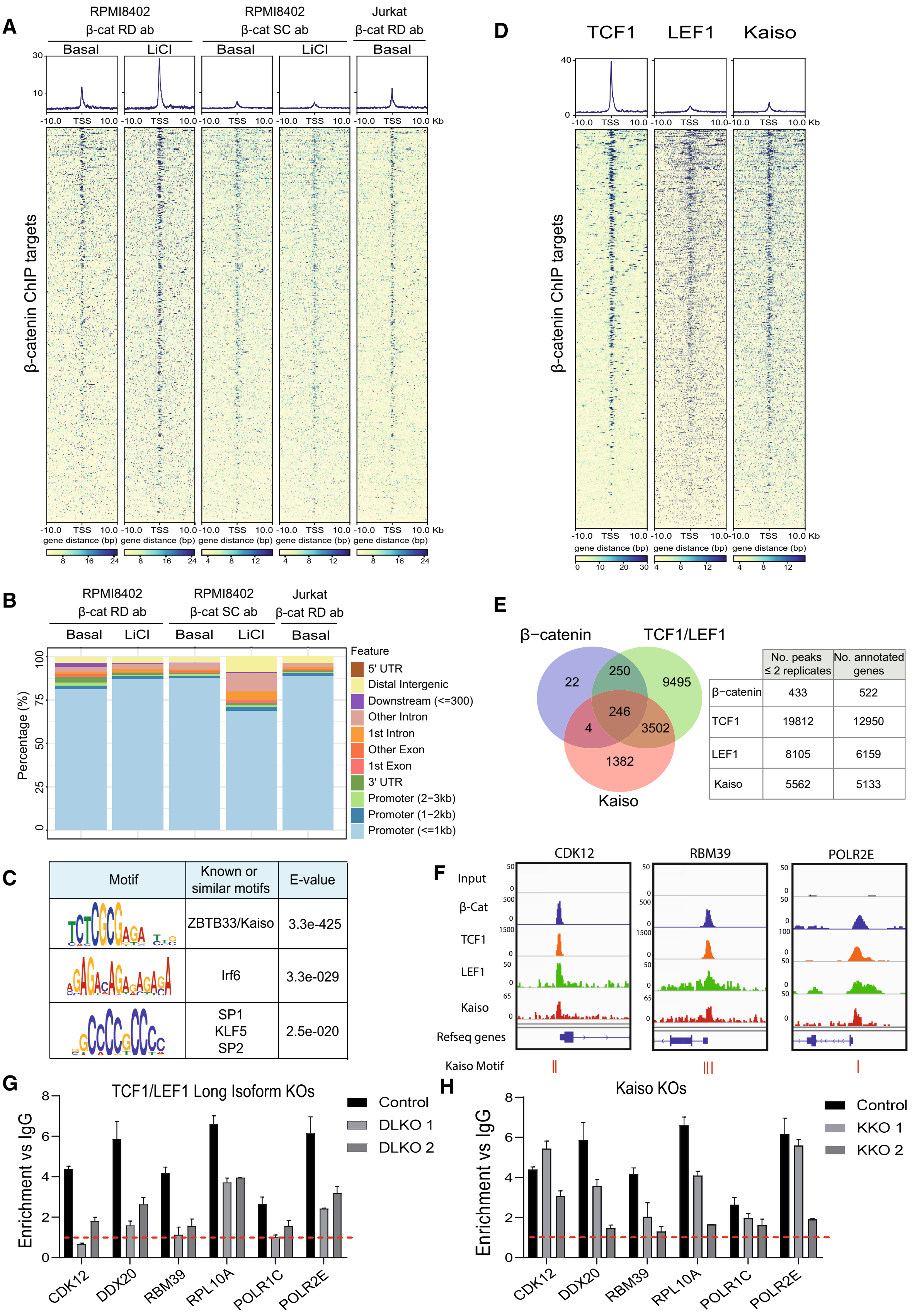
A
RPMI8402
β-cat RD ab
RPMI8402
β-cat SC ab
Jurkat
β-cat RD ab
Basal
LiCl
Basal
LiCl
Basal
β-catenin ChIP targets
-10.0 TSS 10.0 Kb
gene distance (bp)
D
TCF1
LEF1
Kaiso
β-catenin ChIP targets
B
Percentage (%)
Feature
5' UTR
Distal Intergenic
Downstream (<=300)
Other Intron
1st Intron
Other Exon
1st Exon
3' UTR
Promoter (2−3kb)
Promoter (1−2kb)
Promoter (<=1kb)
E
β−catenin
TCF1/LEF1
Kaiso
22
250
9495
246
4
3502
1382
No. peaks ≤ 2 replicates
No. annotated genes
β−catenin 433 522
TCF1 19812 12950
LEF1 8105 6159
Kaiso 5562 5133
C
Motif
Known or similar motifs
E-value
ZBTB33/Kaiso 3.3e-425
Irf6 3.3e-029
SP1 KLF5 SP2 2.5e-020
F
CDK12
RBM39
POLR2E
Input
β-Cat
TCF1
LEF1
Kaiso
Refseq genes
Kaiso Motif
G
TCF1/LEF1 Long Isoform KOs
Enrichment vs IgG
Control
DLKO 1
DLKO 2
CDK12
DDX20
RBM39
RPL10A
POLR1C
POLR2E
H
Kaiso KOs
Enrichment vs IgG
Control
KKO 1
KKO 2
CDK12
DDX20
RBM39
RPL10A
POLR1C
POLR2E

Figure 1.

(Fig 1G). In a lesser extent, deletion of ZBTB33/Kaiso (Fig EV1I, left) resulted in a reduction of β-catenin recruitment to the chromatin of these promoters (Fig 1H). Altogether these results suggested that β-catenin is recruited to specific gene promoters (i.e. genes related with RNA processing) together with TCF1/LEF1 and/or ZBTB33/Kaiso to regulate gene transcription in T-ALL cells.

β-Catenin regulates expression of RNA-processing and mitotic-related genes in an opposite manner

We studied the chromatin landscape of β-catenin target genes in T-ALL cells. By ChIPseq analysis, we found that β-catenin target promoters were preferentially enriched for activation histone marks such as total acetylated Histone3 (H3Ac) or H3K27Ac and H3K4me3 compared with repressive H3K27me3 marks in different T-ALL cell lines (Fig EV2A), which suggested a general active transcriptional status. To better understand the functional impact of β-catenin on its target genes, we investigated the transcriptional effects of knocking down β-catenin in RPMI8402 cells. We compared the transcriptome of cells transduced with sh-control and sh-β-catenin by RNAseq analysis ($n = 3$ per experimental condition) and found 4,929 differentially expressed genes (DEGs) between both conditions (FDR adjusted P-value < 0.05; Fig 2A and B and Dataset EV2). Functional enrichment analysis indicated that upregulated genes were enriched in mitotic related functions, while downregulated genes were enriched in RNA and protein processing functions (Fig 2C and Dataset EV4). Next, we crossed RNAseq and ChIPseq data to uncover β-catenin-target genes that were deregulated after depletion of β-catenin. We identified 79 β-catenin-target genes that were downregulated and 77 that were upregulated upon β-catenin knockdown (Fig 2D and Dataset EV2). We considered the downregulated genes as bona fide β-catenin targets and selected some of these genes to confirm the transcriptional activation dependence of β-catenin in sh-β-catenin cells (Fig 2E) or by treating cells with two different β-catenin inhibitors FH535 (blocking interaction of β-catenin and PPRϒ; Handeli & Simon, 2008) and ICG-001 (Gang *et al*, 2014; blocking interaction with CBP; Figs 2F, and EV2B and C). In both conditions, we found a consistent reduction of gene expression.

Since ZBTB33/Kaiso was also recruited to a subset of β-catenin-targets (Fig 1D–F) and has been previously found to repress β-catenin-dependent transcription (Park *et al*, 2005), we tested whether it was involved in the repression of a subset of β-catenin targets. Unexpectedly, knocking down ZBTB33/Kaiso by specific sh-RNA in RPMI8402 resulted in a significant reduction of the selected β-catenin-target gene expression (Fig 2G), suggesting that ZBTB33/Kaiso was involved in transcriptional activation rather than inhibition of β-catenin-target genes.

The most enriched functions in β-catenin-bound and transcriptionally regulated genes were related to RNA processing and protein synthesis (Figs EV1F and 2C, and Dataset EV4). Thus, we next investigated the possible role of β-catenin in the regulation of RNA synthesis *in vitro*. We measured 5-Ethynyl Uridine (EU; conjugated to Alexa Fluor 488) incorporation to nascent RNA in β-catenin-depleted or inhibited RPMI8402 cells. We found that both sh-β-catenin and treatment with the β-catenin inhibitors ICG-001 or FH535 reduced the total RNA transcription compared with their controls (Fig 2H). Similarly, we determined protein synthesis in basal, sh-β-catenin or IGC-001 and FH535-treated RPMI8402 cells by measuring the incorporation of O-propargyl-puromycin (OPP) to newly translated proteins by flow cytometry analysis. As internal control, we confirmed protein synthesis blockage in cycloheximide (CHX)-treated control cells (Fig 2I). Knockdown of β-catenin and inhibition with either ICG-001 or FH535 significantly decreased OPP incorporation (Fig 2I) further indicating that functional β-catenin depletion reduced protein synthesis in T-ALL cells. We cannot exclude that other β-catenin targets such as MYC also converge with β-catenin in the regulation of RNA and protein synthesis as previously described (Barna *et al*, 2008).

Since genes statistically up-regulated after β-catenin knockdown are associated with mitotic processes, we performed cell cycle analysis of RPMI8402 cells to test the effect of β-catenin inhibition (Fig EV2D and E). Consistent with previous studies (Tetsu & McCormick, 1999), we found that cell cycle progression is repressed in β-catenin-inhibited cells (Fig EV2D and E). Thus, enrichment of cell cycle related categories refers exclusively to mitotic organization, but does not result in cell cycle progression.

Figure 2. β-catenin-regulated transcriptional program in T-ALL cell lines.

A MA plot showing the log2 fold changes for each gene over the mean of normalized counts obtained in the RNAseq analysis of sh-β-catenin vs. sh-control ($n = 3$ for each condition). Dots in blue are those genes differentially expressed with an adjusted P-value < 0.05.

B Heatmap showing the differentially expressed genes (adjusted P-value < 0.05) for each condition.

C Biological functions significantly enriched (adjusted P-value < 0.05) in groups of genes downregulated (Down) or upregulated (Up) in the RNAseq analysis.

D Venn diagram with differentially expressed genes downregulated (DEGs Down) or upregulated (DEGs UP) in the RNAseq analysis compared with the genes obtained in the β-catenin ChIPseq analysis obtained in the basal condition with R&D antibody and confirmed in at least another condition (ChIP targets).

E Relative mRNA expression of β-catenin target genes in the RPMI8402 cell line (right panel) after knockdown of β-catenin (left panel) compared with control. Graph represents the mean and SD of six independent experiments.

F Relative mRNA expression of β-catenin target genes in the RPMI8402 cell line after treatment with the β-catenin inhibitor ICG-001 (10 μM, overnight). Graph represents the mean and SD of three independent experiments.

G Relative mRNA expression of β-catenin target genes in the RPMI8402 cell lines (right panel) after knockdown of ZBTB33/Kaiso (left panel) compared with control. Graph represents the mean and SD of three independent experiments.

H, I Representative flow cytometry histograms of EU incorporation into RNA (H) or OPP incorporation into nascent peptides (I) in the indicated conditions (upper panels). Quantification of relative EU or OPP incorporation. Graph represents the mean and SD of three independent experiments (bottom panels). Cells were pretreated with FH353 30 μM or ICG-001 10 μM (or DMSO) overnight prior to EU or OPP incubation.

Data information: Statistical significance was determined by two-sided Student's t-test.
Source data are available online for this figure.

© 2023 The Authors

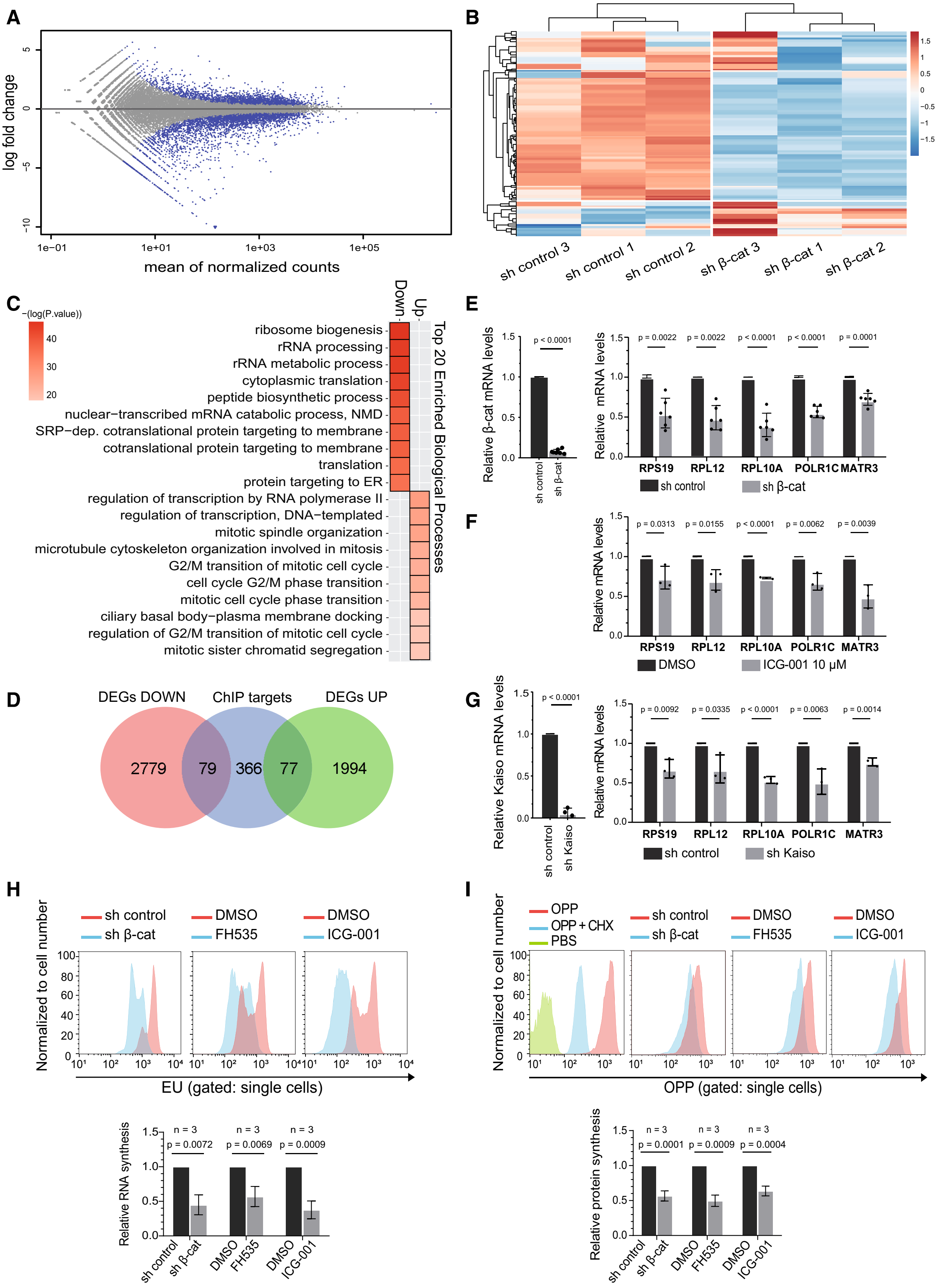
A
log fold change
mean of normalized counts
B
sh control 3
sh control 1
sh control 2
sh β-cat 3
sh β-cat 1
sh β-cat 2
C
−(log(P.value))
Down
Up
Top 20 Enriched Biological Processes
ribosome biogenesis
rRNA processing
rRNA metabolic process
cytoplasmic translation
peptide biosynthetic process
nuclear−transcribed mRNA catabolic process, NMD
SRP−dep. cotranslational protein targeting to membrane
cotranslational protein targeting to membrane
translation
protein targeting to ER
regulation of transcription by RNA polymerase II
regulation of transcription, DNA−templated
mitotic spindle organization
microtubule cytoskeleton organization involved in mitosis
G2/M transition of mitotic cell cycle
cell cycle G2/M phase transition
mitotic cell cycle phase transition
ciliary basal body−plasma membrane docking
regulation of G2/M transition of mitotic cell cycle
mitotic sister chromatid segregation
D
DEGs DOWN
ChIP targets
DEGs UP
2779
79
366
77
1994
E
Relative β-cat mRNA levels
p < 0.0001
Relative mRNA levels
RPS19
RPL12
RPL10A
POLR1C
MATR3
sh control
sh β-cat
F
Relative mRNA levels
DMSO
ICG-001 10 μM
G
Relative Kaiso mRNA levels
sh control
sh Kaiso
H
Normalized to cell number
EU (gated: single cells)
Relative RNA synthesis
sh control
sh β-cat
DMSO
FH535
ICG-001
I
OPP
OPP + CHX
PBS
OPP (gated: single cells)
Relative protein synthesis

Figure 2.

β-Catenin transcriptional target gene signature identified T-ALL refractory patients with worst outcome

To test the relevance of the transcriptional programs identified as β-catenin targets in T-ALL, we interrogated the transcriptome of an exploratory cohort of 40 primary samples from children with T-ALL (2–18 years) at diagnosis who either did not respond to the treatment (induction failure/refractory; $N = 6$), failed to achieve complete remission (relapsed; $N = 13$) or remained in complete continuous remission ($N = 21$; COG Study 9404, GSE14618 only samples with available survival data; Winter *et al*, 2007; Asselin *et al*, 2011). First, these samples were grouped using a non-supervised hierarchical model considering the expression pattern of all 156 DEG identified in the β-catenin-depleted RPMI8402 model (79 down- plus 77 up-regulated; see Fig 2D). This analysis generated three clusters of patients (P_A, P_B and P_C; Fig EV3A) with P_A showing the worst disease-free survival (DFS; $P = 0.011$) and accumulation of induction-failure (refractory cases) within the P_A group (five out of six; Fisher exact test, $P = 0.02$; Fig EV3A and B). P_A samples were characterized by high expression of genes related with RNA processing functions and low expression of genes of the DNA replication and mitotic-related functions (Appendix Fig S3A and Dataset EV4). The median expression levels of β-catenin and ZBTB33/Kaiso were slightly higher in the worst prognostic cluster PA than in the other two clusters (PB and PC). However, when considering TCF1 and LEF1, PA cluster showed significantly lower levels than the rest (Fig EV3C). A similar behavior was observed for TCF1 and LEF1 in the refractory patients when compared with relapse and remission samples, while β-catenin and ZBTB33/Kaiso were not different (Fig EV3D). However, a Kaplan–Meier survival curve showed that the outcome of patients with high or low levels of TCF1 (Fig EV3G) or LEF1 (Fig EV3H) was not significantly different. Similar negative results were obtained when considering highest and lowest levels of β-catenin (Fig EV3E) and ZBTB33/Kaiso levels (Fig EV3F), underlying the importance of specific β-catenin transcriptional programs in the initial chemotherapy response.

We then restricted our gene signature to 79 downregulated genes upon β-catenin depletion in RPMI8402 cells, which we considered as direct transcriptional target genes. We applied a new non-supervised hierarchical clustering (Fig 3A) and the patients' dendrogram was "arbitrarily" divided into five clusters (P1–P5) with P1 including five out of the six refractory patients (Fig 3A and B) showing a poor DFS ($P = 0.0039$) and overall survival (OS; $P = 0.025$; Fig 3C). Comparable analysis of the 77 upregulated target genes in the sh-β-catenin cells did not show significant differences in DFS ($P = 0.15$) and OS ($P = 0.32$) among groups (Fig EV4A). Multivariate Cox proportional model demonstrated that the 79 DEG β-catenin signature represent a powerful independent prognosis factor (HR = 18.18; $P = 0.003$) followed by the ETP + ABD phenotype (HR = 7.64; $P = 0.03$; Fig 3E).

The 79 gene β-catenin signature was initially selected based on its downregulation imposed by β-catenin depletion in RPMI8402. However, these genes showed variable expression profiles in the patient samples, mainly representing three modules of genes with coordinated expression (G1, G2 and G3). In the poor prognosis P1 cluster of patients, the G1 module was primarily upregulated and included most of the genes involved in RNA processing functions. In contrast, G2 genes (enriched for mitotic and DNA repair functions) were downregulated and no function was significantly associate to the G3 module (Fig 3D and Dataset EV4).

We then analyzed the set of genes that were differentially expressed between refractory and remission samples ($n = 3,268$, $P < 0.05$, see Dataset EV3). Interestingly, 42 out of 156 β-catenin-dependent genes (as defined in the sh-β-catenin RPMI8402 model) corresponded to genes differentially expressed in refractory samples (25 upregulated including RNA processing-related genes and 17 downregulated; Fig EV4B and Dataset EV3). A Chi Square test with Yates correction showed that the number of β-catenin targets differentially expressed between refractory and remission patients (42/156; 26.92%) was higher than that expected by chance ($P = 0.0055$). These 42 genes were also sufficient to cluster refractory patients in a new unsupervised analysis, as expected (Fig EV4C). These results uncover the presence of a bona-fide β-catenin-dependent signature, which mainly contains genes involved in RNA processing functions, that is associated with therapeutic refraction in T-ALL.

A β-catenin-dependent RNA processing signature predicts response to therapy in T-ALL patients at diagnosis

We next performed single-sample gene set enrichment analysis (ssGSEA; Barbie *et al*, 2009) to evaluate the enrichment prolife of G1 and G2 signature in the individual samples. In the discovery cohort, we found an inversed correlation between G1 and G2 ssGSEA with five out of six refractory patients grouped within the 25% of patients (referred as Q4) with the highest expression of G1 genes (Fig 4A) with a significantly worse OS ($P = 0.036$) and DFS ($P = 0.052$; Fig EV4E). We applied the same strategy to two additional T-ALL cohorts that lack survival time information: 42 patients from the COG Study 8707 (additional GSE14618 samples) and samples from an independent cohort obtained with RNASeq (EGAS00001000536; Fig 4B and C). Interestingly, both cohorts

Figure 3. Bona fide β-catenin target gene expression in T-ALL patients is associated with chemotherapy refraction. ▶

A Non-supervised hierarchical classification of T-ALL patients (GSE14618, samples with available survival data $N = 40$) according to the expression of the 79 β-catenin targets downregulated after β-catenin knockdown. T-ALL phenotype according to cohort information, patient outcome, vital status and age are depicted on the top of the heatmap. Genes are represented on the left side and patients are shown in the upper part. ETP, Early T-cell precursor; ABD, absence of biallelic TCRgamma locus deletion.

B Distribution of refractory, remission and relapse cases in each patients' cluster.

C Kaplan–Meier curves representing disease free (DSF, top panel) and overall survival (OS, bottom panel) probability for groups of patients P1 ($N = 7$), P2 ($N = 5$), P3 ($N = 4$), P4 ($N = 9$) and P5 ($N = 15$) obtained in (A). Time is represented in years. Statistical significance among groups was determined by log-rank test.

D Top 25 Gene Ontology Biological functions significantly enriched (adjusted P-value < 0.05) in groups of genes G1 ($N = 26$), G2 ($N = 35$) and G3 ($N = 12$).

E Forest plot for the multivariate Cox proportional hazard regression model showing hazard ratio estimates and 95% confidence intervals. Statistical significance among levels for each factor was determined by Wald test, *$P < 0.05$; **$P < 0.01$. Only patients with complete data were considered ($N = 34$).

© 2023 The Authors

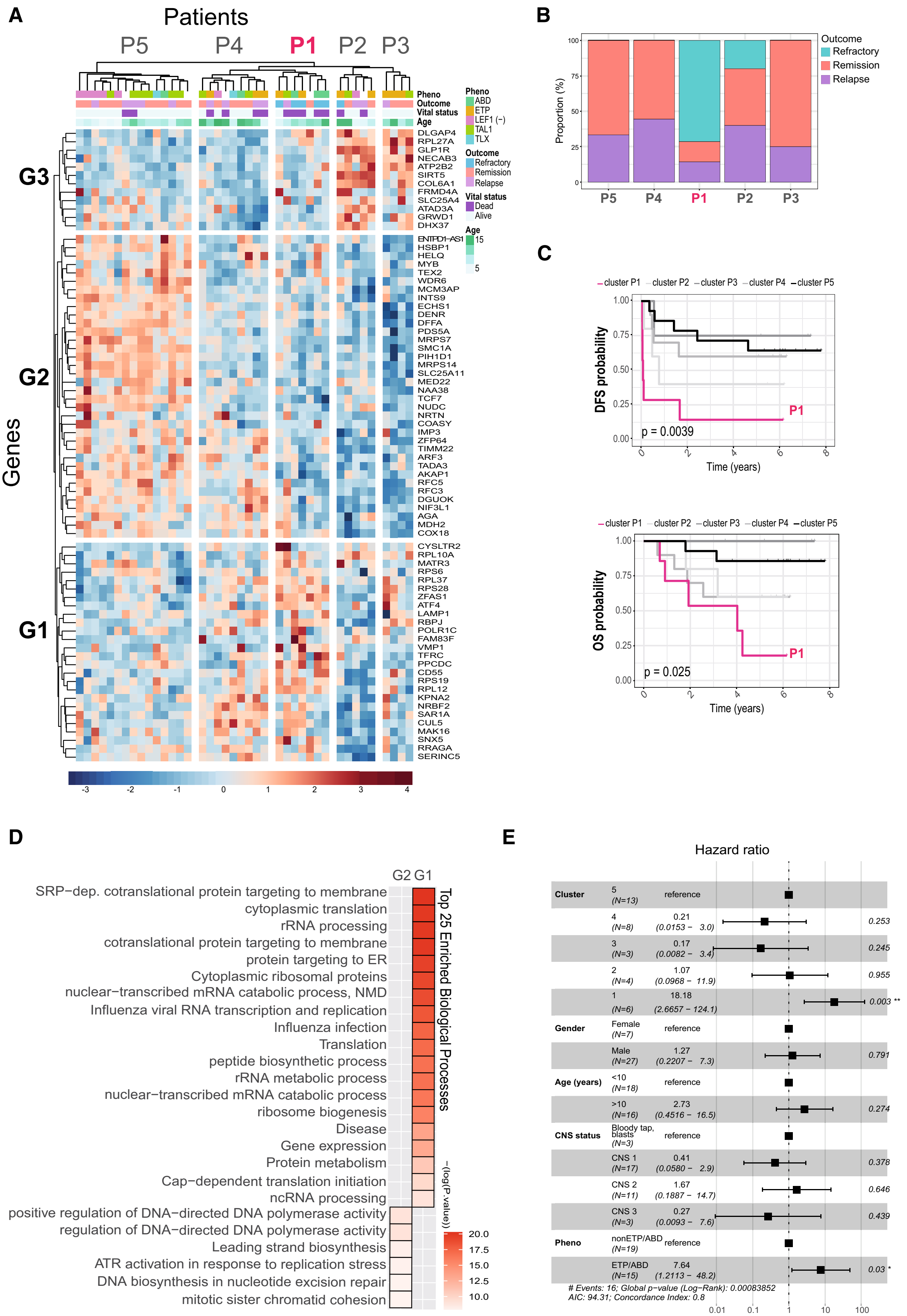
A
Patients
P5 P4 P1 P2 P3
Pheno
Outcome
Vital status
Age
G3
G2
G1
Genes
B
Proportion (%)
Outcome
Refractory
Remission
Relapse
C
DFS probability
p = 0.0039
Time (years)
OS probability
p = 0.025
D
G2 G1
SRP−dep. cotranslational protein targeting to membrane
cytoplasmic translation
rRNA processing
cotranslational protein targeting to membrane
protein targeting to ER
Cytoplasmic ribosomal proteins
nuclear−transcribed mRNA catabolic process, NMD
Influenza viral RNA transcription and replication
Influenza infection
Translation
peptide biosynthetic process
rRNA metabolic process
nuclear−transcribed mRNA catabolic process
ribosome biogenesis
Disease
Gene expression
Protein metabolism
Cap−dependent translation initiation
ncRNA processing
positive regulation of DNA−directed DNA polymerase activity
regulation of DNA−directed DNA polymerase activity
Leading strand biosynthesis
ATR activation in response to replication stress
DNA biosynthesis in nucleotide excision repair
mitotic sister chromatid cohesion
Top 25 Enriched Biological Processes
−(log(P-value))
E
Hazard ratio
Events: 16; Global p-value (Log-Rank): 0.00083852
AIC: 94.31; Concordance Index: 0.8

Figure 3.

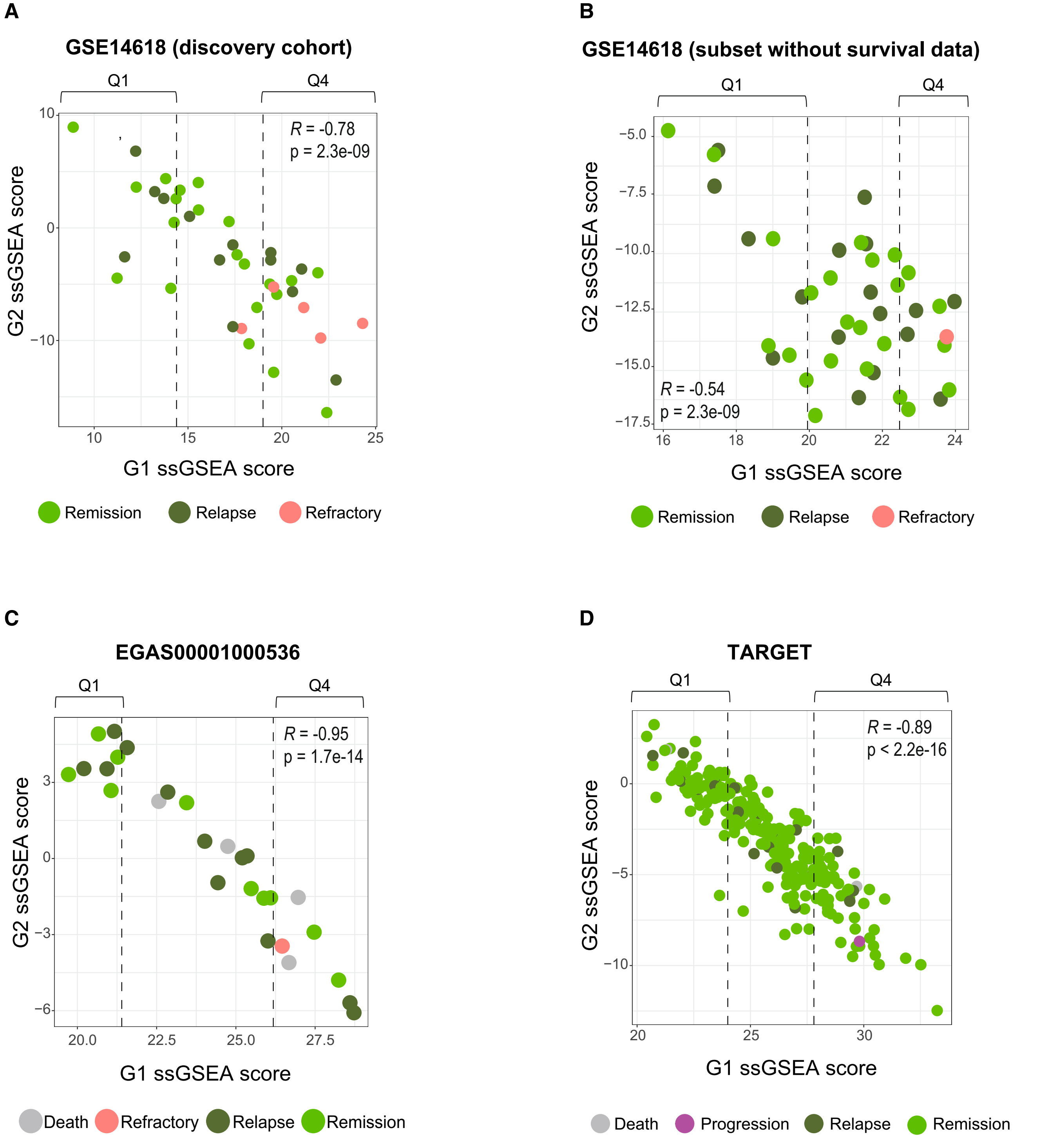

Figure 4. A β-catenin-dependent RNA processing signature predicts response to therapy in T-ALL patients at diagnosis.

A–D Correlation of ssGSEA enrichment for G1 and G2 modules from Fig 3A in patients from the discovery cohort (A), subset of GSE14618 cohort with no survival data available (B), EGA cohort (C) and TARGET cohort (D). Q1 indicates the 25% of the patients with the lowest G1 ssGSEA scores while Q4 represents the 25% of the patients with the highest G1 ssGSEA scores within each cohort. Pearson correlation coefficients are shown.

maintained the inverse correlation between G1 and G2 modules ($R = -0.54$ $P < 0.0001$ and $R = -0.95$ $P < 0.0001$) with refractory samples (one in each cohort) comprised in the 25% of patients with the highest expression of G1 (Fig 4B and C). In all analyses, poor prognosis samples including refractory cases showed the highest levels of β-catenin expression (Fig EV4D).

© 2023 The Authors

Finally, we performed the ssGSEA of a third validation cohort (TARGET study) with 253 primary samples corresponding to children and young adults (97% of patients < 20 years; Dunsmore *et al*, 2020). This cohort contains 230 remission, 20 relapse and a single patient classified as disease progression with marginal response to chemotherapy (31 days of DFS). Consistent with previous analysis, expression levels of genes from G1 and G2 showed a negative correlation ($R = -0.89$ $P < 0.0001$) and the only "refractory" patient was found in the Q4 quartile, with highest expression of G1 (Fig 4D). However, Kaplan–Meier analysis of the patients comprised in Q4 did not show any difference in prognosis (Fig EV4F), further indicating that the β-catenin-signature is only predictive of the chemotherapy response failure.

Inhibition of β-catenin improves response to chemotherapy in T-ALL *in vitro* and *in vivo*

Our data suggests a role for β-catenin dependent transcriptional programs in the outcome of cancer cells after chemotherapy treatments. To evaluate this possibility, we tested the effect of β-catenin on the survival of RPMI8402 treated with different chemotherapy drugs used for the treatment of T-ALL.

First, we treated β-catenin knocked-down RPMI8402 cells with the general chemotherapeutic agent vincristine (VCR) and found that cells with reduced levels of β-catenin were 15 times more sensitive to the treatment than control cells (IC50 from 14.6 to 0.95 nM; Figs 5A and EV5A). Next, we investigated the effect of adding the ICG-001 β-catenin inhibitor to the VCR treatment and observed a dose-dependent reduction of cell viability after 48 h of treatment in the presence of the inhibitor (Figs 5B and EV5B). We also tested the effect of the inhibitor in the treatment with Methotrexate (MTX), L-Asparaginase (L-Asp) and Cytarabine (Ara-C) and similarly found that RPMI8402 cells were more sensitive to the treatment when the β-catenin inhibitor was added (Fig 5C). Similar experiments were performed with other cell lines (Fig EV5C). These results indicated that the levels of β-catenin activity can determine the response of T-ALL cell lines to chemotherapeutic drugs.

To test the effect of β-catenin in the cell recovery after intense chemotherapy treatment, we treated cells with VCR (IC50) alone or with ICG-001 for 2 days and after the drug washout we let the surviving cells recover in the presence or absence of the β-catenin inhibitor (Fig 5D–F). In these conditions, we found that 15–25% of MTT activity remained 8 days after VCR washout, however in the presence of the β-catenin inhibitor we detected less than 5% of MTT activity at that time. Consistent with the results with the ICG-001 inhibitor, cells knocked down for β-catenin could not recover after drug washout (Fig 5G–I), indicating that the levels/activity of β-catenin are important for cells to recover after treatment. VCR treatment in alternative T-ALL cell lines (Fig EV5E) and MTX treatment in RPMI8402 cells (Fig EV5D) show a similar recovery defect in the presence of ICG-001 or sh β-catenin.

To test the efficacy of ICG-001 in chemotherapy response *in vivo*, we transplanted 5×10^5 RPMI8402 T-ALL cells into sublethally irradiated NSG mice and were treated with a chemotherapy cocktail (Vincristine, Dexamethasone and L-Asparaginase) starting 3 days later. The treated animals were divided in two groups receiving DMSO vehicle ("Chemo + DMSO", $n = 4$) or ICG-001 β-catenin inhibitor ("Chemo + ICG-001", $n = 4$), respectively (Fig 6A). Two more mice were left untreated and used as controls to monitor leukemic evolution (Fig 6A). Twenty days after treatment initiation, one control and one "Chemo + DMSO" mice reached a humane endpoint and were sacrificed. We compared the leukemic burden persisting in mice after 3 weeks of treatment initiation (when first animals had to be sacrificed) and found that treatment with β-catenin inhibitor plus chemotherapy imposed a reduction of the leukemic burden in PB (Fig 6B and C) and BM, and strikingly reduced the number of T-ALL cells in the liver and spleen (compared with chemotherapy alone) as detected by flow cytometry (Fig 6C). Moreover, liver visual inspection showed an almost abrogation of tumor nodules (Fig 6D) that was confirmed by H&E staining (Fig 6G) and quantified (Fig 6H). Liver and spleen relative weight was also significantly decreased in the ICG-001-treated animals (Fig 6E and F).

Altogether our results indicate that cells with functional β-catenin have higher chemotherapy resistance and higher capacity to

Figure 5. β-catenin activity determines cell recovery after chemotherapy treatment in T-ALL cells.

A Effect of Vincristine (VCR) treatment on cell viability of RPMI8402 cells transduced with sh β-catenin or sh-control. Dose–response curves represent the logistic fitting and individual points correspond to mean ± SD of three independent experiments. ***$P \leq 0.001$ of IC50 comparison with respect to sh control.

B, C Effect of VCR (B), Methotrexate (MTX) (C left), L-Asparaginase (L-ASP) (C central) or Cytarabine (Ara-C) (C right) treatment in the presence or absence of increasing doses of the β-catenin inhibitor ICG-001. Dose–response curves represent the logistic fitting and individual points correspond to mean ± SD of three independent experiments. ****$P \leq 0.0001$, ***$P \leq 0.001$, **$P \leq 0.01$, *$P \leq 0.05$ of IC50 comparison with respect VCR-treated cells.

D Schematic diagram of the experimental approach used for the drug recovery experiments in (E) and (F). Figure partially created with Biorender.com.

E Cell viability of RPMI8402 cells as fold change relative to day 0 ($n = 3$). VCR, 15 nM; ICG-001, 10 μM. ****$P \leq 0.0001$ with respect to control; ###$P \leq 0.001$ with respect to VCR-treated cells.

F Percentage of viability of RPMI8402 cells after drug wash-out during recovery in the presence or absence of ICG-001 10 μM at the indicated time points. ****$P \leq 0.0001$, ***$P \leq 0.001$, **$P \leq 0.01$, *$P \leq 0.05$ with respect to recovery in complete medium.

G Schematic diagram of the experimental approach used for the drug recovery experiments in (H) and (I). Figure partially created with Biorender.com.

H Cell viability of RPMI8402 cells transduced with sh β-catenin or sh-control as fold change relative to day 0 ($n = 3$). ****$P \leq 0.0001$ untreated sh β-catenin with respect to untreated sh control; ####$P \leq 0.0001$, VCR 1 nM sh β-catenin with respect to VCR 1 nM sh control; ++++$P \leq 0.0001$, VCR15nM sh β-catenin with respect to VCR 15 nM sh control.

I Percentage of viability of RPMI8402 cells upon β-catenin knockdown after drug wash-out when compared with control at the indicated time points. ****$P \leq 0.0001$, ***$P \leq 0.001$, **$P \leq 0.01$, with respect to sh control.

Data information: For all applicable figure panels, data represent mean ± SD of three independent experiments. Statistical significance was determined by two-sided Student's *t*-test for (A, B, C, F, I, and H) (at day 0), or by one-way ANOVA with Tukey's correction for multiple comparison testing for (E and H) (at day 2). Exact *P*-values are disclosed in the Source data file.
Source data are available online for this figure.

© 2023 The Authors

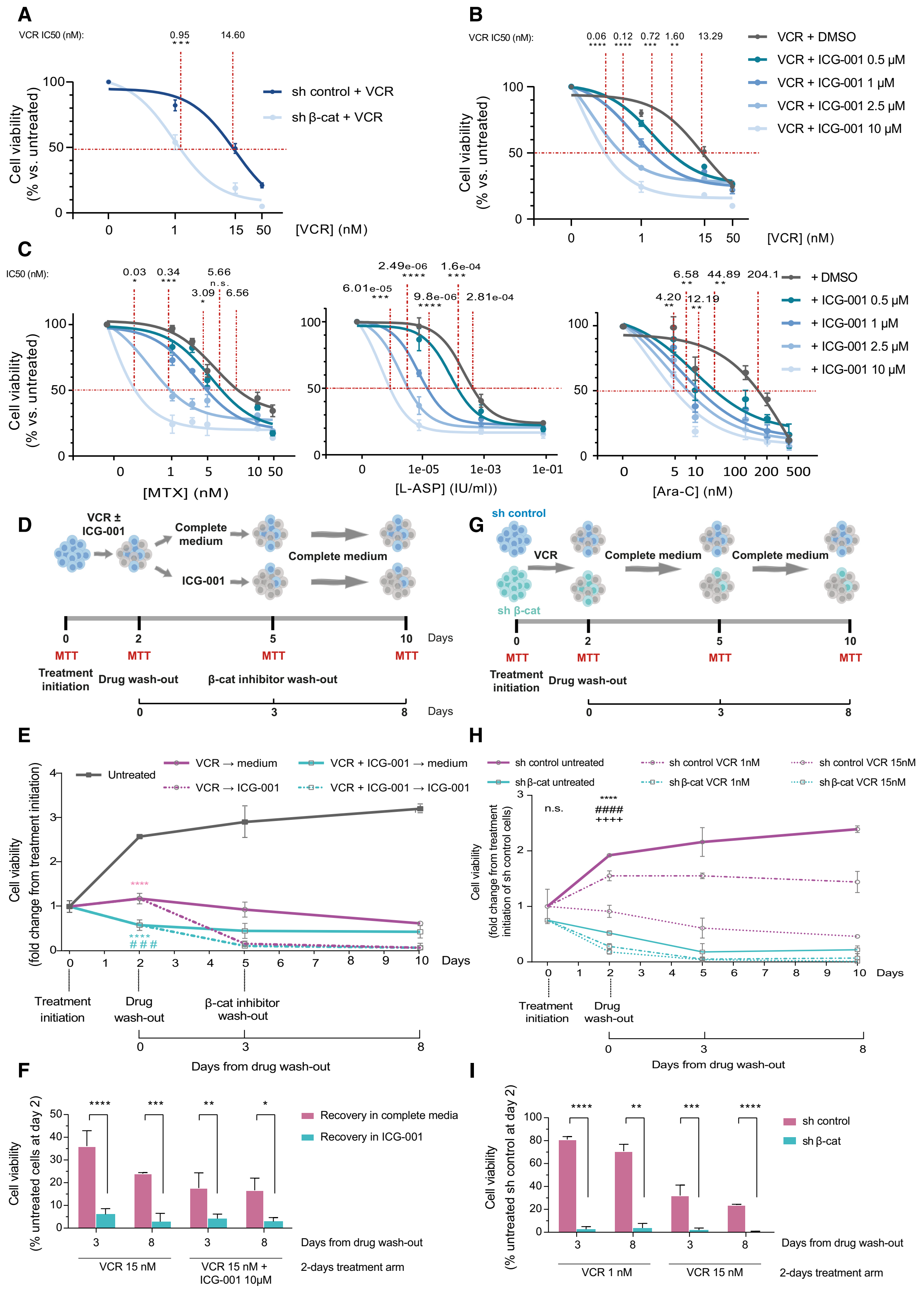

Figure 5.

 © 2023 The Authors

recover after chemotherapy treatment. We propose that combining β-catenin inhibitors with or after chemotherapy administration may reduce the risk of leukemic relapse.

Discussion

Multiple studies identified β-catenin as an essential cancer driver and relevant at different stages of leukemogenesis and leukemic stem cell programs. We now find that β-catenin transcriptional activity in T-ALL regulates a specific signature enriched in RNA processing functions that identifies patients that failed to respond to current therapy regimes. T-ALL cell lines with active β-catenin are more capable to survive after chemotherapy treatments thus opening the possibility for clinical application. Wnt/β-catenin had been previously associated with drug-resistance in different leukemia models (Zhou *et al*, 2017; Carter *et al*, 2019; Perry *et al*, 2020) and solid tumors (Bugter *et al*, 2021; Kaur *et al*, 2021); we now show the importance of β-catenin activity in cell recovery after chemotherapy and patient response to treatment.

Outcome of T-ALL pediatric patients was poor before the use of high-dose chemotherapy treatments. Current intensive treatments

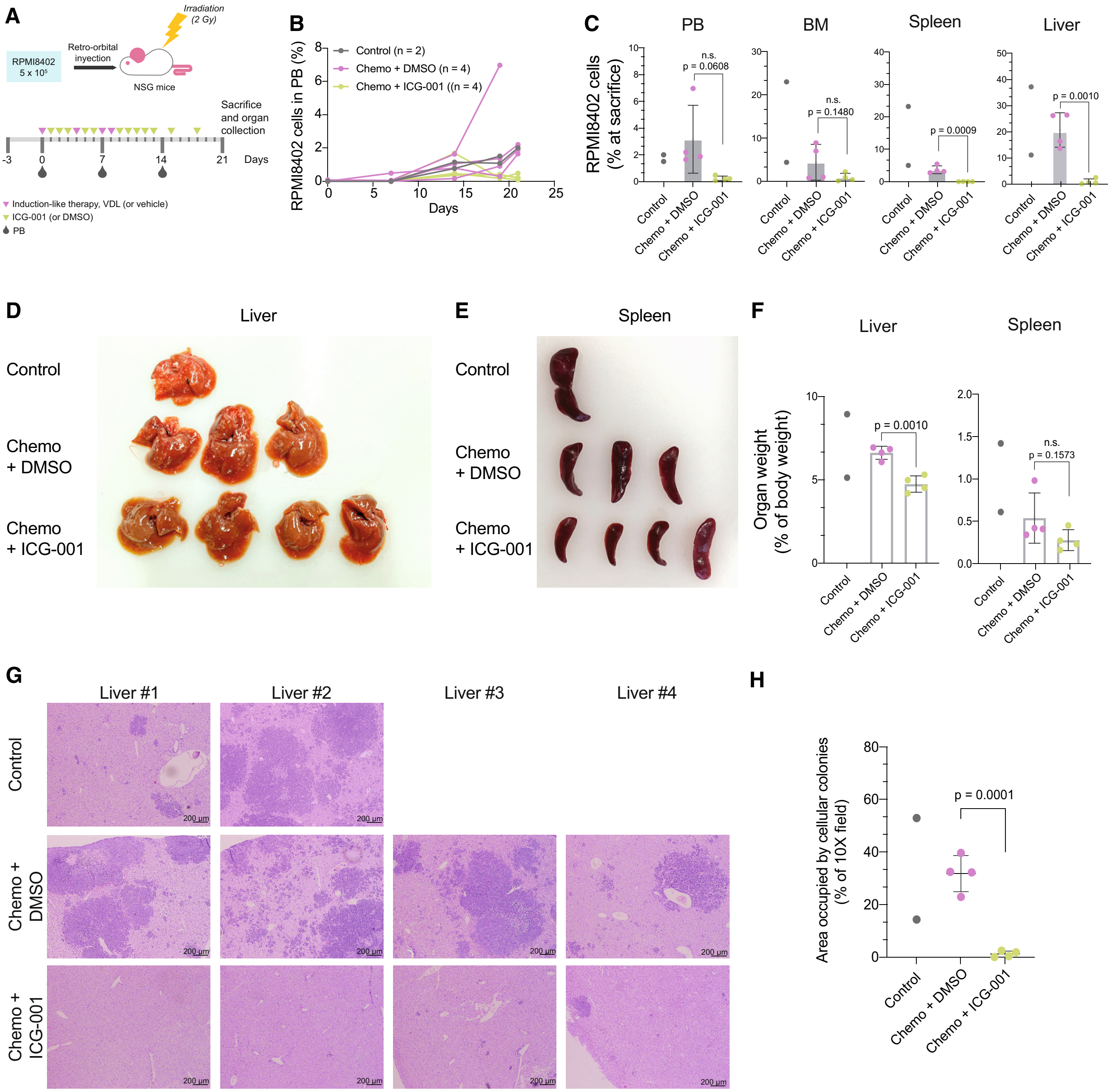

Figure 6.

Figure 6. β-catenin inhibition enhances chemotherapy response in a mouse model of T-ALL.

A Experimental design of *in vivo* treatment of NSG mice transplanted with RPMI8402 cells. Induction-like chemotherapy: V (vincristine, 0.15 mg kg^{-1} day^{-1}), D (dexamethasone, 5 mg kg^{-1} day^{-1}), L (L-Asparaginase, 1,000 IU kg^{-1} day^{-1}). ICG-001: 10 mg kg^{-1} day^{-1}.

B Percentage of RPMI8402 cells in PB at the indicated time points monitoring leukemic progression in the mouse model. RPMI8402 cells identified as human $CD45^{+}$HLA-$ABC^{+}CD5^{+}$ by flow cytometry and gated on DAPI negative cells.

C Percentage of RPMI8402 cells in PB, BM, spleen and liver at sacrifice. RPMI8402 cells identified as human $CD45^{+}$HLA-$ABC^{+}CD5^{+}$ by flow cytometry and gated on DAPI negative cells.

D, E Photograph of the livers (D) and spleen (E) collected at sacrifice with the indicated treatments. One control (liver #1 in Fig 6G) and one Chemo + DMSO (liver #4 in Fig 6G) mice were sacrificed 2 days before the end of the experiment following humane endpoint criteria.

F Quantification of spleen (left panel) and liver (right panel) weight normalized to the mouse body weight at sacrifice.

G H&E staining of representative images per each mouse treated as indicated.

H Percentage of liver area covered by cellular colonies per 10× micrograph. Four different images were quantified per animal and mean values per animal are represented by dots.

Data information: Control: animals left untreated (*n* = 2); Chemo + DMSO: animals receiving chemotherapy and vehicle (*n* = 4); Chemo + ICG-001: animals receiving chemotherapy and the β-catenin inhibitor ICG-001 (*n* = 4), following scheme and doses depicted in Fig 6A and methods.
Data information: For all applicable figure panels, data from Chemo + DMSO and Chemo + ICG-001 groups are mean ± SD and dots represent individual values per animal. Control group data is only represented by individual values per animal. Statistical significance between Chemo + DMSO and Chemo + ICG-001 groups was determined by two-sided Student's *t*-test.
Source data are available online for this figure.

have improved survival in pediatric patients up to 90% (e.g., TARGET cohort), however relapse and refractory patients still have a dismal outcome, and adult patient DFS is very poor compared with children (Ribera *et al*, 2021). We found that the β-catenin signature is informative for the identification of refractory patients in three different cohorts (two from COG group, one from EGA study and one from TARGET). Unfortunately, all cohorts with gene expression data include very few refractory patients. Further studies with better annotated cohorts should be performed in the future to further confirm the usefulness of this signature and whether it can be refined.

We also found a significant association between the phenotypes ETP + ABD with the outcome in the discovery cohort (HR = 7.64; *P* = 0.03), being all the refractory cases inside these T-ALL subtypes (Fig 3E). ETP and ABD groups are both immature T-ALLs and patients falling into these phenotypes frequently display a dismal outcome and higher rates of chemotherapy refraction (Coustan-Smith *et al*, 2009; Gutierrez *et al*, 2010). In this study, we observed that the minimal β-catenin signature is specifically informative of therapy failure even inside the group of patients with an immature (poor prognosis) phenotype (Fig 3A) but it represents a more powerful prognosis factor than ETP + ABD alone (HR cluster P1 = 18.18; *P* = 0.003 vs. HR ETP + ABD = 7.64; *P* = 0.03; Fig 3E). Whether the β-catenin activity contributes to the worse outcome of immature T-ALLs requires further research.

By ChIPseq and RNAseq analysis we found that β-catenin regulates RNA processing and RNA biogenesis genes. Our analysis showed that non-responder patients concentrated in a group expressing a specific β-catenin-dependent RNA processing signature, which had not been previously identified (van Loosdregt *et al*, 2013; Doumpas *et al*, 2019). These genes are transcriptionally active in T-ALL cells and their expression is reduced when β-catenin is knocked-down. Enriched RNA processing functions include different types of RNA-binding proteins (RBP). Aberrant RBPs functioning may result in global remodeling of the transcriptome and the proteome and it has been implicated in human disease and tumorigenesis (reviewed in Hodson *et al*, 2019; Mohibi *et al*, 2019). In AML, a comprehensive CRISPR screening uncovered the upregulation of a network of RBPs important for AML survival and identified RBM39 as a critical and targetable element (Wang *et al*, 2019). Here, we also detected transcriptional downregulation of RBM39 in β-catenin knockdown cells, although it was not identified as a direct β-catenin chromatin target. However, since post-translational modifications may affect the protein levels of RBPs, further analysis of these proteins in the absence of β-catenin should clarify its involvement in chemotherapy resistance.

Although different β-catenin chromatin partners have been described, the TCF/LEF family of transcription factors are the most widely used. Despite not finding enrichment of the TCF/LEF canonical motif, β-catenin sits at the promoter regions, in a DNA-binding motif that has been assigned to ZBTB33/Kaiso. TCF1, LEF1, ZBTB33/Kaiso as well as β-catenin can all bind to these promoters. Although ZBTB33/Kaiso was first shown to have a repressive function in β-catenin target-gene, we found that knockdown of ZBTB33/Kaiso reduces the expression of selected β-catenin target genes and decreases the binding of β-catenin. The COGP9404 cohort shows that slightly higher β-catenin and ZBTB33/Kaiso levels tend to be associated with the group of patients with the worst outcome, whereas levels of TCF1 and LEF1 are significantly lower. These results suggest that TCF1/LEF1 and ZBTB33/Kaiso may collaborate with β-catenin in activating gene transcription in different complexes that may result in distinct types of activities. We observed that β-catenin is able to interact with ZBTB33/Kaiso in the nucleus, however the composition of the activating complex requires further work.

We have also examined whether other factors important for T-ALL are able to collaborate with β-catenin in the chromatin. Computational comparison of the β-catenin target regions with regions bound by T-ALL factors (TAL1, GATA3, LMO1, Runx1 from Sanda *et al* (2012) and Notch1 from https://chip-atlas.org) showed a significant coincidence between β-catenin- and Runx1- or Notch1-bound regions (13.9 and 9.2% of overlapping peaks, respectively, adjusted *P* = 0.008). Contribution of these factors to β-catenin-dependent transcription will be further investigated.

We previously found that β-catenin and Notch are required for MYC activation in T-ALL cells (Gekas *et al*, 2016). Among multiple crucial functions, MYC is involved in RNA metabolism (mRNA splicing, stability and translation efficiency), which is necessary for

 © 2023 The Authors

tumor cells growth (Koh *et al*, 2016; Bigas *et al*, 2020). However, we now find that mRNA processing genes are direct targets of β-catenin. Genes controlled by β-catenin do not have Myc DNA-binding motifs (Fig 1C and Dataset EV1), however we cannot exclude that some genes are coregulated by both factors. Our data indicates that β-catenin regulates a set of RNA processing genes, indicating that MYC and β-catenin regulated genes converge in the RNA function as key factors for T-ALL progression and response to chemotherapy.

Materials and Methods

Cell lines

RPMI8402 (DSMZ ACC. 290), Jurkat (DSMZ ACC. 282), CCRF-CEM (ATCC CCL-119), DND-41 (DSMZ ACC. 525) and HEK 293 T (ATCC 3216) human cell lines were cultured in standard conditions and regularly tested for Mycoplasma contamination. Negative contamination status was confirmed by PCR before each experiment.

Reagents

LiCl (Sigma, 203637) was used at 25 mM. FH-535 (Tocris, 4344) and ICG-001 (Tocris, 4505) stocks were diluted in dimethyl sulfoxide (DMSO, Sigma) and used at the indicated concentrations. Vincristine (Pfizer, 1 mg ml^{-1}) and dexamethasone (Fortecortin, 4 mg ml^{-1}) were kindly provided by Hospital del Mar. L-Asparaginase was obtained from MedChemExpress (HY-P1923).

RNA isolation and sequencing

RNA was isolated using RNeasy Mini Kit (Qiagen) and retro-transcribed using Transcriptor First Strand cDNA Synthesis Kit (Roche). cDNA is used for qPCR analysis. Primers used are listed in Appendix Table S2.

Total RNA from three biological replicates of RPMI8402 sh-control and sh β-catenin were sequenced in the Genomics facility from Centre for Genomic Regulation (CRG) using Illumina Nextseq500 platform. Raw paired-end 150-bp sequences were filtered by quality ($Q > 30$) and length (length > 20 bp) with TrimGalore. Filtered sequences were aligned against the reference genome (hg38) with Bowtie2. High-quality alignments were fed to HTSeq (Anders *et al*, 2015) to estimate the normalized counts of each expressed gene. Differentially expressed genes between different conditions were explored using DESeq2 R package (Love *et al*, 2014).

Chromatin immunoprecipitation and analysis

Cells were crosslinked in 0.2 mM di-succynimidyl glutarate (DSG, Sigma, 80424) for 10 min (Estaras *et al*, 2015) followed by 0.5% formaldehyde for 10 min. Chromatin was isolated by lysing the cells in 100 mM Tris–HCl at pH 8, 0.25% Triton X-100, 100 mM EDTA at pH 8, 0.5 mM EGTA, 20 mM β-Glycerol-phosphate, 0.1 mM NaOrtovanadate and 10 mM NaButyrate and centrifuging 800 *g* to isolate the nuclear pellet, which is sonicated in 10 mM Tris–HCl at pH 8, 100 mM NaCl, 1 mM EDTA at pH 8, 0.63 mM EGTA, 10 mM NaButyrate, 20 mM β-Glycerol-phosphate, 0.1 mM NaOrtovanadate and 1% SDS for 7 cycles, 30 s ON and 30 s OFF, in a Bioruptor Pico (Diagenode). Chromatin is concentrated using Vivaspin 20 columns (Sartorius) and pre-cleared by adding 1% BSA, 12.5 μg μl^{-1} of salmon sperm DNA, 2.5–10 μg of irrelevant IgG (depending on the experimental antibody) and protein G/A-Sepharose. After pre-clearing, chromatin fragments were incubated overnight with irrelevant IgG or the indicated antibodies and precipitated with protein G/A-Sepharose. Crosslink was reversed overnight at 65°C and protein was degraded using 460 μg μl^{-1} of Proteinase K (Roche) for 2 h at 55°C. Chromatin was purified using the MiniElute PCR Purification Kit (Qiagen) and used either for sequencing or for quantitative PCR (qPCR). Fold enrichment was calculated relative to irrelevant IgG. Primers used are listed in Appendix Table S2.

Chromatin was sequenced in the Genomics facility from Centre for Genomic Regulation (CRG) using Illumina HiSeq platform. Raw single-end 50-bp sequences were filtered by quality ($Q > 30$) and length (length > 20 bp) with TrimGalore. Filtered sequences were aligned against the reference genome (hg38) with Bowtie2 (Langmead & Salzberg, 2012). BigWig files were generated with bamCoverage from deepTools (Ramirez *et al*, 2016). Resulting data was visualized through the IGV software (Robinson *et al*, 2011). MACS2 (Zhang *et al*, 2008) was used to identify peaks (using a *P*-value < 1e-5) normalizing to an input sequence from the same cell lines. Peaks were annotated with ChIPseeker (Yu *et al*, 2015). Raw and processed data was uploaded to GEO (GSE196986). Functional enrichment analysis was performed with enrichR (Kuleshov *et al*, 2016) R package against "GO Biological Process 2021" database (Gene Ontology Consortium, 2021). Motif enrichment analysis was performed using MEME-ChIP software (Machanick & Bailey, 2011; https://maayanlab.cloud/Enrichr/Genomicdatasets).

T-ALL Histone marks ChIPseq data are from GSE59657, GSE51522, GSE29611, GSE35583, GSE85601, GSE59257. New ChIPseq and RNAseq data is deposited in GEO (GSE196986).

Lentiviral production

Recombinant lentiviruses were produced in the HEK293T cell line (ATCC Ref. CRL-3216). Plasmids for lentiviral production (#8455 and #12259, Addgene) were introduced together with the desired lentiviral plasmid using polyethyleneimine. Supernatant was filtered with 45 μM filters and ultracentrifuged at 75,000 *g* for 3 h.

Genetic editing

sgRNAs were designed using Benchling (Uniyal *et al*, 2019; Biology Software) and cloned in the lentiCRISPR v2 plasmid (Addgene, #52961). Selected cells were isolated and cultured as single-cells and screened by WB. Knockout clones identified by WB were further verified by Sanger sequencing. sgRNAs used are listed in Appendix Table S1.

shβCat (pLKO.1-Hygro, Sigma, Mission shRNA ID #TRCN0000314921 (Aulicino *et al*, 2014)), shKaiso (pLKO.1-Puro, Sigma, MissionshRNA ID #TRCN000017838) or scrambled control (shControl; pLKO.1-Puro, Sigma, Mission shRNA ID #SHC016) were used to transduce T-ALL cells using lentiviral particles and selected with Hygromycin 800 μg μl^{-1} (Invivogen) or with Puromycin 1.25 μg ml^{-1} (Sigma).

© 2023 The Authors

Western blotting (WB)

Cells were lysed with 10 mM HEPES, 1.5 mM $MgCl_2$, 10 mM KCl, 0.05% NP40, protease inhibitors (Roche) for 10 min at 4°C, centrifuged at 800 *g* and analyzed by electrophoresis. Antibodies used are listed in Appendix Table S3.

RNA and protein synthesis analysis

Click-iT Plus OPP Alexa Fluor 488 Protein Synthesis Assay Kit or Click-iT RNA Alexa Fluor 488 Imaging Kit (Invitrogen) were used for protein and RNA synthesis determination, respectively, following the manufacturer's instructions. Cells were analyzed with the LSRII Cytometer (BD Biosciences). Data analysis was performed using FlowJo.

Cell cycle analysis

Cell cycle was determined by flow cytometry using Ki67-APC and DAPI intracellular stainings. Unsynchronized RPMI8402 cells were collected upon β-Catenin knockdown or after 24 and 48 h-treatment with the β-Catenin inhibitors ICG-001 (10 μM) or FH535 (30 μM). Fixed and permeabilized cells (FIX&Perm Cell permeabilization kit, Invitrogen GAS004) were stained with Ki67-AF647 (1/100, 20 min in the dark; clone B56, BD Bioscience 558615) and DAPI (5 μg ml^{-1}, 1 h in the dark). Cells were analyzed with the Fortessa Cytometer (BD Biosciences). Data analysis was performed using FlowJo.

Gene expression in T-ALL primary samples

The transcriptomes of T-ALL patients were from GEO (GSE14618, including COG study 9404 and COG study 8704), EGA (EGAS00001000536) and Therapeutically Applicable Research to Generate Effective Treatments (TARGET) initiative, phs000218. (https://ocg.cancer.gov/programs/target) repositories. Informed consent was obtained from all subjects and experiments conformed to the principles set out in the WMA Declaration of Helsinki and the Department of Health and Human Services Belmont Report. The transcriptomes of primary samples from GSE14618 obtained by microarrays were analyzed with the affy and limma R package. Data from COG studies 9404 (50 patients) and 8704 (42 patients) were processed independently. Only 40 out of the 50 primary samples from COG study 9404 had available survival clinical data and were further used as a discovery cohort for the identification of predictive signatures. The discovery cohort included 21 complete remission, 13 relapses and six refractory patients and were classified into the phenotypes ETP (Early T-cell precursor), ABD (absence of biallelic TCRgamma locus deletion), LEF1 negative, TAL1, and TLX1. The corresponding clinical data was provided by Alejandro Gutiérrez (Dana Faber, USA). COG 8707 contained 42 patients with no survival data and was comprised by 24 remission, 17 relapsed and one refractory patient.

A total of 265 RNASeq samples from the TARGET cohort were downloaded, including the clinical data. Patients with censored or no outcome information and those affected by a second malignant neoplasm were excluded from the analysis. Data used for further interrogation comprised 230 remissions, 20 relapses, two patients categorized as death and a single patient classified as disease progression (31 days of DFS). Additional 27 RNASeq samples published by Atak *et al* (2013) were downloaded from the European Genome-phenome Archive (EGAS00001000536). Raw sequences from TARGET and EGAS0000100536 datasets were mapped against the human genome using vast-tools (Tapial *et al*, 2017) to assess the normalized expression levels (cRPKM) of β-catenin targets. Primary patients were distributed in 10 remissions, 12 relapses, one refractory patient and four labeled as dead.

Patients were clustered using a non-supervised hierarchical model based on the expression of different groups of β-catenin targets. All non-supervised hierarchical clusterings included were obtained using the pheatmap R package with default parameters (euclidean distance and complete clustering method). In each case, the number of patient and gene clusters were decided based on visual inspection of different expression patterns among clusters. In 3A, we produced a minimal predictive β-catenin signature and we adjusted the number of patient clusters to include the majority of refractory cases within the same cluster. Survival curves were performed with the survminer R package. Hazard ratios were estimated with the peperr R package. Functional enrichment analysis was performed with enrichR (Kuleshov *et al*, 2016) R package against "GO Biological Process 2021" database (Gene Ontology Consortium, 2021).

Single-sample gene set enrichment analysis (ssGSEA)

Gene signatures from gene clusters G1 and G2 were used to derive single-sample gene set enrichment (ssGSEA) scores. Normalized gene expression data for the GSE14618 (COG9404 and COG8707 studies), EGA and TARGET cohorts were submitted to the GenePattern platform (Reich *et al*, 2006). ssGSEA module (version 10.0.11) was used to calculate individual enrichment scores for each pairing patient-gene set. Weighting exponent was set to 0.75 and minimum gene set size was maintained to 10.

Chemotherapy response-assays

T-ALL cell lines (25,000–35,000 cells per well in 96-well plates) were incubated with the indicated drugs for 48 h and cell viability was monitored by the MTT assay (Sigma, M2003). MTT absorbance of the cells incubated in the vehicle (untreated) was set to 100% viability to calculate the dose–response curves. We used GraphPad Prism 8 to generate the logistic fitting curve by non-linear regression model and to calculate IC50 for each experiment. Treatments were performed in triplicate and repeated three times.

In vitro drug recovery assays

We followed the strategy depicted in Fig 5D and G. After a 2-days treatment, cells were washed twice in PBS to wash-out the drugs and re-seeded for recovery either in complete medium without drugs or in the presence of 10 μM of the β-catenin inhibitor ICG-001 that is replenished after the wash-out. After a 3-day recovery period, cells were again washed twice with PBS to remove the β-catenin inhibitor and the remaining cells were maintained in complete medium for an additional 5-day period to evaluate the final recovery. Cell viability was determined by the metabolic activity by MTT

© 2023 The Authors

assays at days 0, 2, 5 and 10 from treatment initiation (corresponding to days −2, 0, 3 and 5 from chemotherapy wash-out). Cell viability was normalized to the MTT absorbance of the cells at day 0.

Animal studies

Animal work was conducted under pathogen-free conditions and adhered to the guidelines from Generalitat de Catalunya and the ethics committee at Parc de Recerca Biomèdica de Barcelona (approved protocol number 10655 according to the European Union regulations).

NSG mice (strain: ANB//NOD.Cg-Prkdcscid Il2rgtm1Wjl/SzJ; 2-month-old males and females) were sublethally irradiated (2 Gy) and retro-orbitally transplanted with 5×10^5 RPMI8402 cells. Animals were randomized into two treatment groups: animals receiving DMSO vehicle and chemotherapy ("Chemo + DMSO", $n = 4$), and animals receiving ICG-001 and chemotherapy ("Chemo + ICG-001", $n = 4$). Two more animals were left untreated as controls to monitor the disease. Three days after transplantation, animals were treated with an induction-like chemotherapy combination (VDL, V (vincristine, 0.15 mg kg^{-1} day^{-1}; days 0 and 7), D (dexamethasone, 5 mg kg^{-1} day^{-1}; days 0, 5, 7 and 8), L (L-Asparaginase, 1,000 IU kg^{-1} day^{-1}; days 0, 5, 7 and 8); Oshima *et al*, 2020). ICG-001 (10 mg kg^{-1} day^{-1}) or DMSO vehicle were administered daily (except days of chemotherapy administration) for 2 weeks and every 2 days for 1 week. Stock solutions of drugs were diluted in sterile saline (chemotherapy) or in 40% PEG300, 5% Tween-80, 55% saline (ICG-011 and DMSO) and administered intraperitoneally (ip). See Fig 6A for treatment scheme. Peripheral blood (PB) was analyzed weekly by flow cytometry for the detection of RPMI8402 cells. Animal were euthanized after 3 weeks when first animals reached a humane endpoint. PB, bone marrow (BM), liver and spleen were collected and evaluated by flow cytometry and hematoxylin and eosin (H&E) stainings (liver).

Flow cytometry analysis of mice organs

Antibodies used for flow cytometry analysis are listed in Appendix Table S4. Cells were analyzed with the LSRII Cytometer (BD Biosciences). Data analysis was performed using FlowJo.

Colony quantification in H&E stainings

ImageJ version 1.53t was used to visualize and quantify cellular colonies. Acquired images were transform into 8-bit greyscale format. After uniform setting of intensity threshold, the colony area is automatically calculated. Four different 10× fields were measured for each animal. Manual counting of the number of cellular colonies was also performed.

Statistical analysis

No statistical methods were used to determine the sample size. The experiments were not randomized and the investigator were not blinded.

GraphPad Prism 8 software and R software environment were used for statistical analysis. Statistical parameters and significance are indicated in the figures and legends. For qPCR experiments and *in vitro* drugs assays, statistical significance among groups was determined by Student's *t*-test (data fitting normal distribution) or Mann–Whitney *U* test (data not fitting normal distribution) for two-group comparison or one-way ANOVA with Tukey's correction.

The paper explained

Problem
T-cell Acute Lymphoblastic Leukemia (T-ALL) is a rare disease affecting children and adults. Combinations of high-dose chemotherapeutic drugs have greatly improved the outcome of pediatric patients, but adults and the few children that relapse have still very dismal prognosis and targeted therapies are not yet available.

Results
Characterization of β-catenin DNA-binding sites and transcriptional targets in T-ALL cell lines identified a β-catenin-dependent gene signature, involving multiple RNA processing elements, that is associated with initial induction failure in T-ALL patients. Consistent with this observation, inhibition of β-catenin sensitizes T-ALL cells to chemotherapy treatments *in vitro* and *in vivo*.

Impact
Our results indicate that β-catenin positively regulates chemotherapy resistance, and suggest that specific β-catenin-dependent activities could be used for the stratification of patients with higher risk of induction failure. These same patients could benefit from combination treatments including β-catenin inhibitors plus chemotherapy.

Data availability

- ChIP seq and RNA seq data: Gene Expression Omnibus GSE196986 (https://www.ncbi.nlm.nih.gov/geo/query/acc.cgi?&acc=GSE196986).
- Transcriptomic Public data sets:COG study 9404 and 8704: GSE14618.EGA cohort: EGAS00001000536; https://ega-archive.org/studies/EGAS00001000536TARGET: https://ocg.cancer.gov/programs/target
- Computational codes available at https://github.com/vgarciahern/Garcia-Hernandez_V_et_al_EMBOMM_manuscript.git

Expanded View for this article is available online.

Acknowledgements
We would like to acknowledge all members of the Bigas and Espinosa lab for critical discussions of this work. Alejandro Gutierrez (Dana Farber, Boston) for sharing clinical data, Susana de la Luna (CRG) and Antonio Salar (IMIM, Hospital del Mar) and Pablo Menéndez (IJC) for kindly providing important reagents and advice. We thank CRG Genomics Unit, UPF Flow Cytometry Core Facility and PRBB Animal Facility for their assistance. This work has been funded by Agencia Estatal de Investigación (SAF2016-75613-R and PID2019-104695RB-I00), Fundación AECC (GC16173697BIGA) and WCR (13-0064). VGH, DA and TL are recipients of Sara Borrell fellowship from ISCIII co-funded by the ESF+ (CD21/00145), FPI (BES-2017-080880) and AECC fellowship (POSTD21975LOBO), respectively.

Author contributions
Violeta García-Hernández: Conceptualization; data curation; formal analysis; supervision; validation; investigation; methodology; writing – original draft;

© 2023 The Authors

writing – review and editing. **David Arambilet:** Formal analysis; investigation. **Yolanda Guillén:** Conceptualization; data curation; software; formal analysis; writing – original draft. **Teresa Lobo-Jarne:** Data curation; software; formal analysis. **María Maqueda:** Resources; data curation; software; formal analysis; writing – review and editing. **Christos Gekas:** Investigation; methodology. **Jessica González:** Investigation. **Arnau Iglesias:** Investigation. **Nerea Vega-García:** Resources; investigation. **Inés Sentís:** Resources; formal analysis. **Juan L Trincado:** Resources; formal analysis. **Ian Márquez-López:** Investigation. **Holger Heyn:** Resources; supervision. **Mireia Camós:** Resources; supervision. **Lluis Espinosa:** Conceptualization; supervision; methodology; writing – original draft; writing – review and editing. **Anna Bigas:** Conceptualization; supervision; funding acquisition; visualization; methodology; writing – original draft; project administration; writing – review and editing.

Disclosure and competing interests statement

The authors declare that they have no conflict of interest.

References

Anders S, Pyl PT, Huber W (2015) HTSeq—a python framework to work with high-throughput sequencing data. *Bioinformatics* 31: 166 – 169

Asselin BL, Devidas M, Wang C, Pullen J, Borowitz MJ, Hutchison R, Lipshultz SE, Camitta BM (2011) Effectiveness of high-dose methotrexate in T-cell lymphoblastic leukemia and advanced-stage lymphoblastic lymphoma: a randomized study by the Children's Oncology group (POG 9404). *Blood* 118: 874 – 883

Atak ZK, Gianfelici V, Hulselmans G, De Keersmaecker K, Devasia AG, Geerdens E, Mentens N, Chiaretti S, Durinck K, Uyttebroeck A *et al* (2013) Comprehensive analysis of transcriptome variation uncovers known and novel driver events in T-cell acute lymphoblastic leukemia. *PLoS Genet* 9: e1003997

Aulicino F, Theka I, Ombrato L, Lluis F, Cosma MP (2014) Temporal perturbation of the Wnt signaling pathway in the control of cell reprogramming is modulated by TCF1. *Stem Cell Reports* 2: 707 – 720

Barbie DA, Tamayo P, Boehm JS, Kim SY, Moody SE, Dunn IF, Schinzel AC, Sandy P, Meylan E, Scholl C *et al* (2009) Systematic RNA interference reveals that oncogenic KRAS-driven cancers require TBK1. *Nature* 462: 108 – 112

Barna M, Pusic A, Zollo O, Costa M, Kondrashov N, Rego E, Rao PH, Ruggero D (2008) Suppression of Myc oncogenic activity by ribosomal protein haploinsufficiency. *Nature* 456: 971 – 975

Bigas A, Guillen Y, Schoch L, Arambilet D (2020) Revisiting beta-catenin signaling in T-cell development and T-cell acute lymphoblastic leukemia. *Bioessays* 42: e1900099

Bigas A, Rodriguez-Sevilla JJ, Espinosa L, Gallardo F (2022) Recent advances in T-cell lymphoid neoplasms. *Exp Hematol* 106: 3 – 18

Bugter JM, Fenderico N, Maurice MM (2021) Publisher correction: mutations and mechanisms of WNT pathway tumour suppressors in cancer. *Nat Rev Cancer* 21: 64

Carter BZ, Mak PY, Wang X, Tao W, Ruvolo V, Mak D, Mu H, Burks JK, Andreeff M (2019) An ARC-regulated IL1beta/Cox-2/PGE2/beta-catenin/ARC circuit controls leukemia-microenvironment interactions and confers drug resistance in AML. *Cancer Res* 79: 1165 – 1177

Coustan-Smith E, Mullighan CG, Onciu M, Behm FG, Raimondi SC, Pei D, Cheng C, Su X, Rubnitz JE, Basso G *et al* (2009) Early T-cell precursor leukaemia: a subtype of very high-risk acute lymphoblastic leukaemia. *Lancet Oncol* 10: 147 – 156

De Jaime-Soguero A, Aulicino F, Ertaylan G, Griego A, Cerrato A, Tallam A, Del Sol A, Cosma MP, Lluis F (2017) Wnt/Tcf1 pathway restricts embryonic stem cell cycle through activation of the Ink4/Arf locus. *PLoS Genet* 13: e1006682

Dose M, Emmanuel AO, Chaumeil J, Zhang J, Sun T, Germar K, Aghajani K, Davis EM, Keerthivasan S, Bredemeyer AL *et al* (2014) Beta-catenin induces T-cell transformation by promoting genomic instability. *Proc Natl Acad Sci USA* 111: 391 – 396

Doumpas N, Lampart F, Robinson MD, Lentini A, Nestor CE, Cantu C, Basler K (2019) TCF/LEF dependent and independent transcriptional regulation of Wnt/beta-catenin target genes. *EMBO J* 38: e98873

Dunsmore KP, Winter SS, Devidas M, Wood BL, Esiashvili N, Chen Z, Eisenberg N, Briegel N, Hayashi RJ, Gastier-Foster JM *et al* (2020) Children's Oncology group AALL0434: a phase III randomized clinical trial testing Nelarabine in newly diagnosed T-cell acute lymphoblastic leukemia. *J Clin Oncol* 38: 3282 – 3293

Estaras C, Benner C, Jones KA (2015) SMADs and YAP compete to control elongation of beta-catenin:LEF-1-recruited RNAPII during hESC differentiation. *Mol Cell* 58: 780 – 793

Gang EJ, Hsieh YT, Pham J, Zhao Y, Nguyen C, Huantes S, Park E, Naing K, Klemm L, Swaminathan S *et al* (2014) Small-molecule inhibition of CBP/catenin interactions eliminates drug-resistant clones in acute lymphoblastic leukemia. *Oncogene* 33: 2169 – 2178

Gekas C, D'Altri T, Aligue R, Gonzalez J, Espinosa L, Bigas A (2016) Beta-catenin is required for T-cell leukemia initiation and MYC transcription downstream of Notch1. *Leukemia* 30: 2002 – 2010

Giambra V, Jenkins CE, Lam SH, Hoofd C, Belmonte M, Wang X, Gusscott S, Gracias D, Weng AP (2015) Leukemia stem cells in T-ALL require active Hif1alpha and Wnt signaling. *Blood* 125: 3917 – 3927

Gutierrez A, Dahlberg SE, Neuberg DS, Zhang J, Grebliunaite R, Sanda T, Protopopov A, Tosello V, Kutok J, Larson RS *et al* (2010) Absence of biallelic TCRgamma deletion predicts early treatment failure in pediatric T-cell acute lymphoblastic leukemia. *J Clin Oncol* 28: 3816 – 3823

Handeli S, Simon JA (2008) A small-molecule inhibitor of Tcf/beta-catenin signaling down-regulates PPARgamma and PPARdelta activities. *Mol Cancer Ther* 7: 521 – 529

Hodson DJ, Screen M, Turner M (2019) RNA-binding proteins in hematopoiesis and hematological malignancy. *Blood* 133: 2365 – 2373

Kaur A, Lim JYS, Sepramaniam S, Patnaik S, Harmston N, Lee MA, Petretto E, Virshup DM, Madan B (2021) WNT inhibition creates a BRCA-like state in Wnt-addicted cancer. *EMBO Mol Med* 13: e13349

Kaveri D, Kastner P, Dembele D, Nerlov C, Chan S, Kirstetter P (2013) Beta-catenin activation synergizes with Pten loss and Myc overexpression in Notch-independent T-ALL. *Blood* 122: 694 – 704

Koh CM, Sabo A, Guccione E (2016) Targeting MYC in cancer therapy: RNA processing offers new opportunities. *Bioessays* 38: 266 – 275

Kuleshov MV, Jones MR, Rouillard AD, Fernandez NF, Duan Q, Wang Z, Koplev S, Jenkins SL, Jagodnik KM, Lachmann A *et al* (2016) Enrichr: a comprehensive gene set enrichment analysis web server 2016 update. *Nucleic Acids Res* 44: W90 – W97

Langmead B, Salzberg SL (2012) Fast gapped-read alignment with bowtie 2. *Nat Methods* 9: 357 – 359

Love MI, Huber W, Anders S (2014) Moderated estimation of fold change and dispersion for RNA-seq data with DESeq2. *Genome Biol* 15: 550

Machanick P, Bailey TL (2011) MEME-ChIP: motif analysis of large DNA datasets. *Bioinformatics* 27: 1696 – 1697

Mohibi S, Chen X, Zhang J (2019) Cancer the 'RBP'eutics-RNA-binding proteins as therapeutic targets for cancer. *Pharmacol Ther* 203: 107390

© 2023 The Authors

Ng OH, Erbilgin Y, Firtina S, Celkan T, Karakas Z, Aydogan G, Turkkan E, Yildirmak Y, Timur C, Zengin E *et al* (2014) Deregulated WNT signaling in childhood T-cell acute lymphoblastic leukemia. *Blood Cancer J* 4: e192

Gene Ontology Consortium (2021) The Gene Ontology resource: enriching a GOld mine. *Nucleic Acids Res* 49: D325 – D334

Oshima K, Zhao J, Perez-Duran P, Brown JA, Patino-Galindo JA, Chu T, Quinn A, Gunning T, Belver L, Ambesi-Impiombato A *et al* (2020) Mutational and functional genetics mapping of chemotherapy resistance mechanisms in relapsed acute lymphoblastic leukemia. *Nat Cancer* 1: 1113 – 1127

Park JI, Kim SW, Lyons JP, Ji H, Nguyen TT, Cho K, Barton MC, Deroo T, Vleminckx K, Moon RT *et al* (2005) Kaiso/p120-catenin and TCF/beta-catenin complexes coordinately regulate canonical Wnt gene targets. *Dev Cell* 8: 843 – 854

Perry JM, Tao F, Roy A, Lin T, He XC, Chen S, Lu X, Nemechek J, Ruan L, Yu X *et al* (2020) Overcoming Wnt-beta-catenin dependent anticancer therapy resistance in leukaemia stem cells. *Nat Cell Biol* 22: 689 – 700

Ramirez F, Ryan DP, Gruning B, Bhardwaj V, Kilpert F, Richter AS, Heyne S, Dundar F, Manke T (2016) deepTools2: a next generation web server for deep-sequencing data analysis. *Nucleic Acids Res* 44: W160 – W165

Reich M, Liefeld T, Gould J, Lerner J, Tamayo P, Mesirov JP (2006) GenePattern 2.0. *Nat Genet* 38: 500 – 501

Ribera JM, Morgades M, Genesca E, Chapchap EC, Montesinos P, Acuna-Cruz E, Gil C, Garcia-Cadenas I, Barba P, Gonzalez-Campos J *et al* (2021) Outcomes and prognostic factors of adults with refractory or relapsed T-cell acute lymphoblastic leukemia included in measurable residual disease-oriented trials. *Hematol Oncol* 39: 529 – 538

Robinson JT, Thorvaldsdottir H, Winckler W, Guttman M, Lander ES, Getz G, Mesirov JP (2011) Integrative genomics viewer. *Nat Biotechnol* 29: 24 – 26

Roy S, Kar M, Roy S, Saha A, Padhi S, Banerjee B (2018) Role of beta-catenin in cisplatin resistance, relapse and prognosis of head and neck squamous cell carcinoma. *Cell Oncol (Dordr)* 41: 185 – 200

Sanda T, Lawton LN, Barrasa MI, Fan ZP, Kohlhammer H, Gutierrez A, Ma W, Tatarek J, Ahn Y, Kelliher MA *et al* (2012) Core transcriptional regulatory circuit controlled by the TAL1 complex in human T cell acute lymphoblastic leukemia. *Cancer Cell* 22: 209 – 221

Tapial J, Ha KCH, Sterne-Weiler T, Gohr A, Braunschweig U, Hermoso-Pulido A, Quesnel-Vallieres M, Permanyer J, Sodaei R, Marquez Y *et al* (2017) An atlas of alternative splicing profiles and functional associations reveals new regulatory programs and genes that simultaneously express multiple major isoforms. *Genome Res* 27: 1759 – 1768

Tetsu O, McCormick F (1999) Beta-catenin regulates expression of cyclin D1 in colon carcinoma cells. *Nature* 398: 422 – 426

Tzoneva G, Ferrando AA (2012) Recent advances on NOTCH signaling in T-ALL. *Curr Top Microbiol Immunol* 360: 163 – 182

Uniyal AP, Mansotra K, Yadav SK, Kumar V (2019) An overview of designing and selection of sgRNAs for precise genome editing by the CRISPR-Cas9 system in plants. *3 Biotech* 9: 223

van de Wetering M, Sancho E, Verweij C, de Lau W, Oving I, Hurlstone A, van der Horn K, Batlle E, Coudreuse D, Haramis AP *et al* (2002) The beta-catenin/TCF-4 complex imposes a crypt progenitor phenotype on colorectal cancer cells. *Cell* 111: 241 – 250

van Loosdregt J, Fleskens V, Fu J, Brenkman AB, Bekker CP, Pals CE, Meerding J, Berkers CR, Barbi J, Grone A *et al* (2013) Stabilization of the transcription factor Foxp3 by the deubiquitinase USP7 increases Treg-cell-suppressive capacity. *Immunity* 39: 259 – 271

Wang E, Lu SX, Pastore A, Chen X, Imig J, Chun-Wei Lee S, Hockemeyer K, Ghebrechristos YE, Yoshimi A, Inoue D *et al* (2019) Targeting an RNA-binding protein network in acute myeloid leukemia. *Cancer Cell* 35: 369 – 384

Winter SS, Jiang Z, Khawaja HM, Griffin T, Devidas M, Asselin BL, Larson RS, Children's Oncology Group (2007) Identification of genomic classifiers that distinguish induction failure in T-lineage acute lymphoblastic leukemia: a report from the Children's Oncology group. *Blood* 110: 1429 – 1438

Xu Z, Xing S, Shan Q, Gullicksrud JA, Bair TB, Du Y, Liu C, Xue HH (2017) Cutting edge: beta-catenin-interacting Tcf1 isoforms are essential for Thymocyte survival but dispensable for Thymic maturation transitions. *J Immunol* 198: 3404 – 3409

Yu S, Zhou X, Steinke FC, Liu C, Chen SC, Zagorodna O, Jing X, Yokota Y, Meyerholz DK, Mullighan CG *et al* (2012) The TCF-1 and LEF-1 transcription factors have cooperative and opposing roles in T cell development and malignancy. *Immunity* 37: 813 – 826

Yu G, Wang LG, He QY (2015) ChIPseeker: an R/Bioconductor package for ChIP peak annotation, comparison and visualization. *Bioinformatics* 31: 2382 – 2383

Zhang Y, Liu T, Meyer CA, Eeckhoute J, Johnson DS, Bernstein BE, Nusbaum C, Myers RM, Brown M, Li W *et al* (2008) Model-based analysis of ChIP-Seq (MACS). *Genome Biol* 9: R137

Zhou H, Mak PY, Mu H, Mak DH, Zeng Z, Cortes J, Liu Q, Andreeff M, Carter BZ (2017) Combined inhibition of beta-catenin and Bcr-Abl synergistically targets tyrosine kinase inhibitor-resistant blast crisis chronic myeloid leukemia blasts and progenitors in vitro and in vivo. *Leukemia* 31: 2065 – 2074

Zhu H, Zhang L, Wu Y, Dong B, Guo W, Wang M, Yang L, Fan X, Tang Y, Liu N *et al* (2018) T-ALL leukemia stem cell 'stemness' is epigenetically controlled by the master regulator SPI1. *Elife* 7: e38314

License: This is an open access article under the terms of the Creative Commons Attribution License, which permits use, distribution and reproduction in any medium, provided the original work is properly cited.

Expanded View Figures

Figure EV1. β-catenin-binding genes in T-ALL cell lines.

A, B WB analysis of the indicated proteins from cytoplasmic and nuclear fractions of four different T-ALL cell lines (A) and at different times of 25 mM LiCl treatment in RPMI8402 cells (B). Tubulin and H3 detection were used as loading and fractionation controls.

C WB analysis of precipitated β-catenin in RPMI8402 cells crosslinked with 0.2 mM DSG at different time points, as done in the ChIP protocol.

D Venn diagrams showing the overlap between β-catenin-enriched ChIP-targets in basal vs LiCl treated conditions obtained in at least two replicates.

E IGV representation of β-catenin-enriched peaks in five basal and two LiCl treated replicates.

F Functional enrichment analysis of β-catenin ChIP targets. Statistically enriched categories (adjusted *P*-value < 0.05) among GO Biological Process terms.

G qPCR detection of β-catenin-enriched genes by ChIP in basal and LiCl treated conditions in the RPMI8402 (left panel) and in the Jurkat (right panel) cell lines. qPCR results normalized to negative IgG control indicated by a red-dashed line. Graph represents the mean and SD of three technical replicates.

H Venn diagrams showing the overlap between the β-catenin, TCF1 and LEF1 ChIPenriched genes (left panel) or the overlap between the β-catenin and the Kaiso genes obtained in at least two replicates.

I WB analysis of Kaiso, TCF1 and LEF1 in control (Empty Vector, EV) and Kaiso KO clones (left panel) or KO clones for the long isoforms of TCF1 and LEF1 (DLKO1 and DLKO2) (right panel) in RPMI8402 cells. Cytoplasmic and nuclear fractions are analyzed and tubulin and H3 used as loading controls.

Source data are available online for this figure.

 © 2023 The Authors

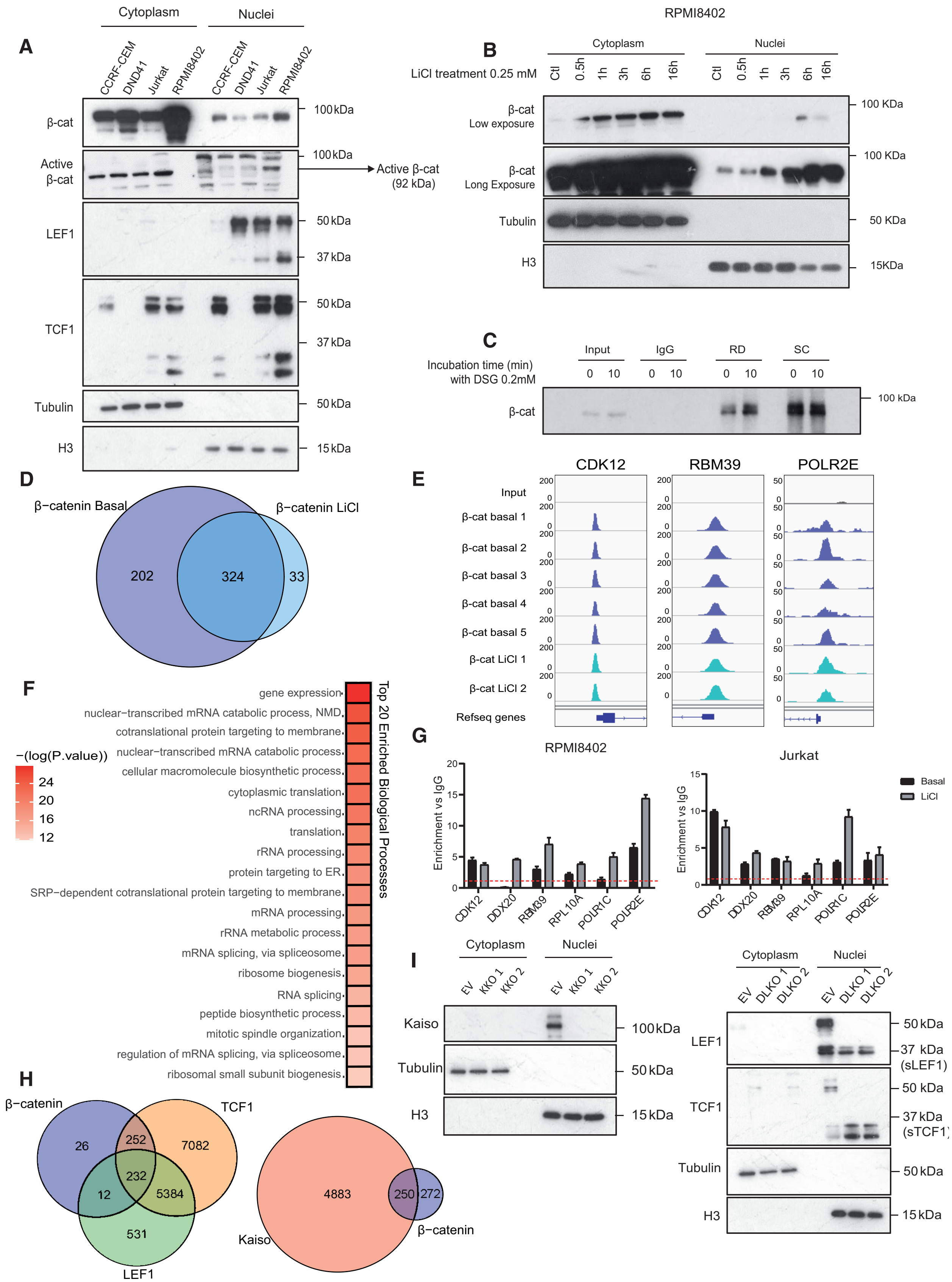
A
Cytoplasm
Nuclei
CCRF-CEM
DND41
Jurkat
RPMI8402
β-cat
Active β-cat
LEF1
TCF1
Tubulin
H3
100 kDa
50 kDa
37 kDa
15 kDa
Active β-cat (92 kDa)
B
RPMI8402
LiCl treatment 0.25 mM
Ctl
0.5h
1h
3h
6h
16h
β-cat Low exposure
β-cat Long Exposure
100 KDa
50 KDa
15KDa
C
Incubation time (min) with DSG 0.2mM
Input
IgG
RD
SC
100 kDa
D
β-catenin Basal
β-catenin LiCl
202
324
33
E
CDK12
RBM39
POLR2E
Input
β-cat basal 1
β-cat basal 2
β-cat basal 3
β-cat basal 4
β-cat basal 5
β-cat LiCl 1
β-cat LiCl 2
Refseq genes
F
−(log(P.value))
Top 20 Enriched Biological Processes
gene expression
nuclear−transcribed mRNA catabolic process, NMD
cotranslational protein targeting to membrane
nuclear−transcribed mRNA catabolic process
cellular macromolecule biosynthetic process
cytoplasmic translation
ncRNA processing
translation
rRNA processing
protein targeting to ER
SRP−dependent cotranslational protein targeting to membrane
mRNA processing
rRNA metabolic process
mRNA splicing, via spliceosome
ribosome biogenesis
RNA splicing
peptide biosynthetic process
mitotic spindle organization
regulation of mRNA splicing, via spliceosome
ribosomal small subunit biogenesis
G
RPMI8402
Jurkat
Enrichment vs IgG
Basal
LiCl
CDK12
DDX20
RBM39
RPL10A
POLR1C
POLR2E
H
β−catenin
TCF1
LEF1
26
252
7082
232
12
5384
531
4883
250
272
Kaiso
I
EV
KKO 1
KKO 2
Kaiso
DLKO 1
DLKO 2
37 kDa (sLEF1)
37 kDa (sTCF1)

Figure EV1.

Figure EV2. β-catenin-target gene expression in T-ALL cell lines.

A Representative heatmap of H3Ac enriched TSS-centered peaks in RPMI8402 cells in basal conditions and treated with LiCl 25 mM for 16 h (left panels) or H3K27Ac, H3K4me3 and H3K27me3 in RPMI8402, Jurkat or DND41 cell lines in basal conditions (right panels). Upper panels show β-catenin-binding targets and bottom panels show random genomic regions. Upper linear graphs show enrichment of the H3Ac mark in the TSS in the β-catenin-binding genes (blue) and in the random genomic regions (green).

B WB analysis of β-catenin levels in the cytoplasm, nucleoplasm and chromatin after treatment of RPMI8402 (left) or Jurkat cells (right) with β-catenin inhibitors ICG-001 or FH535 for 16 h at the indicated concentrations. Tubulin and H3 were used as loading controls.

C qPCR analysis of mRNA expression of β-catenin target genes in the RPMI8402 cell line after treatment with β-catenin inhibitor FH535 (30 μM, overnight). Graph represents the mean and SD of three independent experiments. Statistical significance was determined by two-sided Student's *t*-test.

D, E Flow cytometry cell cycle analysis of RPMI8402 cells after β-catenin inhibition (D) or knockdown (E). Top panels show representative flow cytometry density plots of cell cycle status. Bottom panels show the quantification of cell cycle phases distribution determined by DAPI and Ki67 incorporation in two independent experiments. Data represent the individual values per experiment and condition.

Source data are available online for this figure.

 © 2023 The Authors

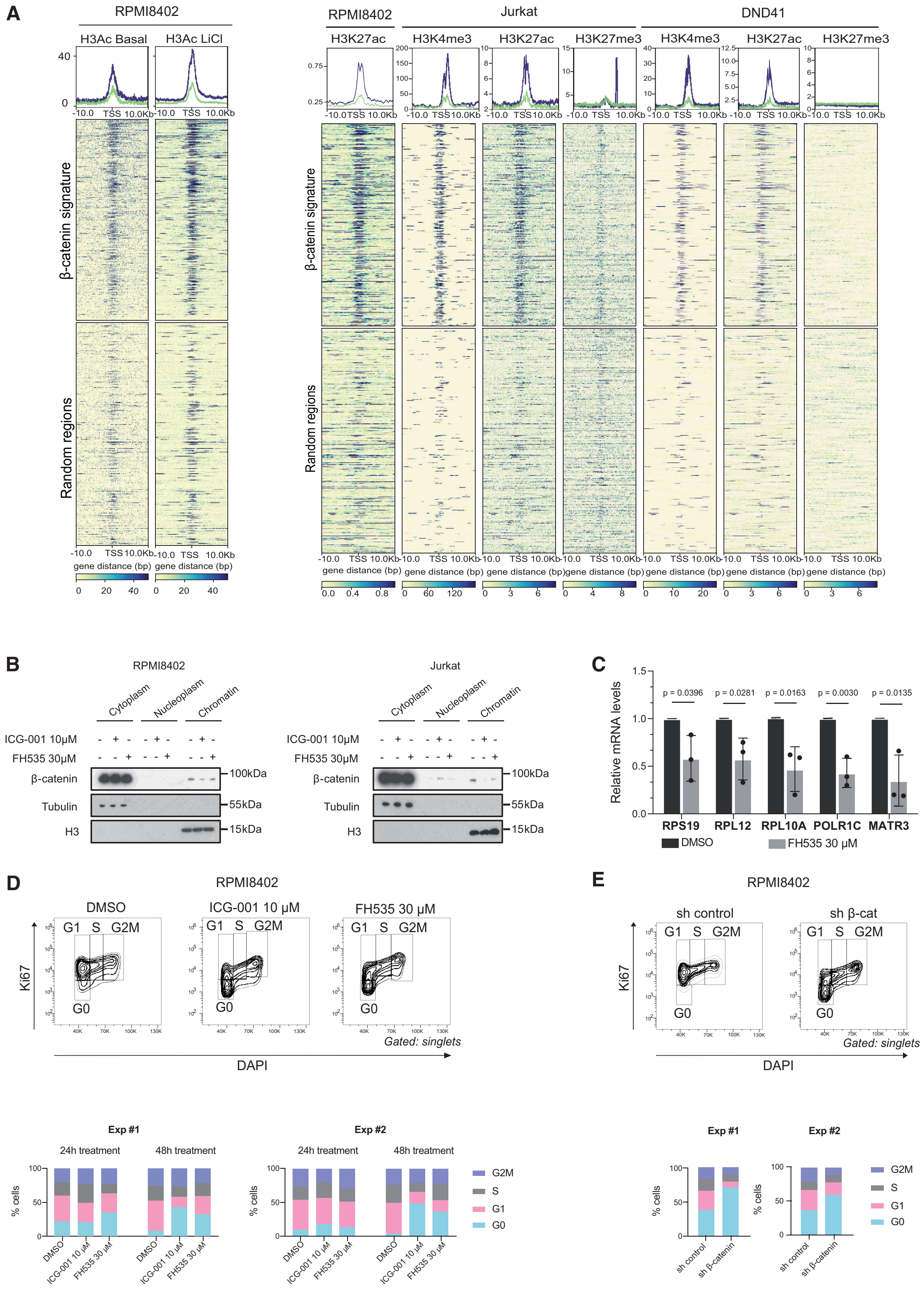
A
RPMI8402
H3Ac Basal
H3Ac LiCl
β-catenin signature
Random regions
gene distance (bp)
Jurkat
DND41
H3K27ac
H3K4me3
H3K27me3
B
RPMI8402
Jurkat
Cytoplasm
Nucleoplasm
Chromatin
ICG-001 10μM
FH535 30μM
β-catenin
Tubulin
H3
100kDa
55kDa
15kDa
C
Relative mRNA levels
p = 0.0396
p = 0.0281
p = 0.0163
p = 0.0030
p = 0.0135
RPS19
RPL12
RPL10A
POLR1C
MATR3
DMSO
FH535 30 μM
D
RPMI8402
DMSO
ICG-001 10 μM
FH535 30 μM
Ki67
DAPI
Gated: singlets
E
sh control
sh β-cat
Exp #1
Exp #2
24h treatment
48h treatment
% cells
G2M
S
G1
G0
sh β-catenin

Figure EV2.

Figure EV3. β-catenin-signature in T-ALL.

A Non-supervised hierarchical classification of T-ALL patients (GSE14618, samples with available survival data $N = 40$) according to the expression of the 156 β-catenin targets found differentially expressed (DEG) in the RNAseq from sh-β-catenin. T-ALL phenotype, patient outcome, vital status and age are depicted on the top of the heatmap. Genes are represented on the left side and patients are shown in the upper part. A summary of the most representative biological functions significantly enriched for each group of genes is shown on the left (Gene Ontology Biological functions significantly enriched (adjusted P-value < 0.05) in groups of genes GA ($N = 92$), GB ($N = 18$) and GC ($N = 38$)). ETP, Early T-cell precursor; ABD, absence of biallelic TCRgamma locus deletion.

B Kaplan–Meier curves representing disease free (DSF, left) and overall survival (OS, right) probability for groups of patients PA ($N = 8$), PB ($N = 8$) and PC ($N = 24$) obtained in (A). Time is represented in years. Statistical significance among groups was determined by log-rank test.

C Scaled β-catenin, ZBTB33/Kaiso, TCF1 and LEF1 expression in each patient cluster PA ($N = 8$), PB ($N = 8$) and PC ($N = 24$). Box and whiskers plot represents the median (central bar), Q1 and Q4 quartiles (low and high hinge, respectively) and minimum and maximum values (lower and higher whisker, respectively) of the indicated scaled expressions. Statistical significance among groups was determined by Kruskal–Wallis test. When significant, Kruskal–Wallis is followed by Wilcoxon test for pairwise comparisons.

D Scaled β-catenin, ZBTB33/Kaiso, TCF1 and LEF1 expression in refractory ($N = 6$), relapse ($N = 13$) and remission cases ($N = 21$). Box and whiskers plot represents the median (central bar), Q1 and Q4 quartiles (low and high hinge, respectively) and minimum and maximum values (lower and higher whisker, respectively) of the indicated scaled expressions. Statistical significance among groups was determined by Kruskal–Wallis test. When significant, Kruskal–Wallis is followed by Wilcoxon test for pairwise comparisons.

E DFS probability for patients based solely on β-catenin expression. In the left panel, the high β-catenin group comprises patients with a β-catenin expression higher than the median ($N = 20$ per group). In the right panel, groups are established based on scaled expression quartiles (high β-catenin, Q4 ($N = 10$); medium β-catenin, Q3 ($N = 10$); and low β-catenin, Q1 + Q2 ($N = 20$). Statistical significance among groups was determined by log-rank test.

F–H DFS probability for patients based solely on Kaiso (F), TCF1 (G), LEF1 (H) expression. High factor groups comprise patients with a factor expression higher than the median ($N = 20$ per group). Statistical significance among groups was determined by log-rank test.

© 2023 The Authors

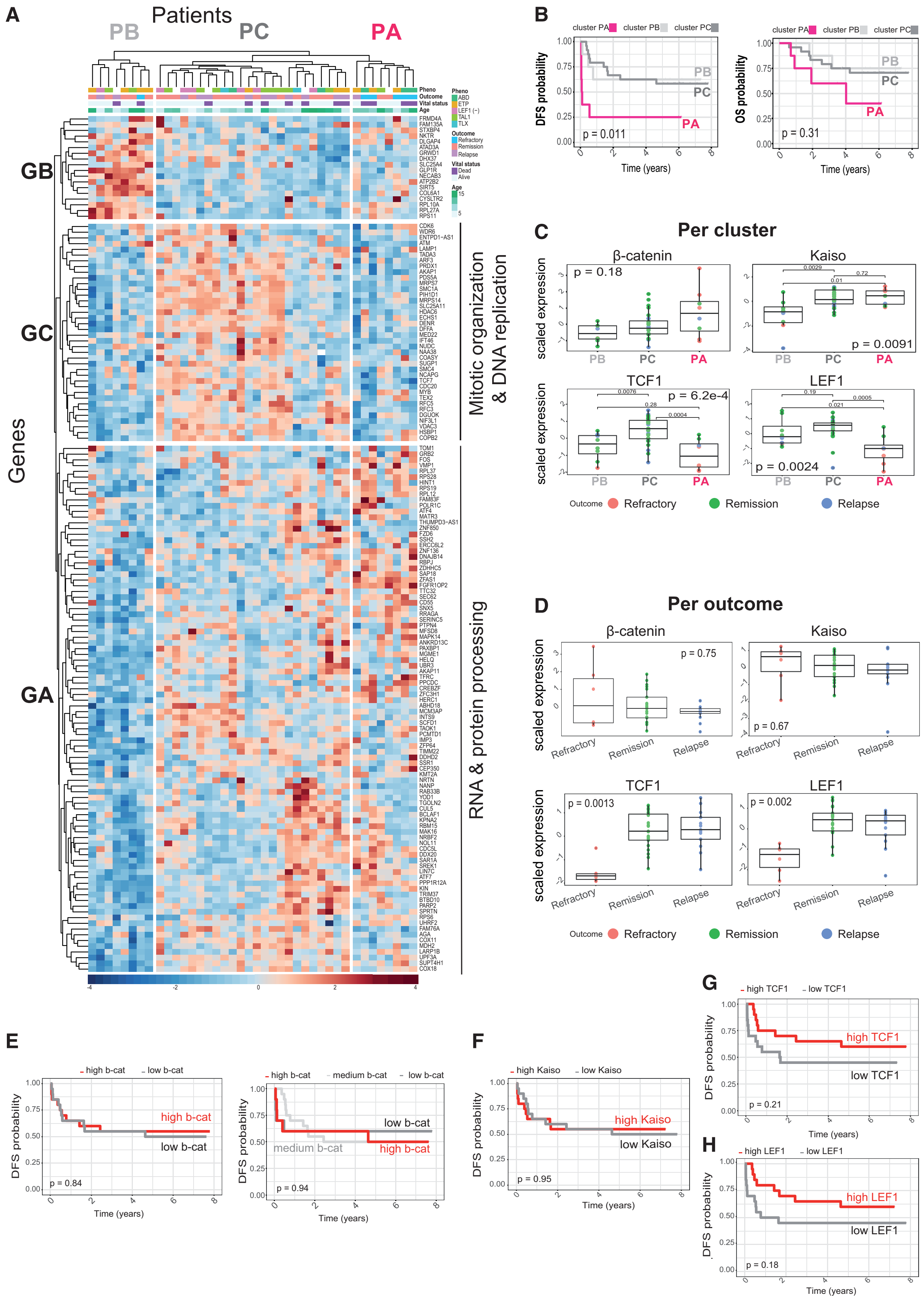
A
Patients
PB
PC
PA
GB
GC
GA
Genes
Mitotic organization & DNA replication
RNA & protein processing
B
DFS probability
OS probability
p = 0.011
p = 0.31
Time (years)
C
Per cluster
β-catenin
Kaiso
TCF1
LEF1
scaled expression
p = 0.18
p = 0.0091
p = 6.2e-4
p = 0.0024
Outcome Refractory Remission Relapse
D
Per outcome
p = 0.75
p = 0.67
p = 0.0013
p = 0.002
E
high b-cat
low b-cat
medium b-cat
p = 0.84
p = 0.94
F
high Kaiso
low Kaiso
p = 0.95
G
high TCF1
low TCF1
p = 0.21
H
high LEF1
low LEF1
p = 0.18

Figure EV3.

Figure EV4. Minimal β-catenin-signature in T-ALL.

A Kaplan–Meier curves representing DSF (left) and OS (right) probability for groups of patients from the discovery cohort clustered according to the expression of the 77 β-catenin targets differentially upregulated in the RNAseq from sh-β-catenin. Time is represented in years. Statistical significance among groups was determined by log-rank test.

B Summary of β-catenin targets differentially expressed between refractory ($N = 6$) and remission ($N = 21$) cases from GSE14618 (samples with available survival data) and summary of the top Gene Ontology Biological functions enriched in groups of β catenin targets upregulated ($N = 25$) or downregulated ($N = 17$; *P*-value < 0.05, Dataset EV3).

C Classification of T-ALL patients (GSE14618, samples with available survival data $N = 40$) according to the expression of the 42 β-catenin targets differentially expressed (*P*-value < 0.05) between refractory and remission patients. T-ALL phenotype, patient outcome, vital status and age are depicted on the top of the heatmap. Genes are represented on the left side and patients are shown in the upper part.

D β-catenin expression levels in patients divided per outcome (left panels) or cluster (right panels) from the subset of GSE14618 cohort with no survival data available (left), EGA cohort (middle) and TARGET cohort (right). Q1, Q2–Q3, and Q4 clusters established according to G1 ssGSEA quartiles from Fig 4B–D. Box and whiskers plot represents the median (central bar), Q1 and Q4 quartiles (low and high hinge, respectively) and minimum and maximum values (lower and higher whisker, respectively) of the indicated expression. Statistical significance among groups was determined by Kruskal–Wallis test. For GSE14614 cohort with no survival data (left panel), Q1 $N = 11$, Q2–Q3 $N = 20$, Q4 $N = 11$. For EGA cohort (middle panel), Q1 $N = 4$, Q2–Q3 $N = 14$, Q4 $N = 9$. For TARGET cohort (right panel), Q1 = 63, Q2–Q3 = 129, Q4 = 61 (D).

E, F Kaplan–Meier curves representing DFS (upper panel) and OS (lower panel) probability for group of patients included in Q4 from Fig 4, compared with the other patients (not Q4) from the discovery cohort (E) and TARGET cohort (F). Q4 $N = 11$ (in D) or $N = 61$ (in E), not Q4 $N = 31$ (in D) or $N = 192$ (in E). Statistical significance among groups was determined by log-rank test.

© 2023 The Authors

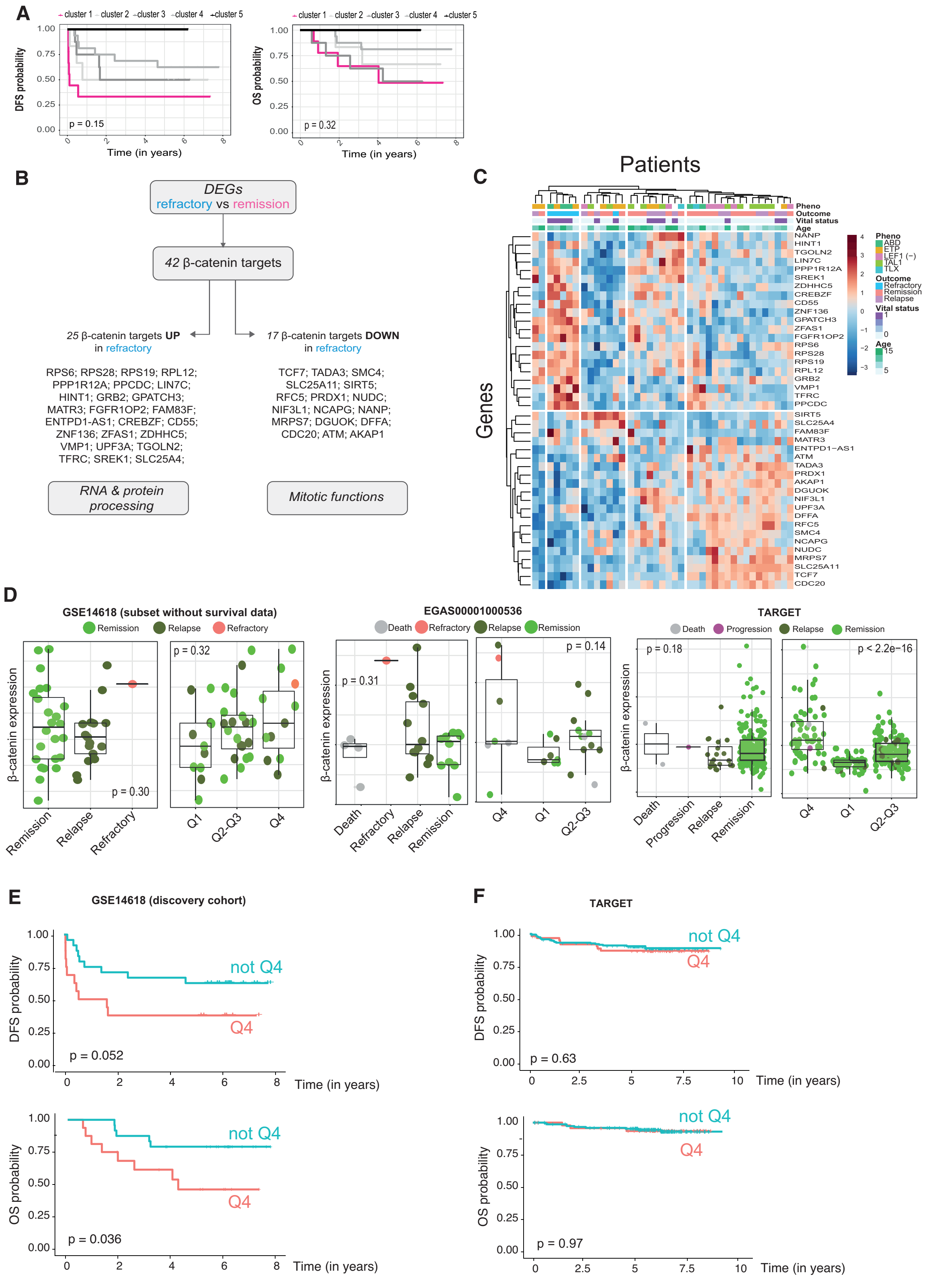
A
DFS probability
OS probability
p = 0.15
p = 0.32
Time (in years)
B
DEGs
refractory vs remission
42 β-catenin targets
25 β-catenin targets UP in refractory
17 β-catenin targets DOWN in refractory
RPS6; RPS28; RPS19; RPL12; PPP1R12A; PPCDC; LIN7C; HINT1; GRB2; GPATCH3; MATR3; FGFR1OP2; FAM83F; ENTPD1-AS1; CREBZF; CD55; ZNF136; ZFAS1; ZDHHC5; VMP1; UPF3A; TGOLN2; TFRC; SREK1; SLC25A4;
TCF7; TADA3; SMC4; SLC25A11; SIRT5; RFC5; PRDX1; NUDC; NIF3L1; NCAPG; NANP; MRPS7; DGUOK; DFFA; CDC20; ATM; AKAP1
RNA & protein processing
Mitotic functions
C
Patients
Genes
Pheno
Outcome
Vital status
Age
D
GSE14618 (subset without survival data)
EGAS00001000536
TARGET
β-catenin expression
p = 0.30
p = 0.32
p = 0.31
p = 0.14
p = 0.18
p < 2.2e−16
E
GSE14618 (discovery cohort)
not Q4
Q4
p = 0.052
p = 0.036
F
TARGET
p = 0.63
p = 0.97

Figure EV4.

Figure EV5. β-catenin effect in chemotherapy cell recovery.

A Effect of 48 h treatment with VCR on RPMI8402 cells upon β-catenin knockdown when compared with control cells. Significant differences: ****$P \leq 0.0001$, ***$P \leq 0.001$, **$P \leq 0.01$.

B Effect of 48 h single and combined treatments with VCR and ICG-001 on RPMI8402 cells viability. Significant differences: ****$P \leq 0.0001$, ***$P \leq 0.001$, **$P \leq 0.01$, *$P \leq 0.05$.

C Effect of VCR 48 h-treatment on cell viability of Jurkat (left) CCRF-CEM (central) or DND41 (right) cells in the presence or absence of increasing doses of the β-catenin inhibitor ICG-001. Dose–response curves represent the logistic fitting and individual points correspond to mean ± SD. ****$P \leq 0.0001$, ***$P \leq 0.001$, **$P \leq 0.01$, *$P \leq 0.05$ of IC50 comparison with respect to the chemotherapy single treatment.

D (left) Cell viability of RPMI8402 cells treated with MTX and during the recovery time in the presence or absence of ICG-001 after drug wash-out as fold change relative to day 0 ($n = 2$). (right) Cell viability of RPMI-8402 cells transduced with sh β-catenin or sh control treated with MTX and during the recovery time as fold change relative to day 0 ($n = 1$). **$P \leq 0.01$ with respect to control; #$P \leq 0.05$ with respect to VCR-treated cells. †† $P \leq 0.01$, † $P \leq 0.05$ with respect to recovery in complete medium.

E Percentage of viability of Jurkat (left), CCRF-CEM (central) and DND41 (right) cells after drug wash-out during recovery in the presence or absence of ICG-001 10 μM at the indicated time points. Significant differences: ****$P \leq 0.0001$, ***$P \leq 0.001$, **$P \leq 0.01$, *$P \leq 0.05$ with respect to recovery in complete medium.

Data information: For all applicable figure panels, data represent mean ± SD of three independent experiments, except in (D) (D left $n = 2$ independent experiments, and D right $n = 1$). Statistical significance was determined by one-way ANOVA with Tukey's correction for multiple comparison testing for (A and B) or by two-sided Student's *t*-test for (C–E).
Source data are available online for this figure.

© 2023 The Authors

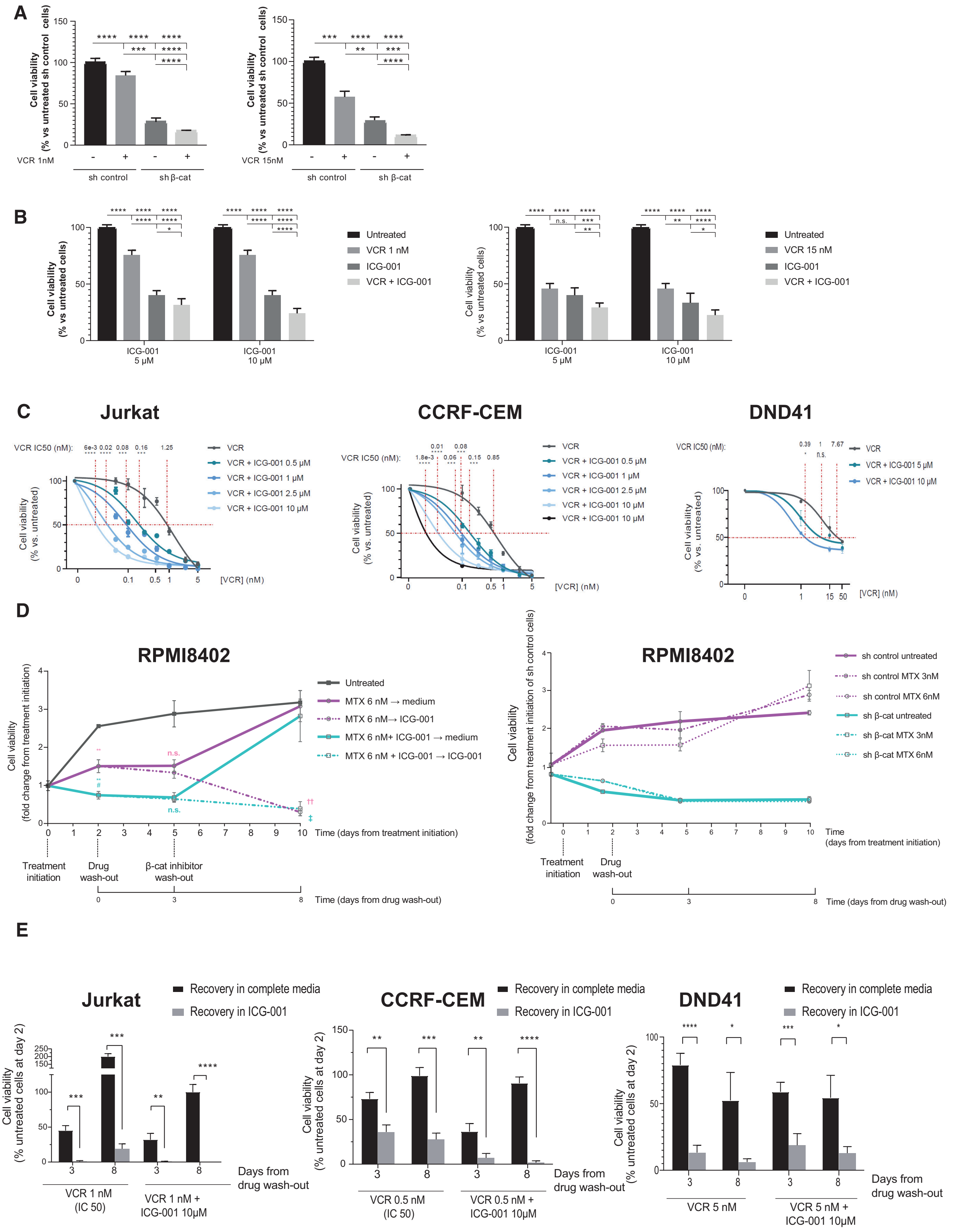

Figure EV5.
