# Supplementary material for: β‐Catenin activity induces an RNA biosynthesis program promoting therapy resistance in T‐cell acute lymphoblastic leukemia
Source: EMBO Mol Med. 2023 Jan 4;15(2):e16554. doi: 10.15252/emmm.202216554 (PMC9906382; doi:10.15252/emmm.202216554)
Supplement: Supplementary file 13 [file EMMM-15-e16554-s005.pdf]

EMBO Molecular Medicine

β-catenin activity induces an RNA biosynthesis program promoting therapy resistance in T-cell Acute Lymphoblastic Leukemia

Violeta García-Hernández, David Arambilet, Yolanda Guillen, Teresa Lobo, Maria Maqueda, Christos Gekas, Jessica González, Arnau Iglesias, Nerea Vega-García, Ines Sentís, Juan Trincado, Ian Márquez-López, Holger Heyn, Mireia Camós, Lluis Espinosa, and Anna Bigas
DOI: 10.15252/emmm.202216554

Corresponding author: Anna Bigas (abigas@imim.es)

Review Timeline:

Submission Date:	7th Jul 22
Editorial Decision:	15th Aug 22
Revision Received:	7th Nov 22
Editorial Decision:	5th Dec 22
Revision Received:	9th Dec 22
Accepted:	13th Dec 22

Editor: Lise Roth

Transaction Report:

(Note: With the exception of the correction of typographical or spelling errors that could be a source of ambiguity, letters and reports are not edited. Depending on transfer agreements, referee reports obtained elsewhere may or may not be included in this compilation. Referee reports are anonymous unless the Referee chooses to sign their reports.)

15th Aug 2022

Dear Dr. Bigas,

Thank you for the submission of your manuscript to EMBO Molecular Medicine, and please accept my apologies for the delay in getting back to you as it takes more time to secure referees and get their reports during the summer vacation season. We have now received feedback from the three reviewers who agreed to evaluate your manuscript. As you will see below, the reviewers raise substantial concerns on your work, which unfortunately preclude its publication in EMBO Molecular Medicine in its current form.

The reviewers find that the question addressed by the study is of potential interest, however they remain unconvinced that some of the major conclusions are sufficiently supported by the data. They thus raise the following major issues:
- unclear clustering strategies
- lack of animal models

If you feel you can satisfactorily address these points and those listed by the referees, you may wish to submit a revised version of your manuscript. Please attach a covering letter giving details of the way in which you have handled each of the points raised by the referees. A revised manuscript will once again be subject to review and we cannot guarantee at this stage that the eventual outcome will be favorable.

Revised manuscripts should be submitted within three months of a request for revision; they will otherwise be treated as new submissions, except under exceptional circumstances in which a short extension is obtained from the editor.

When submitting your revised manuscript, please carefully review the instructions that follow below. We perform an initial quality control of all revised manuscripts before re-review; failure to include requested items will delay the evaluation of your revision.

We require:

1) A .docx formatted version of the manuscript text (including legends for main figures, EV figures and tables). Please make sure that the changes are highlighted to be clearly visible.

2) Individual production quality figure files as .eps, .tif, .jpg (one file per figure). For guidance, download the 'Figure Guide PDF' (https://www.embopress.org/page/journal/17574684/authorguide#figureformat).

3) A .docx formatted letter INCLUDING the reviewers' reports and your detailed point-by-point responses to their comments. As part of the EMBO Press transparent editorial process, the point-by-point response is part of the Review Process File (RPF), which will be published alongside your paper.

4) A complete author checklist, which you can download from our author guidelines (https://www.embopress.org/page/journal/17574684/authorguide#submissionofrevisions). Please insert information in the checklist that is also reflected in the manuscript. The completed author checklist will also be part of the RPF.

5) It is mandatory to include a 'Data Availability' section after the Materials and Methods. Before submitting your revision, primary datasets produced in this study need to be deposited in an appropriate public database, and the accession numbers and database listed under 'Data Availability'. Please remember to provide a reviewer password if the datasets are not yet public (see https://www.embopress.org/page/journal/17574684/authorguide#dataavailability).
In case you have no data that requires deposition in a public database, please state so in this section. Note that the Data Availability Section is restricted to new primary data that are part of this study.

6) For data quantification: please specify the name of the statistical test used to generate error bars and P values, the number (n) of independent experiments (specify technical or biological replicates) underlying each data point and the test used to calculate p-values in each figure legend. The figure legends should contain a basic description of n, P and the test applied. Graphs must include a description of the bars and the error bars (s.d., s.e.m.). Please provide exact p values.

7) We would also encourage you to include the source data for figure panels that show essential data. Numerical data should be provided as individual .xls or .csv files (including a tab describing the data). For blots or microscopy, uncropped images should be submitted (using a zip archive if multiple images need to be supplied for one panel). Additional information on source data and instruction on how to label the files are available at
.

8) Our journal encourages inclusion of *data citations in the reference list* to directly cite datasets that were re-used and

obtained from public databases. Data citations in the article text are distinct from normal bibliographical citations and should directly link to the database records from which the data can be accessed. In the main text, data citations are formatted as follows: "Data ref: Smith et al, 2001" or "Data ref: NCBI Sequence Read Archive PRJNA342805, 2017". In the Reference list, data citations must be labeled with "[DATASET]". A data reference must provide the database name, accession number/identifiers and a resolvable link to the landing page from which the data can be accessed at the end of the reference. Further instructions are available at .

9) We replaced Supplementary Information with Expanded View (EV) Figures and Tables that are collapsible/expandable online. A maximum of 5 EV Figures can be typeset. EV Figures should be cited as 'Figure EV1, Figure EV2" etc... in the text and their respective legends should be included in the main text after the legends of regular figures.
- For the figures that you do NOT wish to display as Expanded View figures, they should be bundled together with their legends in a single PDF file called *Appendix*, which should start with a short Table of Content. Appendix figures should be referred to in the main text as: "Appendix Figure S1, Appendix Figure S2" etc.
- Additional Tables/Datasets should be labeled and referred to as Table EV1, Dataset EV1, etc. Legends have to be provided in a separate tab in case of .xls files. Alternatively, the legend can be supplied as a separate text file (README) and zipped together with the Table/Dataset file.
See detailed instructions here:
.

10) The paper explained: EMBO Molecular Medicine articles are accompanied by a summary of the articles to emphasize the major findings in the paper and their medical implications for the non-specialist reader. Please provide a draft summary of your article highlighting
- the medical issue you are addressing,
- the results obtained and
- their clinical impact.
This may be edited to ensure that readers understand the significance and context of the research. Please refer to any of our published articles for an example.

11) For more information: There is space at the end of each article to list relevant web links for further consultation by our readers. Could you identify some relevant ones and provide such information as well? Some examples are patient associations, relevant databases, OMIM/proteins/genes links, author's websites, etc...

12) Conflict of interest: We updated our journal's competing interests policy in January 2022 and request authors to consider both actual and perceived competing interests. Please review the policy https://www.embopress.org/competing-interests and update your competing interests if necessary.

13) Every published paper now includes a 'Synopsis' to further enhance discoverability. Synopses are displayed on the journal webpage and are freely accessible to all readers. They include a short stand first (maximum of 300 characters, including space) as well as 2-5 one-sentences bullet points that summarizes the paper. Please write the bullet points to summarize the key NEW findings. They should be designed to be complementary to the abstract - i.e. not repeat the same text. We encourage inclusion of key acronyms and quantitative information (maximum of 30 words / bullet point). Please use the passive voice. Please attach these in a separate file or send them by email, we will incorporate them accordingly.

Please also suggest a striking image or visual abstract to illustrate your article as a PNG file 550 px wide x 300-600 px high.

14) As part of the EMBO Publications transparent editorial process initiative (see our Editorial at http://embomolmed.embopress.org/content/2/9/329), EMBO Molecular Medicine will publish online a Review Process File (RPF) to accompany accepted manuscripts.
In the event of acceptance, this file will be published in conjunction with your paper and will include the anonymous referee reports, your point-by-point response and all pertinent correspondence relating to the manuscript. Let us know whether you agree with the publication of the RPF and as here, if you want to remove or not any figures from it prior to publication.
Please note that the Authors checklist will be published at the end of the RPF.

EMBO Molecular Medicine has a "scooping protection" policy, whereby similar findings that are published by others during review or revision are not a criterion for rejection. Should you decide to submit a revised version, I do ask that you get in touch after three months if you have not completed it, to update us on the status.

I look forward to receiving your revised manuscript.

Yours sincerely,

Lise Roth

Lise Roth, PhD
Senior Editor
EMBO Molecular Medicine

***** Reviewer's comments *****

Referee #1 (Comments on Novelty/Model System for Author):

If animal model could be used to verify the finding that will make the conclusion much stronger

Referee #1 (Remarks for Author):

In this manuscript, the authors studied the function of β-catenin in promoting therapy resistance in T acute lymphoblastic leukemia.
BY performing ChIP-seq in 2 cell lines, they showed a correlation between β-catenin and ZBTB33/Kaiso. Then through RNAi knocking-down β-catenin in RPMI8402 cells, authors found that a down-regulation of cell cycle related genes and an up-regulation of RNA processing genes. RNA synthesis down-regulation was confirmed by incorporation of O-propargyl-puromycin and EU. By analyzing patients' sample and utilizing single-sample gene set enrichment analysis, authors claim that refractory patients and poor diagnosis is correlated with β-catenin-dependent RNA processing. Lastly, they combined inhibition of β-catenin with chemotherapy compound and found a decreased RPMI8402 cell viability. However, there are some flaws in this manuscript.

Major:

1. In Figure 2C. Upregulated genes are cycle related while multiple reports revealed the promoting effect of β-catenin on cell cycle. How to explain this data?
2. Within the study of GSE14618, there are 92 patients with T-ALL treated-on Children's Oncology Group studies 8704 (42 patients) and 9404 (50 patients). Any reason why cog 9404 is used when 8704 is not? Moreover, COG Study 9404 has 50 patients and only 40 samples are examined here. What's the standard/reason to exclude the other 12 patients?
3. P3...Analysis of the mean expression levels of β-catenin, TCF1, LEF1 and ZBTB33/Kaiso for each cluster of patients indicated that those patients belonging to the worse prognostic cluster (PA)...
How do expressions of β-catenin, TCF1, LEF1 and ZBTB33/Kaiso indicate the patients prognostic cluster? The logic is not clear, and conclusion lacks sufficient data and explanation.
4. P4...Genes in the G1 module are consistently upregulated in the refractory patients, and this is significantly associated with G2 downregulation in the discovery cohort...
The evidence provided is weak.
5. No animal T-All model was tested. By knocking down β-catenin, many proliferation-related signaling pathways and oncogenes will be restrained, which has been reported by multiple publications. This section lacks connection to the RNA processing.
6. Figure S2d has neither figure legend nor mention in the content.
Minor:
1. Fig. S1C. DSG incubation time and concentration is different compared to M&M.
2. All Supplementary Figures have major layout and alignment issue.
3. Figure 1. Crispr Knock-out clones could be biased.
4. P3... refractory outcome (Fig S3d) tend to have higher expression levels of β-catenin and Kaiso and significantly lower levels of TCF1 and LEF1...
Expression levels of β-catenin and Kaiso are almost the same throughout all three groups (p=0.75/0.67).
5. P4...OS, Abbreviation without previous explanation.
6. Figure 5A. B and C. Starting point of control group is not 100%
7. Figure S4. There is 2 "?" at the end.

Referee #2 (Comments on Novelty/Model System for Author):

-cell lines model appropriate; it lacks some experimental data using patients samples.

Referee #2 (Remarks for Author):

In this manuscript from Prof. Bigas' lab the authors used a variety of experiments and analyses to demonstrate the importance of b-catenin and its downstream target pathways in T-ALL. In general, the data shown is well done and it does offer some novel aspects in T-ALL physiopathology: e.g. the molecular data demonstrating the importance of b-catenin pathway and the in vitro cell line results (Fig. 5). However, the way the data was/is presented in relation to the chemoresistance there is lack of direct experimental demonstration of this with b-catenin associated molecular signatures, albeit the authors showed a good correlation with the molecular signature and the OS in one patient cohort study. There are few aspects/questions that should be addressed to further improve this work:

1-The in vitro data (Fig. 5) does support the functional importance of b-catenin in the RPMI-8402 cell line but since the data before this was/is to show the correlation between the molecular signatures vs chemoresistance and this cell line is not chemoresistant to VCR hence, it seems misplaced. This reviewer suggests to move this data as Fig. 3 after the molecular data as proof of principle/supporting the functional importance of b-catenin in T-ALL. Importantly, in vivo work is also required to further support this (some NSG mice transplanted with this cell line then treating mice with the different conditions e.g. VCR, ICG-001 etc, as like in vitro in Fig. 5e). Altogether these results will support the general importance of b-catenin in T-ALL.

2-Following the data above, then focusing on the importance of the molecular signature with the clinical outcome it will read better. However, in this part it lacks a direct functional demonstration of the molecular signature and sensitivity/chemoresistance of chemotherapy using patients samples in vitro. The authors should test using patients samples in vitro (e.g. on MS5-DL1) and test the in vitro sensitivity of the cells derived from remission vs refractory samples from the different groups (P1, P2, etc from COG study 9404) and also whether inhibiting b-catenin (ICG-001) will improve responses. This reviewer understands that sometimes is not possible to test all the samples due to lack of material however, some from Q1 and Q4 including few refractory ones will help to consolidate these results.

3-Is a bit confusing to follow all the different clustering and its use for the DFS studies. In the COG study 9404 (with survival data) it was used P1, P2, etc and the TARGET with 3 clusters based on Q1-Q4. Can the authors show either or both in COG study 9404 (with survival data) using Q1-Q4 to plot DSF and/or the TARGET with similar clustering as P1 to P5 in the COG study 9404 study? Maybe when using Q1-Q4 clustering is not predictive even in the COG study 9404 (with survival data) cohort? If so, it takes away the power of this G1/G2 ssGSEA score analysis. This reviewer questions the importance of this analysis as even patients located in Q4 (high in G1 score) can be in remission and those that responded but then relapsed (seen more clearly in the TARGET cohort) which means using this signature does not necessary can predict that a patient can be refractory of the treatment used. Also is unclear how the P1 to P5 clustering was originated, can the authors clarify?

4-In the TARGET cohort, there was/is no differences in DFS between the different clusters (Fig. 4d and e) potentially due to the lack of refractory samples in cluster A that are able to augment this difference seen in the COG study 9404 (P1 cluster vs the rest). Or maybe the use of this clustering based on G1/G2 ssGSEA score is not powerful enough? If using a different clustering (as requested above) even with similar results are found perhaps is better to move as supplementary as there is insufficient refractory samples in this cohort hence the data is not that strong.

Minor:
-supplementary figures need attention, as after pdf conversion many graphs were distorted/misplaced hence it was difficult to analyse the data.

Referee #3 (Remarks for Author):

This is an interesting study characterizing beta-catenin functions in resistance to therapy with a focus on RNA binding-related genes as critical targets of the catenin signaling pathway. As resistance to therapy is an unmet need in T-ALL, the focus of the study is of interest for scientists and physicians alike. Nevertheless, a few parts of the current version of the study need to be better connected, especially with regards to the role of the identified catenin targets. Specific comments and questions are shown below:

Is the phenomenon described a general T-ALL phenomenon or it applies to a specific subtype?

The authors showed that catenin affects the levels of RNA binding proteins, such as RBM39. Please discuss the roles of those proteins and their impact on the transcriptome. Can silencing of any of those proteins recapitulate the molecular effect in the transcriptome caused by b-catenin silencing?

To showcase the importance of the downstream targets of catenin pathway, the authors need to rescue the phenotype of b-catenin silencing via expressing at least one of the downstream targets.

Ideally the authors need to show protein levels of the RBPs, as it is well known that factors implicated in RNA metabolism can be

regulated at the post-transcriptional level (via cross-talk of their activities, nonsense mediated decay etc).

Overall, T-ALL transcription factors (TFs) co-bind genomic targets (see Sanda et al., Cancer Cell, 2012 as an example). Are the loci in question co-bound by other leukemia TFs, such as NOTCH1, or TAL1? This could mean that targeting those proteins might affect the targets in question. The authors can use published ChIP-Seq data to answer this. Can modulation of those TFs lead to the same effect as catenin silencing? The authors can also analyze publicly available data to answer this.

The reviewers find that the question addressed by the study is of potential interest, however they remain unconvinced that some of the major conclusions are sufficiently supported by the data. They thus raise the following major issues:
● unclear clustering strategies
● lack of animal models

Answer:
We have performed new experiments and analyses to address both issues.
We have tested alternative clustering strategies and clarified the strategy chosen in the methodology to address the first issue.
We have used the NSG mice model transplanted with RPMI8402 T-ALL cells to test the effect of β-catenin inhibition in the response to chemotherapy treatment as suggested by reviewer 2. We compared the leukemic burden persisting in mice treated with chemotherapy plus/minus IGC-001 β-catenin inhibitor. We now show in the new Figure 6 the reduced level of leukemic cells in PB and BM and the abrogation of infiltration in the liver and spleen of mice treated with the β-catenin inhibitor.

Referee #1 (Comments on Novelty/Model System for Author):
If animal model could be used to verify the finding that will make the conclusion much stronger
Answer: We have now added the animal model suggested by the reviewer 2. See below.

Referee #1 (Remarks for Author):
Major:
1. In Figure 2C. Upregulated genes are cycle related while multiple reports revealed the promoting effect of β-catenin on cell cycle. How to explain this data?
Answer: We agree that we need to clarify that the enrichment of cell cycle related categories refers exclusively to mitotic organization, which does not necessarily mean that cells progress into cell cycle. It has been previously shown that β-catenin and Wnt members interact with different proteins in the centrosomes during mitosis having an impact in spindle formation or chromatid segregation (reviewed in Mbom et al, Bioessays 2013). All the upregulated genes belong to these specific functions and the vast majority are not direct β-catenin targets (by ChIP-seq), consequently we hypothesize that indirect feedback loops affect the expression of these genes in the absence of β-catenin.
However, to experimentally validate the effect of β-catenin deletion in T-ALL, we have performed cell cycle analysis of RPMI8402 in β-catenin-inhibited conditions. We found that cell cycle progression is repressed as expected upon β-catenin depletion. We have now included the analysis in FigS 2d and e). We have now included this explanation in the text, and references to 'cell cycle' in the text have been refined to allude to mitotic functions.

In the text:
'Since genes statistically up-regulated after β-catenin knockdown are associated to mitotic processes, we performed cell cycle analysis of RPMI8402 cells to test the effect of β-catenin inhibition (Fig S2d and S2e). Consistent with previous studies (O Tetsu and McCormick, Nature 2999), we found that cell cycle progression is repressed in β-catenin-inhibited cells (Fig S2d, S3e). Thus, enrichment of cell cycle related categories refers exclusively to mitotic organization, but does not result in cell cycle progression'

2. Within the study of GSE14618, there are 92 patients with T-ALL treated-on Children's Oncology Group studies 8704 (42 patients) and 9404 (50 patients). Any reason why cog

9404 is used when 8704 is not? Moreover, COG Study 9404 has 50 patients and only 40 samples are examined here. What's the standard/reason to exclude the other 12 patients?
Answer: In order to identify signatures with prognosis value, we only used samples from patients with clinical survival data available, which are the 40 of 50 samples from the 9404 series. The 42 patients from the 8704 study do not have survival data, which precluded including them in the analysis. This has been now specified in the methods:

'The transcriptomes of T-ALL patients were from GEO (GSE14618, including COG study 9404 and COG study 8704), EGA (EGAS00001000536) and TARGET (https://ocg.cancer.gov/programs/target) repositories. The transcriptomes of primary samples from GSE14618 obtained by microarrays were analyzed with the affy and limma R package. Data from COG studies 9404 (50 patients) and 8704 (42 patients) were processed independently. Only 40 out of the 50 primary samples from COG study 9404 had available survival clinical data and were further used as a discovery cohort for the identification of predictive signatures. The discovery cohort included 21 complete remission, 13 relapses and 6 refractory patients and were classified into the phenotypes ETP (Early T-cell precursor), ABD (absence of biallelic TCRgamma locus deletion), LEF1 negative, TAL1, and TLX1. The corresponding clinical data was provided by Alejandro Gutiérrez (Dana Faber, USA). COG 8707 contained 42 patients with no survival data and was comprised by 24 remission, 17 relapsed and 1 refractory patient.'

3. P3...Analysis of the mean expression levels of β-catenin, TCF1, LEF1 and ZBTB33/Kaiso for each cluster of patients indicated that those patients belonging to the worse prognostic cluster (PA)...How do expressions of β-catenin, TCF1, LEF1 and ZBTB33/Kaiso indicate the patients prognostic cluster? The logic is not clear, and conclusion lacks sufficient data and explanation.
Answer: We have rephrased the sentence to clarify that the worst prognostic cluster (PA), which includes 5 out of the 6 refractory patients, show higher (but not significant) median expression levels of β-catenin and Kaiso and significant lower levels of TCF1 and LEF1 when compared to the other two clusters (PB and PC). However, as indicated in the manuscript, no significant differences were found in patient outcome (Kaplan-Meier curves) when considering the sole expression of none of these factors (Fig. S3 from e to h).

Thus, this sentence has been reviewed in the manuscript as follows:

'The median expression levels of β-catenin and ZBTB33/Kaiso were slightly higher in the worst prognostic cluster PA than in the other two clusters (PB and PC). However, when considering TCF1 and LEF1, PA cluster showed significantly lower levels than the rest (Fig S3c). A similar behavior was observed for TCF1 and LEF1 in the refractory patients when compared to relapse and remission samples, while β-catenin and ZBTB33/Kaiso were not different (Fig S3d).'

4. P4...Genes in the G1 module are consistently upregulated in the refractory patients, and this is significantly associated with G2 downregulation in the discovery cohort... The evidence provided is weak.
Answer: As mentioned in the previous section of the paper ('*β-catenin transcriptional target gene signature identified T-ALL refractory patients with worst outcome*', page 4): 'In the poor prognosis P1 cluster of patients, the G1 module was primarily upregulated and included most of the genes involved in RNA processing functions. In contrast, G2 genes (enriched for DNA repair functions) were downregulated'. Following this reasoning, in figure

4 we evaluated the enrichment prolife of G1 and G2 gene sets in the individual samples of additional cohorts.
The significant association between G1 and G2 modules is assessed in figure 4a based on the correlation between ssGSEA scores. As shown in Fig. 4 there is a significant negative correlation between G1 and G2 modules in all tested cohorts, with refractory patients showing the highest expression of G1 genes.
Thanks to the reviewer's comment, we have realized that this sentence is confusing and misplaced, and therefore it has now been removed from the manuscript.

5. No animal T-All model was tested.
Answer: We have now included the NSG mice model transplanted with RPMI8402 T-ALL cells to test the effect of β-catenin inhibition in the response to chemotherapy treatment as suggested by reviewer2. We compared the leukemic burden persisting in mice treated with chemotherapy plus/minus IGC-001 β-catenin inhibitor. We now show in the new Figure 6 that treatment with β-catenin inhibitor plus chemotherapy imposed a reduction of the leukemic burden in PB and BM, and strikingly reduced tumor infiltration in liver and spleen of mice (when compared with chemotherapy alone).

In the text:
'To test the efficacy of ICG-001 in chemotherapy response in vivo, we transplanted 5x10^5 RPMI8402 T-ALL cells into sublethally irradiated NSG mice and were treated with a chemotherapy cocktail (Vincristine, Dexamethasone and L-Asparaginase) starting three days later. The treated animals were divided in 2 groups receiving DMSO vehicle ('Chemo+DMSO', n=4) or ICG-001 β-catenin inhibitor ('Chemo+ICG-001', n=4) respectively(Fig 6a). 2 more mice were left untreated and used as controls to monitor leukemic evolution (Fig 6a). 20 days after treatment initiation, one control and one 'Chemo+DMSO' mice reached a humane endpoint and were sacrificed. We compared the leukemic burden persisting in mice after 3 weeks of treatment initiation (when first animals had to be sacrificed) and found that treatment with β-catenin inhibitor plus chemotherapy imposed a reduction of the leukemic burden in PB (Fig 6b and 6c) and BM, and strikingly reduced the number of T-ALL cells in the liver and spleen (compared with chemotherapy alone) as detected by flow cytometry (Fig 6c). Moreover, liver visual inspection showed an almost abrogation of tumor nodules (Fig 6d) that was confirmed by H&E staining (Fig 6g) and quantified (Fig 6h). Liver and spleen relative weight was also significantly decreased in the ICG-001—treated animals (Fig 6e and f).'

By knocking down β-catenin, many proliferation-related signaling pathways and oncogenes will be restrained, which has been reported by multiple publications. This section lacks connection to the RNA processing.
Answer: We agree that β-catenin affects different proliferation-related pathways in different cell types and we also found cell cycle inhibition in the absence of β-catenin in T-ALL cells (new Fig S2). However, most likely metabolic adaptations, including RNA processing and protein biogenesis, are directly linked to the capacity of cells to proliferate.
If the reviewer is referring to the possible connection between the RNA processing function and chemotherapy resistance in T-ALL, we have now tested the expression levels of several of these β-catenin-dependent genes after chemotherapy, in the presence or absence of the β-catenin inhibitor. We detected decreased expression upon β-catenin abrogation of most selected targets related to RNA and protein biosynthesis (RPL10A, RPS19, RPL12 or POLR1C) in pairwise comparisons.

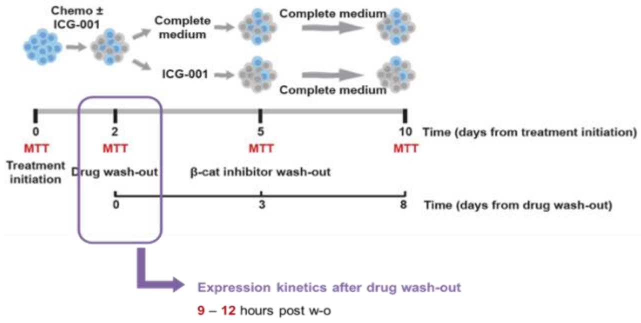

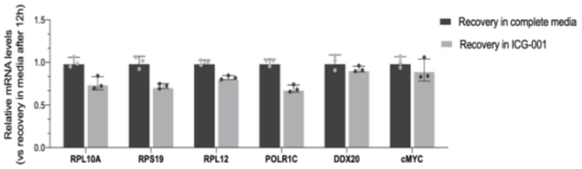

Fig.1 qPCR analysis of the indicated genes in the RPMI8402 cell line 12h after chemotherapy wash-out during recovery in the presence or absence of ICG-001 (10 µM). Relative expression of each gene is normalized to the recovery without ICG-001 condition. A representative experiment is shown.

6. Figure S2d has neither figure legend nor mention in the content.

Answer: The reviewer is right. Panel S2d was mistakenly added to the figure. We apologize for the mistake, we have now removed this panel from the figure, which in turn has been updated to include the cell cycle analysis referred to in the first question.

Minor:

1. Fig. S1C. DSG incubation time and concentration is different compared to M&M.

Answer: We have revised it (0.2mM and 10 min).

2. All Supplementary Figures have major layout and alignment issue.

Answer: We have realigned the suppl. Figures.

3. Figure 1. Crispr Knock-out clones could be biased.

Answer: it was difficult to obtain stable KO clones for all these elements, but we tested several clones for TCF1 and LEF1 double KO for the expression of β-catenin target genes and the results were consistent. We also tested single TCF1 KO and the results were similar to the ones reported for the double KO, but not so regular. It was harder to obtain Kaiso KO and there was more variability in the results as can be seen in the 2 clones represented in Figure 1. For this reason, we confirmed results and performed additional experiments by knocking down β-catenin and Kaiso (Ex. Fig 2).

4. P3... refractory outcome (Fig S3d) tend to have higher expression levels of β-catenin and Kaiso and significantly lower levels of TCF1 and LEF1...

Expression levels of β-catenin and Kaiso are almost the same throughout all three groups (p=0.75/0.67).

Answer: The reviewer is right. There are no statistically significant differences in the level of β-catenin and Kaiso expression between different groups as shown in Fig S3. Significant differences are only obtained for TCF1 and LEF1. The complete sentence has been reviewed in the manuscript as follows:

'The median expression levels of β-catenin and ZBTB33/Kaiso were slightly higher in the worst prognostic cluster PA than in the other two clusters (PB and PC). However, when considering TCF1 and LEF1, PA cluster showed significantly lower levels than the rest (Fig S3c). A similar behavior was observed for TCF1 and LEF1 in the refractory patients when compared to relapse and remission samples, while β-catenin and ZBTB33/Kaiso were not different (Fig S3d).'

5. P4...OS, Abbreviation without previous explanation.
Answer: We have added the explanation.

6. Figure 5A. B and C. Starting point of control group is not 100%
Answer: Figures 5a-c show both the non-linear regression (curves) and the mean values (dots) of dose-response experiments. This is indicated in the legend of Figure 5 ('Dose-response curves represent the logistic fitting and individual points correspond to mean ± SD of three independent experiments'). The dot corresponding to the untreated condition (starting point, [drug] = 0 in the x axis) is 100 %. However, it is the GraphPad Prism program who defines the best fitting curve that is not always starting at 100%. We have now further detailed the strategy used to normalize drug responses in the methods section.

'MTT absorbance of the cells incubated in the vehicle (untreated) was set to 100 % viability to calculate the dose-response curves. We used GraphPad Prism 8 to generate the logistic fitting curve by non-linear regression model and to calculate IC50 for each experiment. Treatments were performed in triplicate and repeated three times.'

7. Figure S4. There is 2 "?" at the end.
Answer: We have corrected the mistake.

Referee #2 (Comments on Novelty/Model System for Author):

-cell lines model appropriate; it lacks some experimental data using patient samples.
Answer: All patient data has been obtained from public available datasets and we do not have access to these patient samples. Instead we have performed in vivo experiments with the RPMI8402 cells as suggested by the reviewer.

Referee #2 (Remarks for Author):

In this manuscript from Prof. Bigas' lab the authors used a variety of experiments and analyses to demonstrate the importance of b-catenin and its downstream target pathways in T-ALL. In general, the data shown is well done and it does offer some novel aspects in T-ALL physiopathology: e.g. the molecular data demonstrating the importance of b-catenin pathway and the in vitro cell line results (Fig. 5).
Answer: We thank the reviewer for the supportive words.

However, the way the data was/is presented in relation to the chemoresistance there is lack of direct experimental demonstration of this with b-catenin associated molecular signatures, albeit the authors showed a good correlation with the molecular signature and the OS in one patient cohort study.

There are few aspects/questions that should be addressed to further improve this work:

1-The in vitro data (Fig. 5) does support the functional importance of b-catenin in the RPMI-8402 cell line but since the data before this was/is to show the correlation between the molecular signatures vs chemoresistance and this cell line is not chemoresistant to VCR hence, it seems misplaced. This reviewer suggests to move this data as Fig. 3 after the molecular data as proof of principle/supporting the functional importance of b-catenin in T-ALL.

Answer: We thank the reviewer for the constructive suggestions, however we believe that placing the chemotherapy sensitivity data to support the finding that β-catenin activity is important for the chemotherapy response in T-ALL patients is more relevant for the reader. About the idea that RPMI8402 cells are not resistant to VCR, this is not totally supported by the in vitro data showing increased sensitivity in combination with β-catenin inhibitors. Most important, we have now included new in vivo data showing that RPMI8402 can continue growing in the experimental group treated with chemotherapy alone (including VCR) similar to the untreated group, whereas this capacity is significantly reduced in the combination treatment with β-catenin inhibitors. We are convinced that new Fig 6 reinforces the usefulness of RPMI-8402 cells for studying chemotherapy resistance.

Importantly, in vivo work is also required to further support this (some NSG mice transplanted with this cell line then treating mice with the different conditions e.g. VCR, ICG-001 etc, as like in vitro in Fig. 5e). Altogether these results will support the general importance of b-catenin in T-ALL.
Answer: As the reviewer suggested, and as mentioned above, we have tested the efficacy of a chemotherapy cocktail compared to chemotherapy plus IGC-001 β-catenin inhibitor in NSG mice transplanted with RPMI8402. We now show in the new Figure 6 the reduction of leukemic cells in PB and BM and the almost complete abrogation of infiltration in liver and spleen of mice in the combination treatment. We thank the reviewer for this suggestion that strongly supports the general importance of β-catenin in T-ALL chemotherapy resistance.

In the text:
'To test the efficacy of ICG-001 in chemotherapy response in vivo, we transplanted $5x10^5$ RPMI8402 T-ALL cells into sublethally irradiated NSG mice and were treated with a chemotherapy cocktail (Vincristine, Dexamethasone and L-Asparaginase) starting three days later. The treated animals were divided in 2 groups receiving DMSO vehicle ('Chemo+DMSO', n=4) or ICG-001 β-catenin inhibitor ('Chemo+ICG-001', n=4) respectively(Fig 6a). 2 more mice were left untreated and used as controls to monitor leukemic evolution (Fig 6a). 20 days after treatment initiation, one control and one 'Chemo+DMSO' mice reached a humane endpoint and were sacrificed. We compared the leukemic burden persisting in mice after 3 weeks of treatment initiation (when first animals had to be sacrificed) and found that treatment with β-catenin inhibitor plus chemotherapy imposed a reduction of the leukemic burden in PB (Fig 6b and 6c) and BM, and strikingly reduced the number of T-ALL cells in the liver and spleen (compared with chemotherapy alone) as detected by flow cytometry (Fig 6c). Moreover, liver visual inspection showed an almost abrogation of tumor nodules (Fig 6d) that was confirmed by H&E staining (Fig 6g) and quantified (Fig 6h). Liver and spleen relative weight was also significantly decreased in the ICG-001—treated animals (Fig 6e and f).'

2-Following the data above, then focusing on the importance of the molecular signature with the clinical outcome it will read better. However, in this part it lacks a direct functional demonstration of the molecular signature and sensitivity/chemoresistance of chemotherapy using patients samples in vitro. The authors should test using patients samples in vitro (e.g. on MS5-DL1) and test the in vitro sensitivity of the cells derived from remission vs refractory samples from the different groups (P1, P2, etc from COG study 9404) and also whether inhibiting b-catenin (ICG-001) will improve responses.
This reviewer understands that sometimes is not possible to test all the samples due to lack of material however, some from Q1 and Q4 including few refractory ones will help to consolidate these results.

Answer: We agree with the reviewer that this experiment would support the relevance of our findings from a clinical point of view but unfortunately, we do not have access to the samples from the COG study since the data was downloaded from public repositories.

3-Is a bit confusing to follow all the different clustering and its use for the DFS studies. In the COG study 9404 (with survival data) it was used P1, P2, etc and the TARGET with 3 clusters based on Q1-Q4. Can the authors show either or both in COG study 9404 (with survival data) using Q1-Q4 to plot DSF and/or the TARGET with similar clustering as P1 to P5 in the COG study 9404 study? Maybe when using Q1-Q4 clustering is not predictive even in the COG study 9404 (with survival data) cohort? If so, it takes away the power of this G1/G2 ssGSEA score analysis.
This reviewer questions the importance of this analysis as even patients located in Q4 (high in G1 score) can be in remission and those that responded but then relapsed (seen more clearly in the TARGET cohort) which means using this signature does not necessary can predict that a patient can be refractory of the treatment used.
Answer: As indicated by the reviewer, the upregulation of G1 geneset does not necessarily predict treatment failure (refraction), but identifies patients with a higher probability of refraction, which will potentially benefit from the administration of β-catenin inhibitors in combination with chemotherapy, and could be used when selecting patients for future clinical trials.
Following the reviewer suggestion, we have now established survival curves comparing patients belonging to Q4 cluster (high G1) vs the rest, for both our discovery cohort and TARGET. We find reduced DFS and OS in the Q4 group compared to the other patients of the discovery cohort (as it contains most of the refractory P1 group), while there is no impact in survival curves using the same criteria in the TARGET group including a single refractory patient. We have added the OS and DFS using this clustering in the manuscript (Figure S4d and S4e).

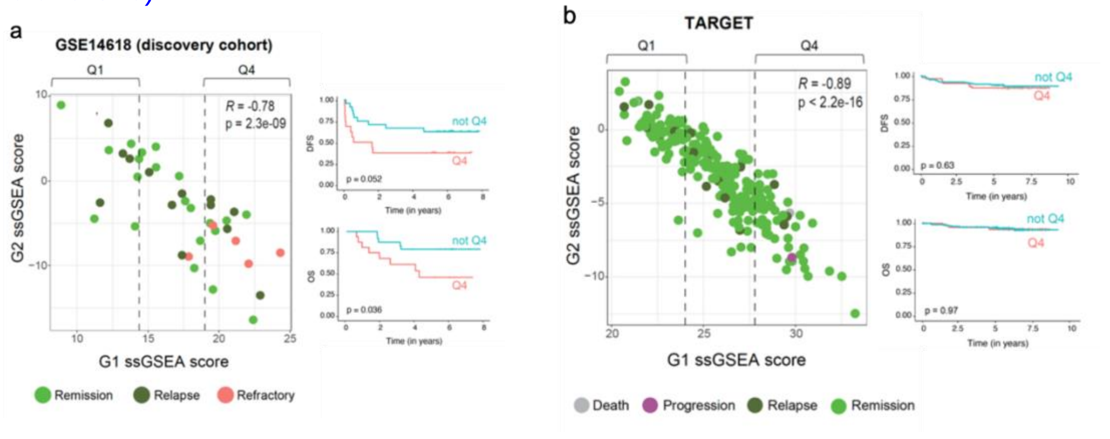

Fig. *2 Correlation of ssGSEA enrichment for G1 and G2 modules from Fig 3a in patients from the discovery (a, left) and TARGET (b, left) cohorts, corresponding to the panels shown in Fig 4. Kaplan-Meier curves (right panels in a and b) represent disease free probability (DFS) and overall survival (OS) for groups of patients in Q4 compared to the other patients.*

We have also clustered TARGET patients using a non-supervised hierarchical model based on the expression of the 79 β-catenin direct transcriptional targets, same as in Figure 3 for the discovery cohort. This classification is not informative since there is only one refractory patient and patient groups do not show different outcomes. This data is not shown in the

manuscript.

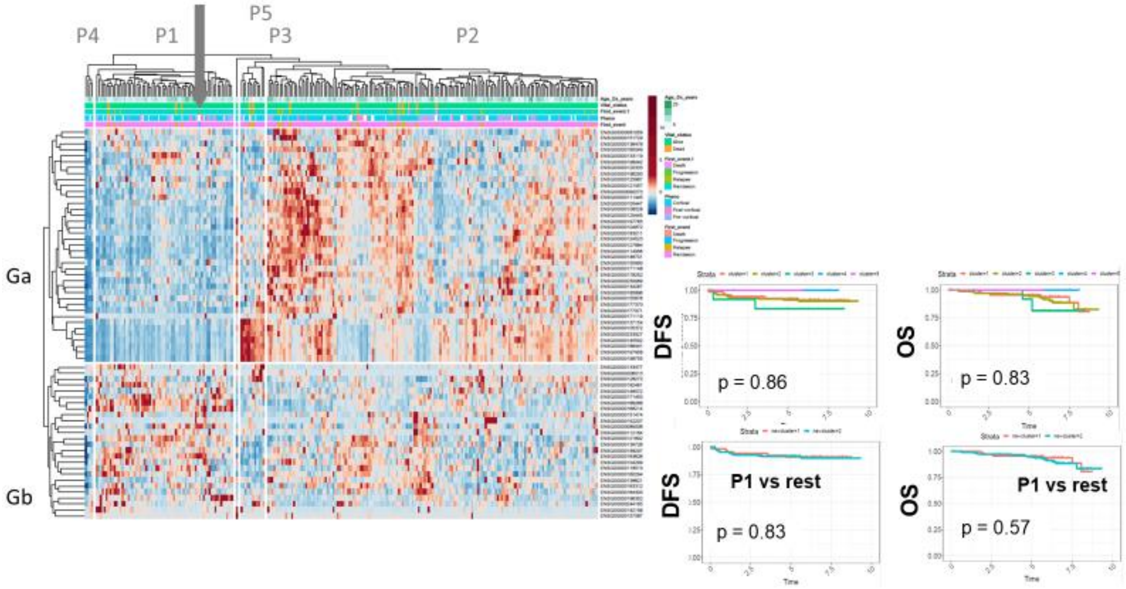

***Fig**. 3 Non-supervised hierarchical classification of T-ALL patients from TARGET cohort according to the expression of the 79 β-catenin targets downregulated after β-catenin knockdown and Kaplan-Meier curves representing disease free (DSF) and overall free (OS) probability for groups of patients generated. Grey arrow marks the position of the single refractory patient in the TARGET cohort*

Also is unclear how the P1 to P5 clustering was originated, can the authors clarify?

Answer: We have now added a clearer explanation of the clustering strategies both in the methods section and in the manuscript body. For clarity, references to the different clusters have been refined throughout the manuscript and figures.

In the methods section, we have added:

'Patients were clustered using a non-supervised hierarchical model based on the expression of different groups of β-catenin targets. All non-supervised hierarchical clusterings included were obtained using the pheatmap R package with default parameters (euclidean distance and complete clustering method). In each case, the number of patient and gene clusters were decided based on visual inspection of different expression patterns among clusters. In 3A, we produced a minimal predictive β-catenin signature and we adjusted the number of patient clusters to include the majority of refractory cases within the same cluster. Survival curves were performed with the survminer R package. Hazard ratios were estimated with the peperr R package.'

In the text:

'We applied a new non-supervised hierarchical clustering (Fig 3a) and the patients' dendrogram was 'arbitrarily' divided into five clusters (P1-P5) with P1 including 5 out of the 6 refractory patients (Fig 3a and 3b) showing a poor DFS (p=0.0039) and overall survival (OS) (p=0.025) cluster (Fig 3c).'

4-In the TARGET cohort, there was/is no difference in DFS between the different clusters (Fig. 4d and e) potentially due to the lack of refractory samples in cluster A that are able to augment this difference seen in the COG study 9404 (P1 cluster vs the rest). Or maybe the use of this clustering based on G1/G2 ssGSEA score is not powerful enough? If using a different clustering (as requested above) even with similar results are found perhaps is

better to move as supplementary as there is insufficient refractory samples in this cohort hence the data is not that strong.
Answer: As commented above, clustering based on G1/G2 ssGSEA score is only powerful to identify refractory patients, but non-supervised clustering is not informative. Following reviewer suggestion, we have moved Kaplan-Meier analysis of TARGET patients based on this clustering to supplementary material (Fig S4d and S4e) .

Minor:
-supplementary figures need attention, as after pdf conversion many graphs were distorted/misplaced hence it was difficult to analyse the data.
Answer: We apologize for that inconvenience and have corrected them.

Referee #3 (Remarks for Author):

This is an interesting study characterizing beta-catenin functions in resistance to therapy with a focus on RNA binding-related genes as critical targets of the catenin signaling pathway. As resistance to therapy is an unmet need in T-ALL, the focus of the study is of interest for scientists and physicians alike.
Answer: We really appreciate the positive comments from the reviewer.

Nevertheless, a few parts of the current version of the study need to be better connected, especially with regards to the role of the identified catenin targets. Specific comments and questions are shown below:

Is the phenomenon described a general T-ALL phenomenon or it applies to a specific subtype?
Answer:
The minimal β-catenin signature is specifically informative of therapy failure among those patients within the poor-prognosis immature phenotypes ETP and ABD, but it represents a more powerful prognosis factor than ETP+ABC classification alone ($HR_{cluster\ P1}$=18.18; p =0.003 vs. $HR_{ETP+ABD}$=7.64; p=0.03 (Fig 3e)). Consistent with this, despite only 3 out of 10 ETP patients and 3 out of 5 ABC patients in the discovery cohort were considered as refractory, all the refractory were located in the P1 cluster (Fig 3a) (see graph and table below).
We thank the reviewer for addressing this issue which is now mentnioned in the discussion section.

'We also found a significant association between the phenotypes ETP and ABD with the outcome in the discovery cohort (HR=7.64; p=0.03), being all the refractory cases inside these T-ALL subtypes (Fig 3e). ETP and ABD groups are both immature T-ALLs and patients falling into these phenotypes frequently display a dismal outcome and higher rates of chemotherapy refraction (Coustan-Smith Lancet Oncol. 2009; Gutierrez J Clin Oncol. 2010). In this study we observed that the minimal β-catenin signature is specifically informative of therapy failure even within the group of patients with an immature phenotype (Fig 3a) but it represents a more powerful prognosis factor than ETP plus ABD alone (HR cluster P1=18.18; p =0.003 vs. HR ETP+ABD=7.64; p=0.03) (Fig 3e). Whether the β-catenin activity contributes to the worse outcome of immature T-ALLs requires further research.'

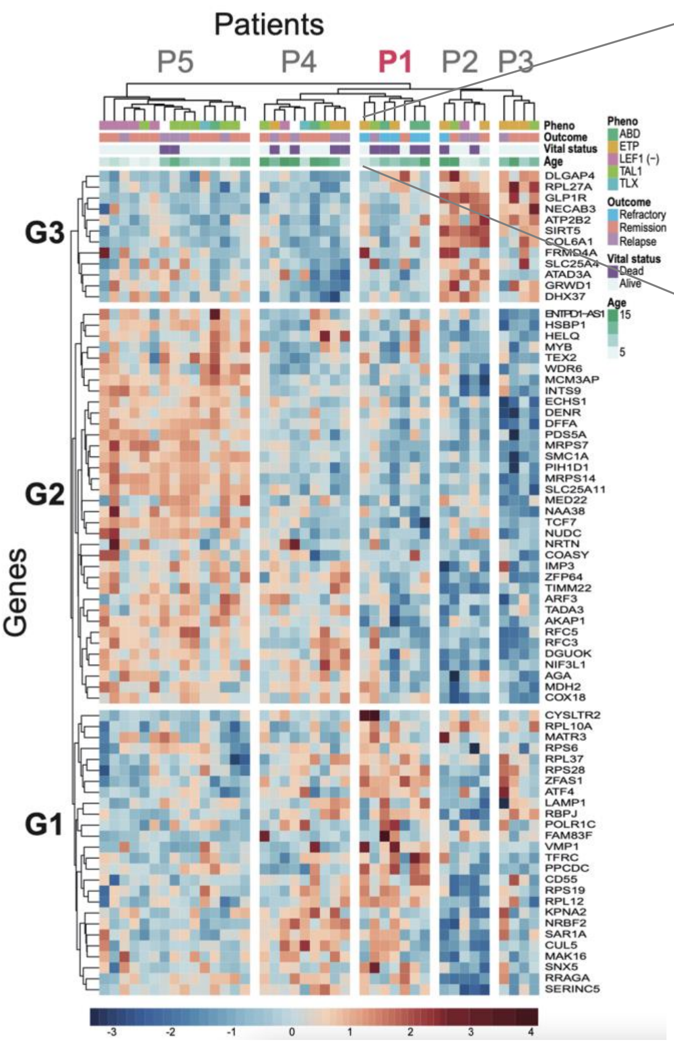

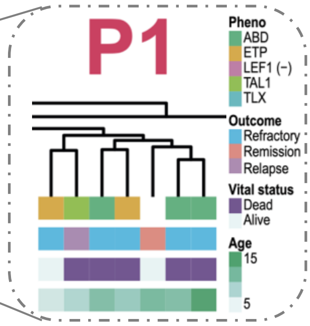

	Refractory (non-responders)	Non-refractory (responders)
Cluster P1	5 (71.43%)	2 (28.57%)
ETP	3 (30%)	7 (70%)
ABD	3 (60%)	2 (40%)
ETP+ABD	6 (40%)	9 (60%)

The phenotype of the refractory patients in the other cohorts analyzed are either not immature (cortical T-ALL in the case of EGAS00001000536) or not reported. Further studies with additional and fully annotated datasets are needed to confirm the general application of our results.

The authors showed that catenin affects the levels of RNA binding proteins, such as RBM39. Please discuss the roles of those proteins and their impact on the transcriptome.

Answer: We agree with the reviewer that this is an important question that deserves further discussion, and thus has been now included in the manuscript.

'Enriched RNA processing functions include different types of RNA-binding proteins (RBP). Aberrant RBPs functioning may result in global remodeling of the transcriptome and the proteome and it has been implicated in human disease and tumorigenesis (reviewed in Mohibi et al, Pharmacol Ther, 2019 and Hodson et al, Blood 2019). In AML, a comprehensive CRISPR screening uncovered the upregulation of a network of RBPs important for AML survival and identified RBM39 as a critical and targetable element (Wang et al, Cancer Cell 2019). Here, we also detected downregulation of RBM39 in β-catenin knockdown cells, although it was not identified as a direct β-catenin chromatin target.'

To further investigate the possible impact of RNA processing downstream of *β-catenin, we have generated* an interaction map including all identified elements in this functional group (see figure below, Fig 5). This analysis suggests that β-catenin has a pleiotropic role in the coordination and amplification of several processes likely contributed by RBPs such as splicing, mitochondrial translation, rRNA processing and others.

Can silencing of any of those proteins recapitulate the molecular effect in the transcriptome caused by b-catenin silencing?

To showcase the importance of the downstream targets of catenin pathway, the authors need to rescue the phenotype of b-catenin silencing via expressing at least one of the downstream targets.

Answer: This is an important point; however, we find an extensive representation of downstream β-catenin targets annotated to RNA processing functions, including 273 RBPs (Gerstberger Nat Rev Genet. 2014) with no clear indication of individual elements that could essentially contribute to this function (see below, Figure 4).

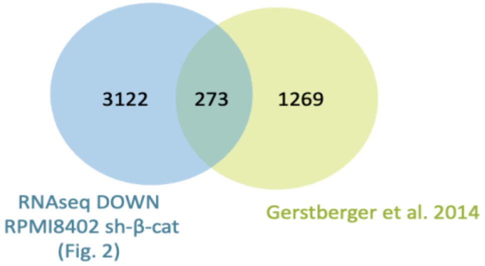

Fig.4 Venn Diagram showing the number of differentially downregulated genes in RPMI8402 cells upon β-catenin knockdown (blue) included in the RNA-binding proteins census in Gerstberger et al, 2014.

Next, we tried to narrow down the list of RBPs that may recapitulate the effects of β-catenin knockdown by building a protein-protein interaction (PPI) network (including all significantly down-regulated genes in our RNAseq which were annotated to RNA processing-related GO terms; n = 735 genes from the initial 3,395), the PPI analysis revealed an imbricated web of elements with no clear core hits (see figure below). These results supports the idea that the global impact on RNA and protein synthesis modulated by β-catenin is orchestrated by a network of RBPs and other RNA-processing elements, rather than by individual master regulators (see Figure 5 below, not included in the manuscript). From these data, we strongly believe that identification of targetable RBPs across our signature should require a systematic inspection (as done in Wang et al. Cancer Cell 2019) that should be addressed in a follow-up project.

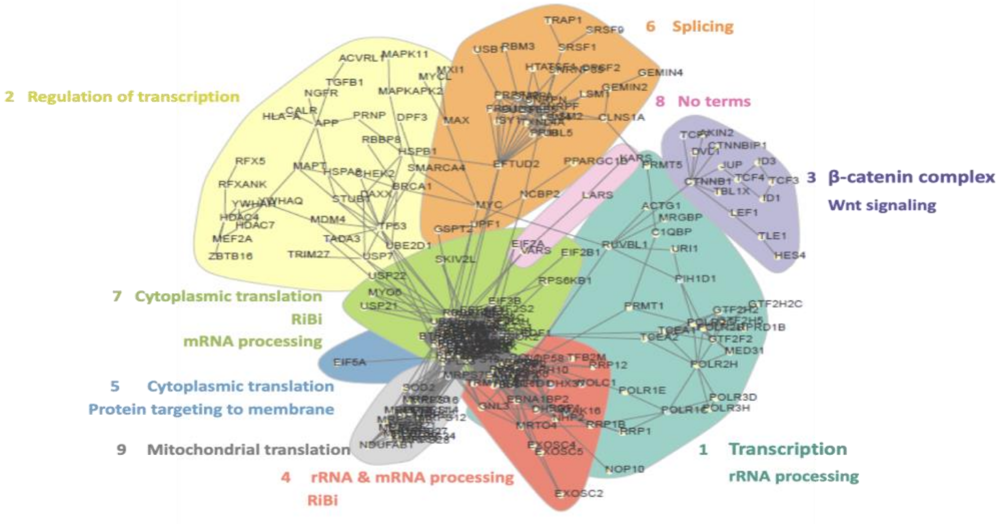

Fig. 5 Largest Connected Component (LCC, n = 243 genes) obtained from the Protein-Protein Interaction (PPI) network. A PPI was built with STRINGdb v11.5 (https://string-db.org) with the list of 735 β-catenin downstream targets (down-regulated differentially expressed genes associated to RNA processing GO terms in sh-β-catenin RPMI8402 cells). LCC is composed of 243 genes and integrated by 9 communities based on the Louvain-Leiden clustering method (marked in different colors). The 9 communities are annotated to different RNA processing terms as depicted by an overrepresentation functional analysis (enrichR R package v3.1). Network analysis conducted with igraph R package v1.3.4.

Still, identification of targetable RBPs across our signature should require a systematic inspection (as done in Wang et al. Cancer Cell 2019) that should be addressed in a follow-up project.

Ideally the authors need to show protein levels of the RBPs, as it is well known that factors implicated in RNA metabolism can be regulated at the post-transcriptional level (via cross-talk of their activities, nonsense mediated decay etc).
Answer: Here, we described an RNA biogenesis program modulated by β-catenin through transcriptional effects. Thus, we have focused our efforts in transcriptomic analysis. However, we cannot rule out the possibility that other mechanisms as post-translational modifications also contribute to the final levels of the RBPs transcriptionally modulated by β-catenin.

Overall, T-ALL transcription factors (TFs) co-bind genomic targets (see Sanda et al., Cancer Cell, 2012 as an example). Are the loci in question co-bound by other leukemia TFs, such as NOTCH1, or TAL1? This could mean that targeting those proteins might affect the targets in question. The authors can use published ChIP-Seq data to answer this. Can modulation of those TFs lead to the same effect as catenin silencing? The authors can also analyze publicly available data to answer this.
Answer: To address this question, we have performed motif enrichment analysis of β-catenin DNA binding regions. We uncovered ZBTB33/Kaiso as the most represented motif in these regularory regions (Fig 1c, Table S1) as well as a significant enrichment of the IRF6 and SPI1 motifs, the latest also annotated as secondary SMAD3 motif (Table S1). We propose these factors as the most likely candidates to act as chromatin co-factors for β-catenin. We have included some experimental evidences indicating the involvement of Kaiso in β-catenin-dependent transcription (Figure 2g). We also found that factors such as BRCA1, YY1 or MYC could bind the same regulatory regions (see figure 6 below), which will be further investigated.

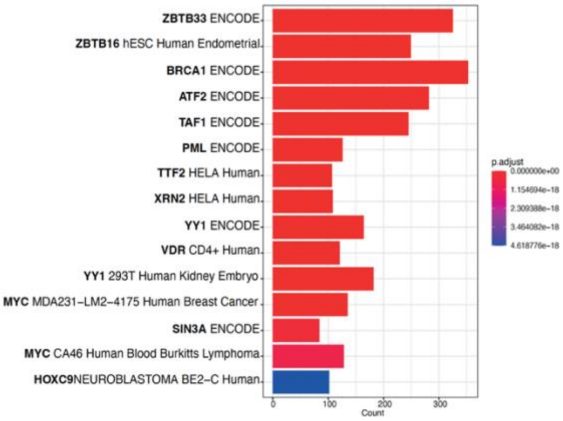

Fig.6 Barplot with the top 15 significantly overrepresented TFs. Results are sorted by adjusted p-value (FDR). TFEA included 'ChEA 2022' and 'ENCODE and ChEA consensus TFs from ChIP-X' databases which were directly downloaded from https://maayanlab.cloud/Enrichr/#libraries (Xie et al., Current Protocols 2021).

In addition, and following the reviewer's suggestion, we have compared the β-catenin direct target genomic regions with the ones from TAL1, RUNX1, GATA3 and LMO1 from Sanda et al. and public dataset of NOTCH1 ChIPseq performed in CUTLL1 cells (obtained from https://chip-atlas.org). The genomics sites bound by β-catenin showed significant overlapping with those from RUNX1 and NOTCH1 (adj p-val < 0.05, see table below). Overlap between β-catenin and LMO1 was also significant but limited, with only 7 convergent regions. The overlap between these factors and β-catenin targets may indicate a co-regulatory chromatin activity, although functional experiments should be performed in our system to test this point.

Table 1: Pairwise comparison of β-catenin-bound regions and TFs-bound regions in T-All cell lines from Sanda et al., Cancer Cell 2012 and public data from ChIP ATLAS (only for NOTCH1). Significant overlapping is detected when comparing to LMO1, RUNX1 and NOTCH1 data (FDR adj p-value < 0.05). A minimum of 1-base was considered for detecting overlap between two regions. The background null distribution of the overlap was estimated per TF by means of the generation of random data with the same number of regions and corresponding widths as the original TF data. Randomized regions were restricted to any annotated region included in the Open Regulatory Annotation Database (Lesurf et al., NAR 2016). P-value was calculated by the probability of observing a higher than observed overlap.

TF		Regions in TF	β-catenin overlapping regions	β-catenin overlapping regions in proximal promoter (<=1kb)	Adjusted P-value
Jurkat (Sanda et al. 2012)	TAL1	6273	6 (1,4%)	5 (83%)	1
	HEB	4803	6 (1,4%)	4 (67%)	1
	E2A	2783	4 (0,9%)	3 (75%)	0.7193
	LMO1	3414	8 (1,8%)	7 (88%)	**0.0240**
	GATA3	8645	12 (2,85)	10 (83%)	0.3437
	RUNX1	9176	60 (13,9%)	57 (95%)	**0.0080**
CUTLL1 (ChIP-Atlas)	NOTCH1	3229	40 (9,2%)	35 (88%)	**0.0080**

In this line, we previously found that β-catenin and Notch are required for MYC activation in T-ALL cells (Gekas et al, 2016). Myc-associated gene sets are significantly overrepresented in the TFEA and in the PPI network, Myc is one of the factors connecting splicing/rRNA processing and regulation of transcription apart from being a well-known regulator of RNA metabolism and global cellular growth (Sabò et al, Nature 2014; Stine et al, Cancer Discovery 2015).

We have now revised this part in the discussion:

'We have also examined whether other factors important for T-ALL are able to collaborate with β-catenin in the chromatin. Computational comparison of the β-catenin target regions with regions bound by T-ALL factors (TAL1, GATA3, LMO1, Runx1 from Sanda et al, Cancer Cell 2012; Notch1 from https://chip-atlas.org) showed a significant coincidence between β-cateni-n and Runx1- or Notch1-bound regions (13.9% and 9.2% of overlapping peaks, respectively) (data not shown). Contribution of these factors to β-catenin-dependent transcription will be further investigated'.

5th Dec 2022

Dear Dr. Bigas,

Thank you for the submission of your manuscript to EMBO Molecular Medicine, and please accept my apologies for the delay in getting back to you as one referee needed more time to complete his/her review. As you will see below, the referees are now supportive of publication, and I am therefore pleased to inform you that we will be able to accept your manuscript once the following editorial points will be addressed:

1/ Please address referee #3 remaining minor comment.

2/ Manuscript text:
- Please remove the highlighted text, accept all previous changes, and only keep in track changes mode any new modification.
- Human data: include a statement that informed consent was obtained from all subjects and that the experiments conformed to the principles set out in the WMA Declaration of Helsinki and the Department of Health and Human Services Belmont Report.
- Data Availability: Thank you for depositing your datasets in a public repository. Please note that they must be public before acceptance of the manuscript. Please also note that this section is restricted to new primary data that are part of this study and not datasets previously generated and already published.

3/ Figures.
- Please indicate for all figures or in their legends the exact p= values, not a range. Some people found that to keep the figures clear, providing a supplemental table in an Appendix with all exact p-values was preferable. You are welcome to do this if you want to.
- Appendix: please provide a table of content.

4/ At EMBO Press we encourage authors to provide source data for the main figures. Numerical data should be provided as individual .xls or .csv files (including a tab describing the data). For blots or microscopy, uncropped images should be submitted (using a zip archive if multiple images need to be supplied for one panel). Additional information on source data and instruction on how to label the files are available at
.

5/ Checklist:
- Please carefully check the section about Dual Use Research of Concern and either indicate whether the information is included in the manuscript or remove the "Material and Methods" on the right boxes.
- Please also make sure that the Data Availability section matches the information provided in the manuscript.

6/ Please provide a Paper Explained section, that emphasizes the major findings in the paper and their medical implications for the non-specialist reader. Please provide a summary of your article highlighting
- the medical issue you are addressing,
- the results obtained and
- their clinical impact.
This may be edited to ensure that readers understand the significance and context of the research. Please refer to any of our published articles for an example.

7/ Every published paper now includes a 'Synopsis' to further enhance discoverability. Synopses are displayed on the journal webpage and are freely accessible to all readers. They include a short stand first (maximum of 300 characters, including space) as well as 2-5 one-sentences bullet points that summarizes the paper. Please write the bullet points to summarize the key NEW findings. They should be designed to be complementary to the abstract - i.e. not repeat the same text. We encourage inclusion of key acronyms and quantitative information (maximum of 30 words / bullet point). Please use the passive voice. Please attach these in a separate file or send them by email, we will incorporate them accordingly.

Please also suggest a striking image or visual abstract to illustrate your article as a PNG file 550 px wide x 300-600 px high.

8/ As part of the EMBO Publications transparent editorial process initiative (see our Editorial at http://embomolmed.embopress.org/content/2/9/329), EMBO Molecular Medicine will publish online a Review Process File (RPF) to accompany accepted manuscripts.
This file will be published in conjunction with your paper and will include the anonymous referee reports, your point-by-point response and all pertinent correspondence relating to the manuscript. Let us know whether you agree with the publication of the RPF and as here, if you want to remove or not any figures from it prior to publication.
Please note that the Authors checklist will be published at the end of the RPF.

I look forward to receiving your revised manuscript.

With kind regards,

Lise Roth

Lise Roth, PhD
Senior Editor
EMBO Molecular Medicine

***** Reviewer's comments *****

Referee #1 (Remarks for Author):

Author have largely addressed my concern.

Referee #2 (Remarks for Author):

Although the authors were not able to perform the exp. (point/question 2) that this reviewer requested due to the lack of samples, overall the authors answered the remaining of my "concerns" and questions appropriately. The presented results have enough weight and merit to support their hypothesis.

Referee #3 (Comments on Novelty/Model System for Author):

The authors are presenting an improved manuscript from a technical and scientific standpoint.

Referee #3 (Remarks for Author):

The authors have adequately addressed my comments. Regarding the comment "Ideally the authors need to show protein levels of the RBPs...", I would add their answer as part of the discussion in the manuscript, as the actual protein levels of these factors are critical for the disease pathophysiology and a follow up story would be very informative. It is possible, for instance, that these factors have post-transcriptional regulatory mechanisms that compensate for their transcriptional regulation by the b-catenin pathway.

Reviewer #3

The authors have adequately addressed my comments. Regarding the comment "Ideally the authors need to show protein levels of the RBPs...", I would add their answer as part of the discussion in the manuscript, as the actual protein levels of these factors are critical for the disease pathophysiology and a follow up story would be very informative. It is possible, for instance, that these factors have post-transcriptional regulatory mechanisms that compensate for their transcriptional regulation by the b-catenin pathway.

We thank the reviewers for the comments.
We have addressed this comment by including in the discussion, Page 11, lane 302:

‘*However, since post-translational modifications may affect the protein levels of RBPs, further analysis of these proteins in the absence of β-catenin should clarify its involvement in chemotherapy resistance.*’

13th Dec 2022

Dear Dr. Bigas,

Thank you for sending your revised synopsis. I am pleased to inform you that your manuscript is accepted for publication and is now being sent to our publisher to be included in the next available issue of EMBO Molecular Medicine.

Please read below for additional IMPORTANT information regarding your article, its publication and the production process.

Congratulations on your interesting work!

With kind regards,

Lise Roth

Lise Roth, Ph.D
Senior Editor
EMBO Molecular Medicine

Follow us on Twitter @EmboMolMed
Sign up for eTOCs at embopress.org/alertsfeeds

*** *** *** IMPORTANT INFORMATION *** *** ***

SPEED OF PUBLICATION
The journal aims for rapid publication of papers, using using the advance online publication "Early View" to expedite the process: A properly copy-edited and formatted version will be published as "Early View" after the proofs have been corrected. Please help the Editors and publisher avoid delays by providing e-mail address(es), telephone and fax numbers at which author(s) can be contacted.

Should you be planning a Press Release on your article, please get in contact with embomolmed@wiley.com as early as possible, in order to coordinate publication and release dates.

LICENSE AND PAYMENT:

All articles published in EMBO Molecular Medicine are fully open access: immediately and freely available to read, download and share.

EMBO Molecular Medicine charges an article processing charge (APC) to cover the publication costs. You, as the corresponding author for this manuscript, should have already received a quote with the article processing fee separately. Please let us know in case this quote has not been received.

Once your article is at Wiley for editorial production you will receive an email from Wiley's Author Services system, which will ask you to log in and will present you with the publication license form for completion. Within the same system the publication fee can be paid by credit card, an invoice, pro forma invoice or purchase order can be requested.

Payment of the publication charge and the signed Open Access Agreement form must be received before the article can be published online.

PROOFS

You will receive the proofs by e-mail approximately 2 weeks after all relevant files have been sent o our Production Office. Please return them within 48 hours and if there should be any problems, please contact the production office at embopressproduction@wiley.com.

Please inform us if there is likely to be any difficulty in reaching you at the above address at that time. Failure to meet our deadlines may result in a delay of publication.

All further communications concerning your paper proofs should quote reference number EMM-2022-16554-V3 and be directed to the production office at embopressproduction@wiley.com.

Thank you,

Lise Roth, Ph.D
Senior Editor
EMBO Molecular Medicine

EMBO Press Author Checklist

Corresponding Author Name: Anna Bigas	
Journal Submitted to: EMBO Molecular Medicine	
Manuscript Number: EMM-2022-16554-V2	

USEFUL LINKS FOR COMPLETING THIS FORM
The EMBO Journal - Author Guidelines
EMBO Reports - Author Guidelines
Molecular Systems Biology - Author Guidelines
EMBO Molecular Medicine - Author Guidelines

Reporting Checklist for Life Science Articles (updated January 2022)
This checklist is adapted from Materials Design Analysis Reporting (MDAR) Checklist for Authors. MDAR establishes a minimum set of requirements in transparent reporting in the life sciences (see Statement of Task: 10.31222/osf.io/9sm4x). Please follow the journal's guidelines in preparing your manuscript.
Please note that a copy of this checklist will be published alongside your article.

Abridged guidelines for figures
1. Data
The data shown in figures should satisfy the following conditions:
- the data were obtained and processed according to the field's best practice and are presented to reflect the results of the experiments in an accurate and unbiased manner.
- ideally, figure panels should include only measurements that are directly comparable to each other and obtained with the same assay.
- plots include clearly labeled error bars for independent experiments and sample sizes. Unless justified, error bars should not be shown for technical replicates.
- if n<5, the individual data points from each experiment should be plotted. Any statistical test employed should be justified.
- Source Data should be included to report the data underlying figures according to the guidelines set out in the authorship guidelines on Data Presentation.

2. Captions
Each figure caption should contain the following information, for each panel where they are relevant:
- a specification of the experimental system investigated (eg cell line, species name).
- the assay(s) and method(s) used to carry out the reported observations and measurements.
- an explicit mention of the biological and chemical entity(ies) that are being measured.
- an explicit mention of the biological and chemical entity(ies) that are altered/varied/perturbed in a controlled manner.
- the exact sample size (n) for each experimental group/condition, given as a number, not a range;
- a description of the sample collection allowing the reader to understand whether the samples represent technical or biological replicates (including how many animals, litters, cultures, etc.).
- a statement of how many times the experiment shown was independently replicated in the laboratory.
- definitions of statistical methods and measures:
  - common tests, such as t-test (please specify whether paired vs. unpaired), simple χ2 tests, Wilcoxon and Mann-Whitney tests, can be unambiguously identified by name only, but more complex techniques should be described in the methods section;
  - are tests one-sided or two-sided?
  - are there adjustments for multiple comparisons?
  - exact statistical test results, e.g., P values = x but not P values < x;
  - definition of 'center values' as median or average;
  - definition of error bars as s.d. or s.e.m.

Please complete ALL of the questions below.
Select "Not Applicable" only when the requested information is not relevant for your study.

Materials

Newly Created Materials	Information included in the manuscript?	In which section is the information available? (Reagents and Tools Table, Materials and Methods, Figures, Data Availability Section)
New materials and reagents need to be available; do any restrictions apply?	Yes	Material and Methods and Data Availability

Antibodies	Information included in the manuscript?	In which section is the information available? (Reagents and Tools Table, Materials and Methods, Figures, Data Availability Section)
For **antibodies** provide the following information:<br>- Commercial antibodies: RRID (if possible) or supplier name, catalogue number and or/clone number<br>- Non-commercial: RRID or citation	Yes	Material and Methods and Suplementary information

DNA and RNA sequences	Information included in the manuscript?	In which section is the information available? (Reagents and Tools Table, Materials and Methods, Figures, Data Availability Section)
**Short novel DNA or RNA including primers, probes**: provide the sequences.	Yes	Material and Methods and Suplementary information

Cell materials	Information included in the manuscript?	In which section is the information available? (Reagents and Tools Table, Materials and Methods, Figures, Data Availability Section)
**Cell lines:** Provide species information, strain. Provide accession number in repository **OR** supplier name, catalog number, clone number, and/**OR** RRID.	Yes	Material and Methods
**Primary cultures:** Provide species, strain, sex of origin, genetic modification status.	Not Applicable	
Report if the cell lines were recently **authenticated** (e.g., by STR profiling) and tested for mycoplasma contamination.	Yes	Material and Methods

Experimental animals	Information included in the manuscript?	In which section is the information available? (Reagents and Tools Table, Materials and Methods, Figures, Data Availability Section)
**Laboratory animals or Model organisms:** Provide species, strain, sex, age, genetic modification status. Provide accession number in repository **OR** supplier name, catalog number, clone number, **OR** RRID.	Yes	Material and Methods
**Animal observed in or captured from the field:** Provide species, sex, and age where possible.	Not Applicable	
Please detail **housing and husbandry conditions**.	Yes	Material and Methods

Plants and microbes	Information included in the manuscript?	In which section is the information available? (Reagents and Tools Table, Materials and Methods, Figures, Data Availability Section)
**Plants:** provide species and strain, ecotype and cultivar where relevant, unique accession number if available, and source (including location for collected wild specimens).	Not Applicable	
**Microbes:** provide species and strain, unique accession number if available, and source.	Not Applicable	

Human research participants	Information included in the manuscript?	In which section is the information available? (Reagents and Tools Table, Materials and Methods, Figures, Data Availability Section)
If collected and within the bounds of privacy constraints report on age, sex and gender or ethnicity for all study participants.	Not Applicable	

Core facilities	Information included in the manuscript?	In which section is the information available? (Reagents and Tools Table, Materials and Methods, Figures, Data Availability Section)
If your work benefited from core facilities, was their service mentioned in the acknowledgments section?	Yes	acknowledgments

Design

Study protocol	Information included in the manuscript?	In which section is the information available? (Reagents and Tools Table, Materials and Methods, Figures, Data Availability Section)
If study protocol has been **pre-registered, provide DOI in the manuscript**. For clinical trials, provide the trial registration number **OR** cite DOI.	Yes	
Report the **clinical trial registration number** (at ClinicalTrials.gov or equivalent), where applicable.	Not Applicable	

Laboratory protocol	Information included in the manuscript?	In which section is the information available? (Reagents and Tools Table, Materials and Methods, Figures, Data Availability Section)
Provide DOI OR other citation details if **external detailed step-by-step protocols** are available.	Not Applicable	

Experimental study design and statistics	Information included in the manuscript?	In which section is the information available? (Reagents and Tools Table, Materials and Methods, Figures, Data Availability Section)
Include a statement about **sample size** estimate even if no statistical methods were used.	Yes	Material and Methods
Were any steps taken to minimize the effects of subjective bias when allocating animals/samples to treatment (e.g. **randomization procedure**)? If yes, have they been described?	Yes	Material and Methods
Include a statement about **blinding** even if no blinding was done.	Yes	Material and Methods
Describe **inclusion/exclusion criteria** if samples or animals were excluded from the analysis. Were the criteria pre-established?<br><br>If sample or data points were omitted from analysis, report if this was due to attrition or intentional exclusion and provide justification.	Yes	Material and Methods
For every figure, are **statistical tests** justified as appropriate? Do the data meet the assumptions of the tests (e.g., normal distribution)? Describe any methods used to assess it. Is there an estimate of variation within each group of data? Is the variance similar between the groups that are being statistically compared?	Yes	Material and Methods and Figures

Sample definition and in-laboratory replication	Information included in the manuscript?	In which section is the information available? (Reagents and Tools Table, Materials and Methods, Figures, Data Availability Section)
In the figure legends: state number of times the experiment was **replicated** in laboratory.	Yes	Figures and Material and Methods
In the figure legends: define whether data describe **technical or biological replicates**.	Yes	figures

Ethics

Ethics	Information included in the manuscript?	In which section is the information available? (Reagents and Tools Table, Materials and Methods, Figures, Data Availability Section)
Studies involving **human participants**: State details of **authority granting ethics approval** (IRB or equivalent committee(s), provide reference number for approval.	Not Applicable	
Studies involving **human participants**: Include a statement confirming that **informed consent** was obtained from all subjects and that the experiments conformed to the principles set out in the WMA Declaration of Helsinki and the Department of Health and Human Services Belmont Report.	Not Applicable	
Studies involving **human participants:** For publication of **patient photos**, include a statement confirming that consent to publish was obtained.	Not Applicable	
Studies involving experimental **animals**: State details of **authority granting ethics approval** (IRB or equivalent committee(s), provide reference number for approval. Include a statement of compliance with ethical regulations.	Yes	Material and Methods
Studies involving **specimen and field samples:** State if relevant **permits** obtained, provide details of authority approving study; if none were required, explain why.	Not Applicable	

Dual Use Research of Concern (DURC)	Information included in the manuscript?	In which section is the information available? (Reagents and Tools Table, Materials and Methods, Figures, Data Availability Section)
Could your study fall under dual use research restrictions? Please check biosecurity documents and list of **select agents and toxins** (CDC): https://www.selectagents.gov/sat/list.htm	Not Applicable	
If you used a select agent, is the security level of the lab appropriate and reported in the manuscript?	Not Applicable	
If a study is subject to dual use research of concern regulations, is the name of the **authority granting approval and reference number** for the regulatory approval provided in the manuscript?	Not Applicable	

Reporting

The MDAR framework recommends adoption of discipline-specific guidelines, established and endorsed through community initiatives. Journals have their own policy about requiring specific guidelines and recommendations to complement MDAR.

Adherence to community standards	Information included in the manuscript?	In which section is the information available? (Reagents and Tools Table, Materials and Methods, Figures, Data Availability Section)
State if relevant guidelines or checklists (e.g., **ICMJE, MIBBI, ARRIVE, PRISMA**) have been followed or provided.	Not Applicable	
For **tumor marker prognostic studies**, we recommend that you follow the **REMARK** reporting guidelines (see link list at top right). See author guidelines, under 'Reporting Guidelines'. Please confirm you have followed these guidelines.	Not Applicable	
For **phase II and III randomized controlled trials**, please refer to the **CONSORT** flow diagram (see link list at top right) and submit the CONSORT checklist (see link list at top right) with your submission. See author guidelines, under 'Reporting Guidelines'. Please confirm you have submitted this list.	Not Applicable	

Data Availability

Data availability	Information included in the manuscript?	In which section is the information available? (Reagents and Tools Table, Materials and Methods, Figures, Data Availability Section)
Have **primary datasets** been deposited according to the journal's guidelines (see 'Data Deposition' section) and the respective accession numbers provided in the Data Availability Section?	Yes	Data Availability and Material and Methods
Were **human clinical and genomic datasets** deposited in a public access-controlled repository in accordance to ethical obligations to the patients and to the applicable consent agreement?	Not Applicable	
Are **computational models** that are central and integral to a study available without restrictions in a machine-readable form? Were the relevant accession numbers or links provided?	Yes	Data Availability and Material and Methods
If publicly available data were reused, provide the respective **data citations in the reference list**.	Yes	Data Availability and Material and Methods and Reference list
