# Supplementary material for: β‐Catenin activity induces an RNA biosynthesis program promoting therapy resistance in T‐cell acute lymphoblastic leukemia
Source: EMBO Mol Med. 2023 Jan 4;15(2):e16554. doi: 10.15252/emmm.202216554 (PMC9906382; doi:10.15252/emmm.202216554)

Uncropped scans of all blots in Expanded View

Figure EV1a

Figure EV1a: anti-β-catenin

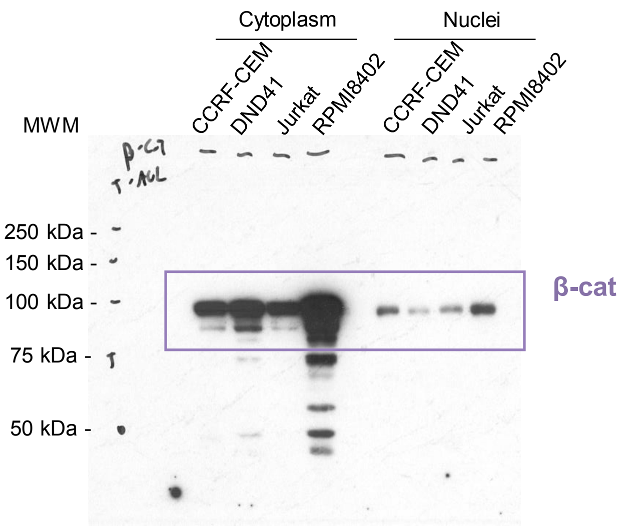

Figure EV1a: anti-active β-catenin

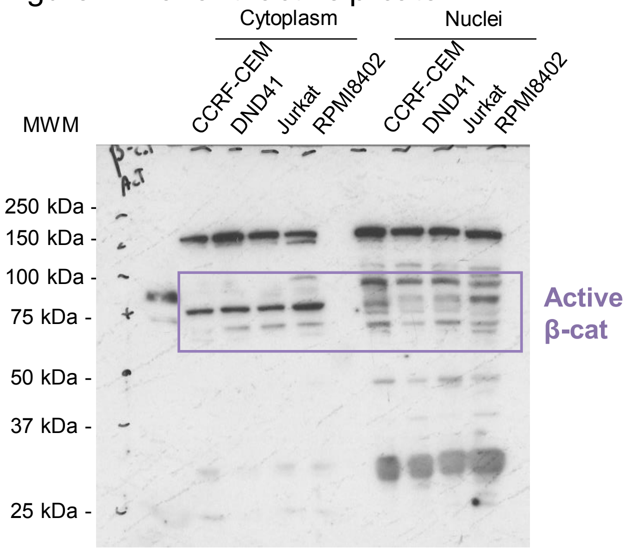

Figure EV1a: anti-TCF1

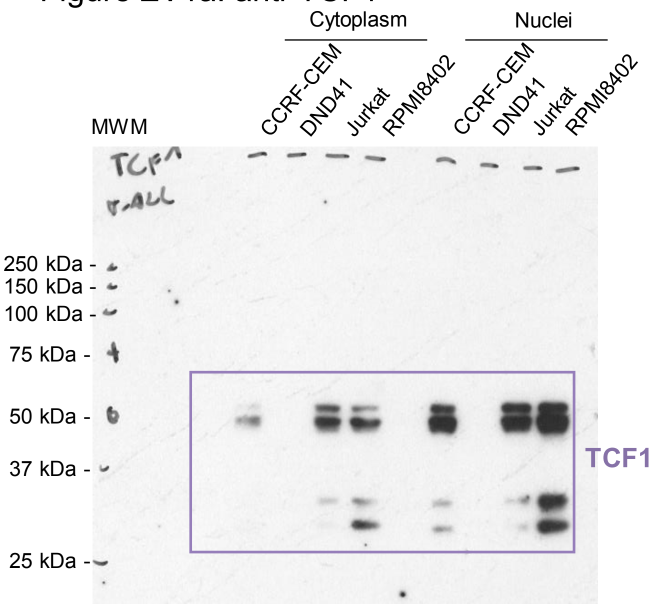

Figure EV1a: anti-LEF1

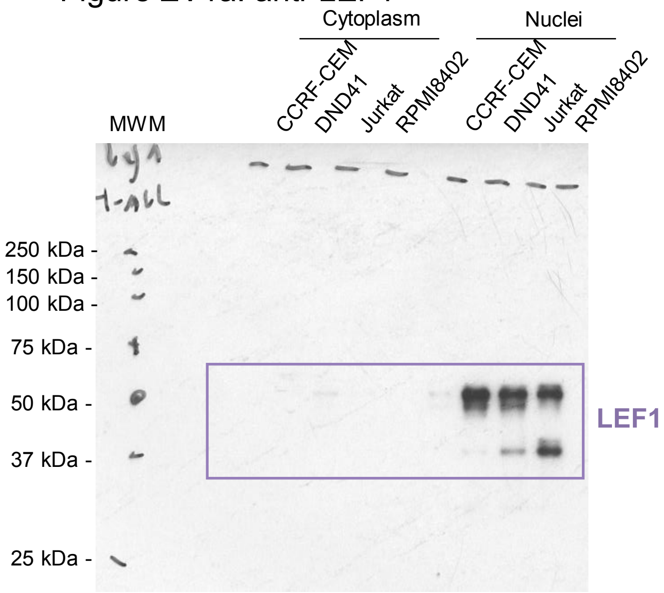

Figure EV1a: anti-Tubulin

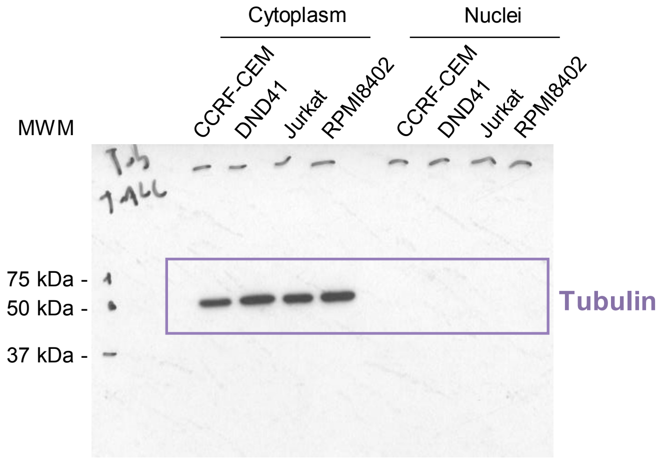

Figure EV1a: anti-Histone 3

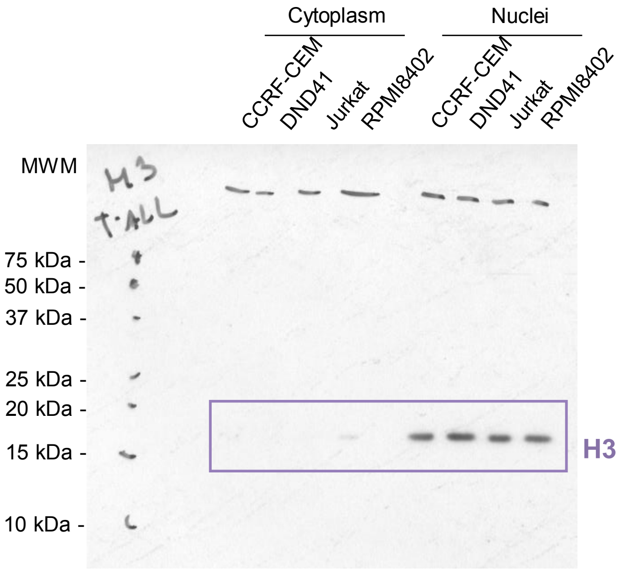

Uncropped scans of all blots in Expanded View

Figure EV1b

Figure EV1b: anti-β-catenin (low exposure)

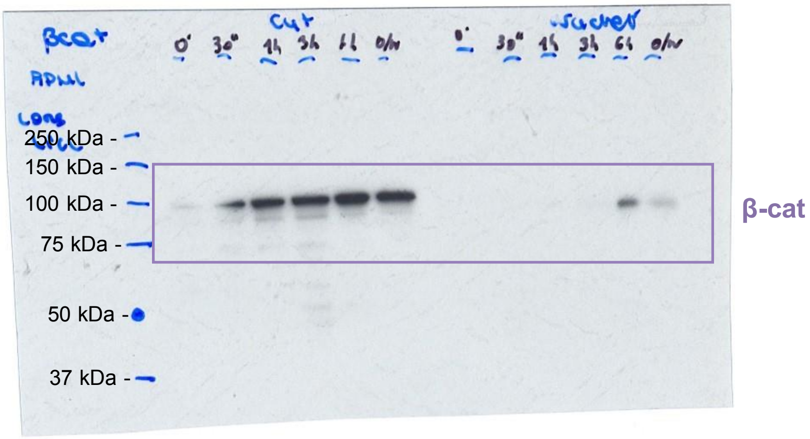

Figure EV1b: anti-β-catenin (long exposure)

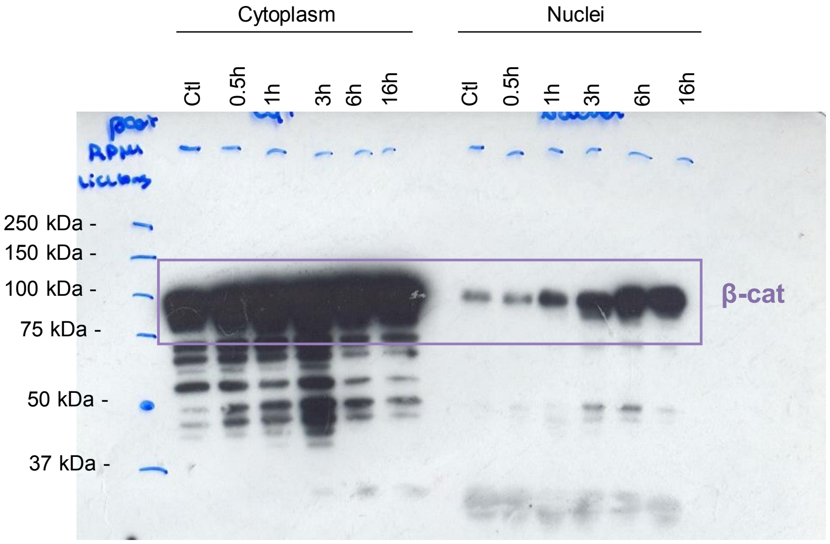

Figure EV1b: anti-Tubulin

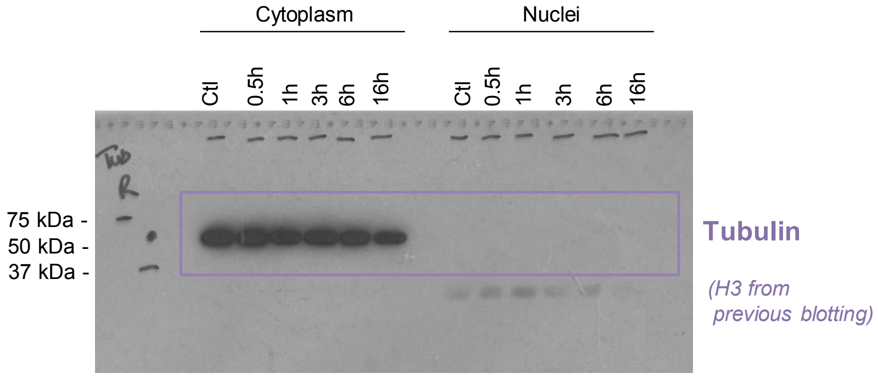

Figure EV1b: anti-Histone 3

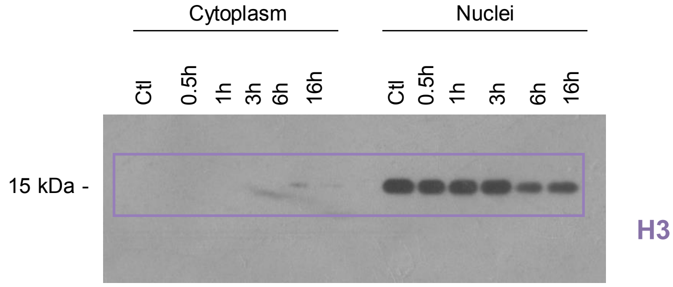

Uncropped scans of all blots in Expanded View

Figure EV1c

Figure EV1c: anti-β-catenin

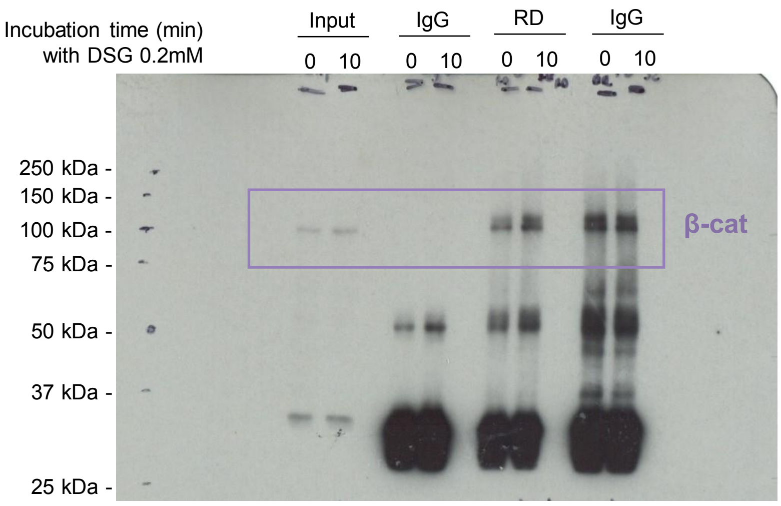

Uncropped scans of all blots in Expanded View

Figure EV1i

Figure EV1i (left): anti-Kaiso

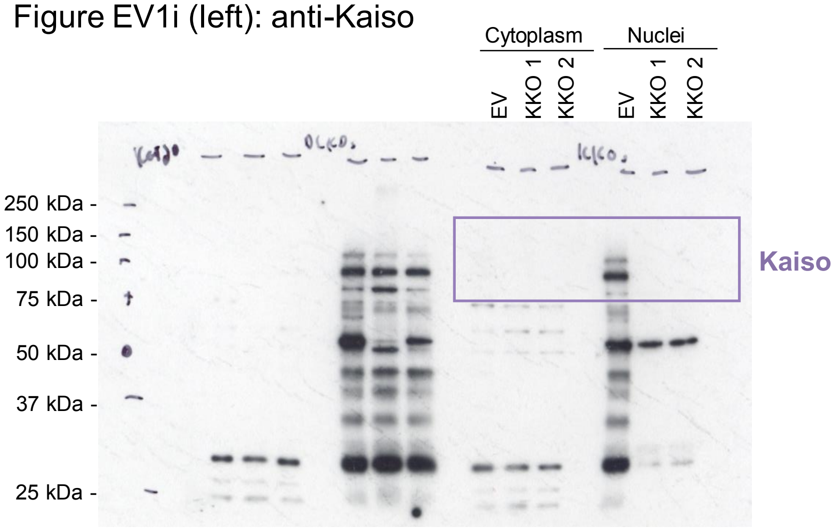

Figure EV1i (right): anti-LEF1

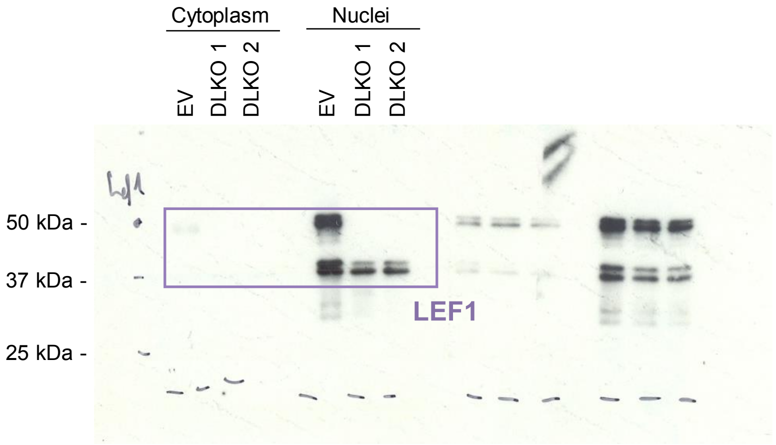

Figure EV1i (right): anti-TCF1

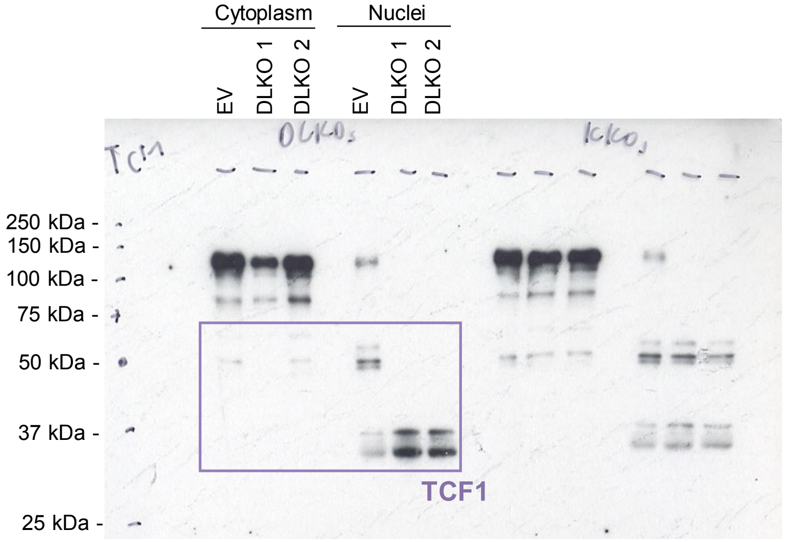

Supplement: Supplementary file 7 — Source Data for Expanded View [file EMMM-15-e16554-s003.zip › Source_data_EV1.zip!/Source_data_EV1/Fig EV1_WB_uncropped_images.pdf]
