# Supplementary material for: β‐Catenin activity induces an RNA biosynthesis program promoting therapy resistance in T‐cell acute lymphoblastic leukemia
Source: EMBO Mol Med. 2023 Jan 4;15(2):e16554. doi: 10.15252/emmm.202216554 (PMC9906382; doi:10.15252/emmm.202216554)

Uncropped scans of all blots in Expanded View

Figure EV2b

Figure EV2b (left, RPMI8402): anti-β-catenin

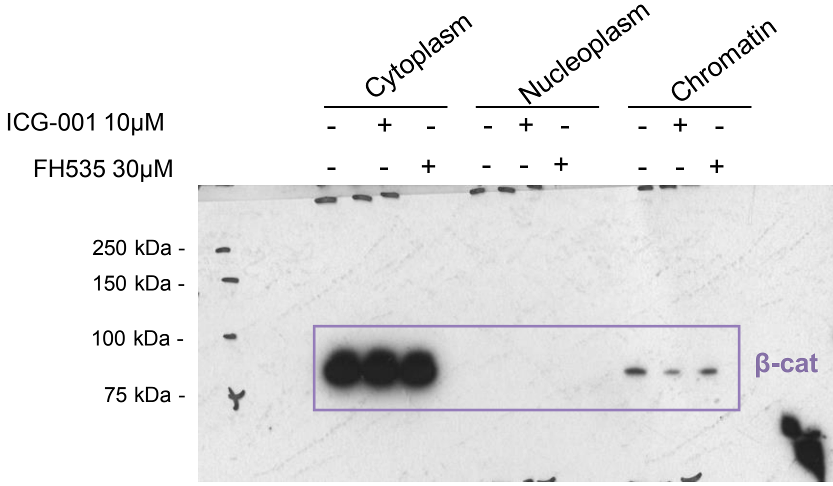

Figure EV2b (left, RPMI8402): anti-Tubulin

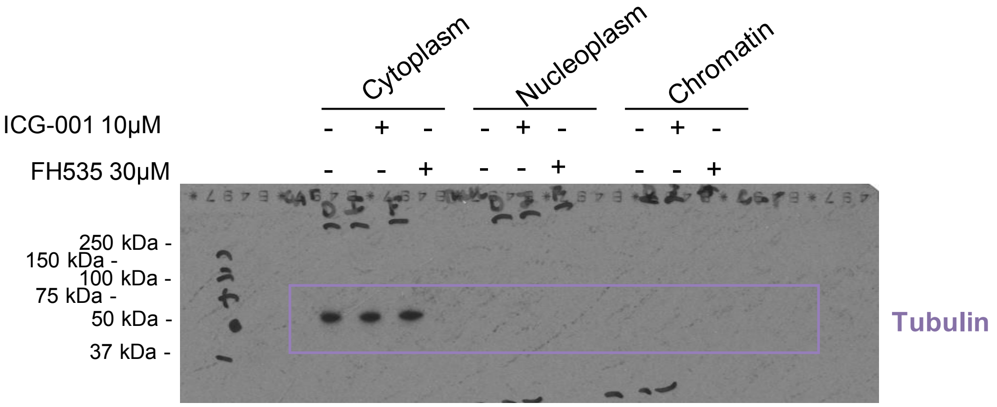

Figure EV2b (left, RPMI8402): anti-Histone 3

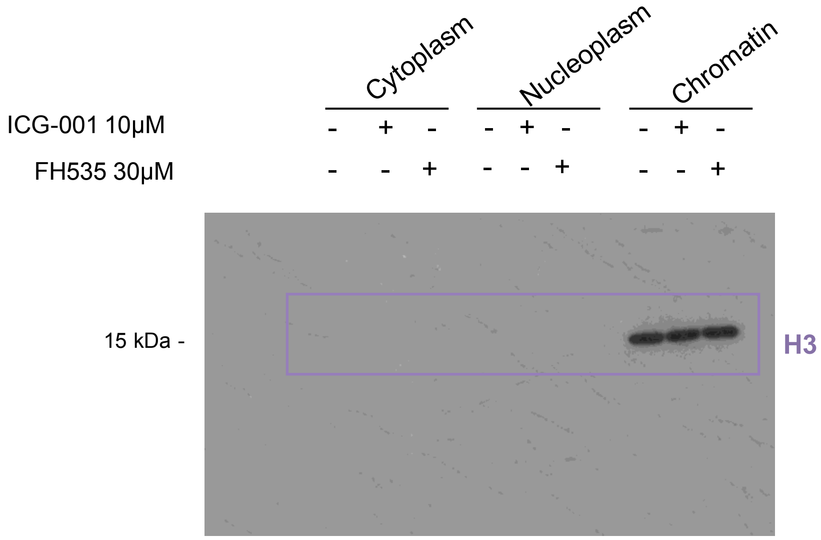

Uncropped scans of all blots in Expanded View

Figure EV2b

Figure EV2b (right, jurkat) anti-β - catenin

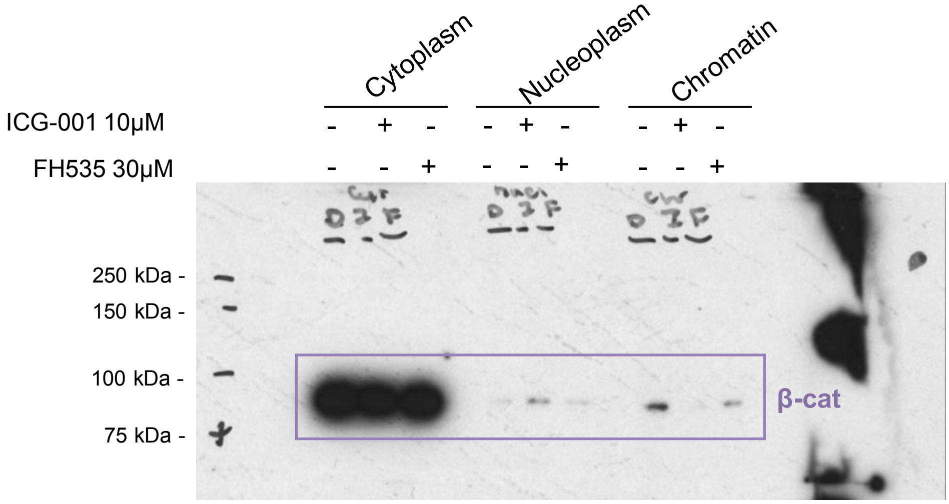

Figure EV2b (right, Jurkat): anti-Tubulin

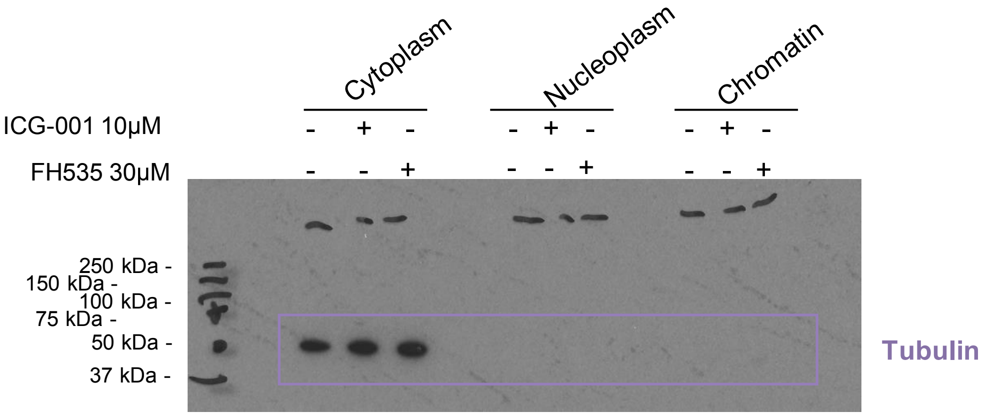

Figure EV2b (right, Jurkat): anti-Histone 3

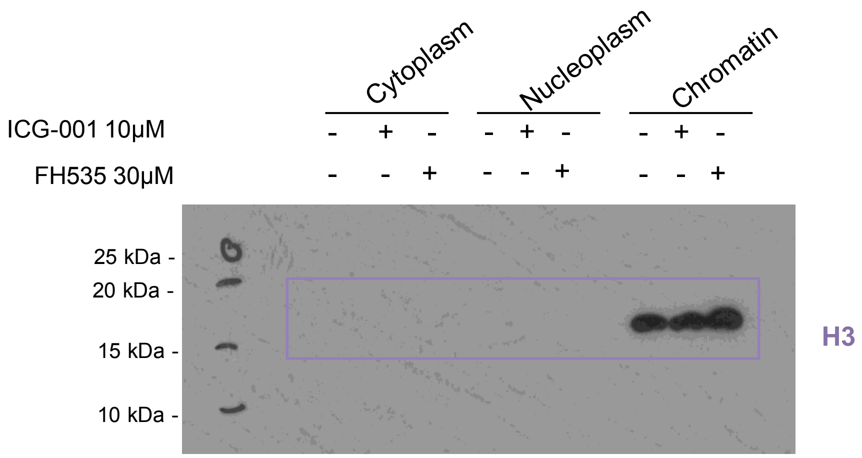

Supplement: Supplementary file 7 — Source Data for Expanded View [file EMMM-15-e16554-s003.zip › Source_data_EV2.zip!/Source_data_EV2/EV2-WB_uncropped_images1.pdf]
